# Aptamer-based multiplexed proteomic technology for biomarker discovery

**DOI:** 10.1038/npre.2010.4538.1

**Published:** 2010-06-14

**Authors:** Larry Gold, Deborah Ayers, Jennifer Bertino, Christopher Bock, Ashley Bock, Edward Brody, Jeff Carter, Virginia Cunningham, Andrew Dalby, Bruce Eaton, Tim Fitzwater, Dylan Flather, Ashley Forbes, Trudi Foreman, Cate Fowler, Bharat Gawande, Meredith Goss, Magda Gunn, Shashi Gupta, Dennis Halladay, Jim Heil, Joe Heilig, Brian Hicke, Gregory Husar, Nebojsa Janjic, Thale Jarvis, Susan Jennings, Evaldas Katilius, Tracy Keeney, Nancy Kim, Terese Kaske, Tad Koch, Stephan Kraemer, Luke Kroiss, Ngan Le, Daniel Levine, Wes Lindsey, Bridget Lollo, Wes Mayfield, Mike Mehan, Robert Mehler, Michele Nelson, Sally Nelson, Dan Nieuwlandt, Malti Nikrad, Urs Ochsner, Rachel Ostroff, Matt Otis, Thomas Parker, Steve Pietrasiewicz, Dan Resnicow, John Rohloff, Glenn Sanders, Sarah Sattin, Dan Schneider, Britta Singer, Martin Stanton, Alana Sterkel, Alex Stewart, Suzanne Stratford, Jonathan Vaught, Mike Vrkljan, Jeffrey Walker, Mike Watrobka, Sheela Waugh, Allison Weiss, Sheri Wilcox, Alexey Wolfson, Steve Wolk, Chi Zhang, Dom Zichi

**Affiliations:** 1 SomaLogic; University of Colorado at Boulder, https://www.nature.com/nature; 2 SomaLogic,; 3 Sally Jobe Breast Centre, https://www.nature.com/nature; 4 University of Colorado at Boulder,; 5 The Rogosin Institute,

**Keywords:** proteomic, aptamer, modified nucleotides, multiplex, protein, biomarker, CKD, kidney disease

## Abstract

Interrogation of the human proteome in a highly multiplexed and efficient manner remains a coveted and challenging goal in biology. We present a new aptamer-based proteomic technology for biomarker discovery capable of simultaneously measuring thousands of proteins from small sample volumes (15 [mu]L of serum or plasma). Our current assay allows us to measure ~800 proteins with very low limits of detection (1 pM average), 7 logs of overall dynamic range, and 5% average coefficient of variation. This technology is enabled by a new generation of aptamers that contain chemically modified nucleotides, which greatly expand the physicochemical diversity of the large randomized nucleic acid libraries from which the aptamers are selected. Proteins in complex matrices such as plasma are measured with a process that transforms a signature of protein concentrations into a corresponding DNA aptamer concentration signature, which is then quantified with a DNA microarray. In essence, our assay takes advantage of the dual nature of aptamers as both folded binding entities with defined shapes and unique sequences recognizable by specific hybridization probes. To demonstrate the utility of our proteomics biomarker discovery technology, we applied it to a clinical study of chronic kidney disease (CKD). We identified two well known CKD biomarkers as well as an additional 58 potential CKD biomarkers. These results demonstrate the potential utility of our technology to discover unique protein signatures characteristic of various disease states. More generally, we describe a versatile and powerful tool that allows large-scale comparison of proteome profiles among discrete populations. This unbiased and highly multiplexed search engine will enable the discovery of novel biomarkers in a manner that is unencumbered by our incomplete knowledge of biology, thereby helping to advance the next generation of evidence-based medicine.

Proteins present in blood are an immediate measure of an individual's phenotype and state of wellness. Secreted proteins, released from diseased cells and surrounding tissues, contain important biological information with the potential to transform early diagnostic, prognostic, therapeutic, and even preventative decisions in medicine.

We will realize the full power of proteomics only when we can measure and compare the proteomes of many individuals to identify biomarkers of human health and disease and track the blood-based proteome of an individual over time. Because the

Nature Precedings : hdl:10101/npre.2010.4538.1 : Posted 14 Jun 2010

human proteome contains an estimated 20,000 proteins, plus post-translational variants, that span a concentration range of ~12 logs, there is great technical difficulty in identifying and quantifying valid biomarkers. Proteomic measurements demand extreme sensitivity, specificity, dynamic range, and accurate quantification.

The desire to profile the changes in protein expression at large scale is not new. Attempts at high-content proteomics began with 2-D gels and now mostly employ mass spectrometry (MS) and antibody-based technologies[1]. MS can deliver specific analytical capabilities, but its sensitivity is limited typically to nM protein concentrations which leaves much of the proteome in plasma undetected. Techniques like Stable Isotope Standards and Capture by Anti-Peptide Antibodies (SISCAPA) and Multiple Reaction Monitoring (MRM) can be more sensitive, but are still limited to tens of protein measurements[2]. In addition, problems of cost, throughput, and reproducibility remain a challenge. Due to these limitations, MS biomarker studies cannot yet be efficiently scaled to measure with sufficient sensitivity thousands of proteins in thousands of samples, and such studies therefore miss the greatest opportunity for discovery.

In contrast to 2-D gels and MS, antibody-based methods are much more sensitive and can be used to detect analytes in the sub-nM range. This is enabled by the high affinity of antibodies for their targets which is generally in the nM to pM range. However, non-specific binding of antibodies to non-cognate proteins, other macromolecules, and surfaces requires the use of sandwich-type assays where the second antibody contributes to enhanced specificity through an independent binding event. In other words, technologies such as Enzyme-Linked Immuno-Sorbent Assays (ELISAs) attain high sensitivity by combining the specificity of two different antibodies to the same protein, requiring that both bind to elicit a signal[1]. Although broadly used in single-analyte tests, it has recently become clear that such assays cannot be multiplexed above a few tens of simultaneous measurements[3,4] in large part because cross-reactivity of secondary antibodies to surface-immobilized proteins (including primary antibodies) dramatically erodes specificity[1]. This inherent characteristic compromises the performance of antibody-based arrays including printed antibodies, sandwich formats, and bead-based arrays[1,5]. A recently reported proximity ligation assay that relies on antibody sandwich formation in solution followed by ligation of antibody-tethered nucleic acids and PCR amplification has been multiplexed with six analytes[3].

Given these challenges, we set out to develop a proteomics array technology analogous to the highly-successful nucleic acid hybridization microarray. To create this technology, we developed a new class of DNA-based aptamers enabled by a versatile chemistry technology that endows nucleotides with protein-like functional groups. These modifications greatly expand the repertoire of targets accessible to aptamers. The resulting technology provides efficient, large-scale selection of exquisite protein binding reagents selected specifically for use in highly-multiplexed proteomics arrays. Here we present the development of these unique reagents in the context of our high-content, high-performance, low-cost proteomics array, and demonstrate the potential of the platform to identify biomarkers from clinically-relevant samples.

Aptamers are a class of nucleic acid-based molecules discovered twenty years ago[6,7] and have since been employed in diverse applications including therapeutics[8], catalysis[9], and now proteomics. Aptamers are short single-stranded oligonucleotides, which fold into diverse and intricate molecular structures that bind with high affinity and specificity to proteins, peptides, and small molecules[10-12]. Aptamers are selected *in vitro* from enormously large libraries of randomized sequences by the process of Systematic Evolution of Ligands by EXponential enrichment (SELEX)[6,7]. A SELEX library with 40 random sequence positions has $4^{40}$ ($\sim10^{24}$) possible combinations and a typical selection screens $10^{14}$-$10^{15}$ unique molecules. This is on the order of $10^5$ times larger than standard peptide or protein combinatorial molecular libraries[13].

Based on the collective knowledge of the aptamer field that has developed since its inception[6,7], we hypothesized that aptamers could make exceptional reagents for high-content proteomics. There were many examples of high affinity RNA and DNA aptamers selected against human proteins[12]. However, there were also examples of difficult protein targets for which standard RNA and DNA SELEX did not yield high affinity aptamers. With two key innovations, we created a new class of aptamer, the Slow Off-rate Modified Aptamer (SOMAmer), which enabled efficient selection of high-affinity aptamers for almost any protein target.

The first innovation was motivated by the idea that aptamers can be endowed with protein-like properties by adding functional groups that mimic amino acid side-

Nature Precedings : hdl:10101/npre.2010.4538.1 : Posted 14 Jun 2010

chains to expand their chemical diversity[14]. Eaton and colleagues developed the technology to efficiently synthesize nucleotides modified with diverse functional groups and to utilize them in SELEX[14,15]. This innovation was used to select catalysts, including the first RNA-catalyzed carbon-carbon bond formation[9,16]. Building on this work, we developed modified deoxyribonucleotides and SELEX methods[17] to select modified DNA aptamers from libraries that incorporate one of four dUTPs modified at the 5-position (Fig. 1a and Supplementary Information (SI)).

To test whether modified nucleotides improve SELEX, we compared selections with modified and unmodified nucleotides targeting thirteen "difficult" human proteins that repeatedly failed SELEX with unmodified DNA. As a control, we included GA733-1 protein, which had yielded high-affinity aptamers with unmodified DNA SELEX. The results (Supplementary Table 1) show that only SELEX with modified nucleotides yielded high-affinity aptamers to these difficult proteins. It is worth noting that, depending on the protein, certain modifications worked better than others (Supplementary Table 1), illustrating the benefit of applying multiple modifications against the same target to ensure a high probability of success. Based on these results, we adopted modified nucleotide SELEX exclusively in our standard selections. To date, we have selected high-affinity aptamers (with most $K_d$ values lower than nM, see Fig. 1b) to over 1000 human proteins, nearly all the proteins we have targeted. There are no obvious commonalities among those proteins that were initially unsuccessful in SELEX with unmodified DNA. Overall, these results provide the first comprehensive evidence that modified nucleotides can expand the range of possible aptamer targets and improve their binding properties.

The second innovation was a solution to the principal challenge of identifying a second element of specificity beyond binding of a second ligand for use in high-content arrays. Inspired by classic kinetic theory of specific binding in complex mixtures[18,19], we employed kinetic manipulations to help overcome the problem of non-specific SOMAmer-protein binding. To achieve this second element of specificity, we selected for aptamers with slow dissociation rates ($t_{1/2}$ >30 min, Fig. 1c) that allow selective disruption of non-specific (or non-cognate) binding interactions by using a large excess of a polyanionic competitor. This kinetic challenge works well for two reasons. First, dissociation rates of non-cognate SOMAmer-protein interactions are generally much

Nature Precedings : hdl:10101/npre.2010.4538.1 : Posted 14 Jun 2010

faster (half-lives of a few minutes or less). Second, since all aptamers are polyanions, another polyanion at high concentration (*e.g.*, dextran sulfate) can serve as a common competitor that dramatically minimizes rebinding events in a multiplex assay. In contrast, a common non-denaturing competitor of all antibody-antigen interactions or, more generally, protein-protein interactions, is not known.

The current array measures 813 human proteins (Supplementary Table 2). These proteins represent a wide range of sizes, physicochemical properties (*e.g.*, pI range of 4-11 as shown in Fig. 1d), and biological functions from a variety of molecular pathways and gene families (Fig. 1e). Thus, SOMAmer technology enables an efficient and scalable pipeline to generate unbiased content for proteomics arrays.

To create our high-content proteomics discovery array, we developed a novel assay (Fig. 2) which transforms a complex proteomic sample (*e.g.*, plasma, serum, conditioned media, cell lysates, *etc.*) into a quantified protein signature. The assay leverages equilibrium binding and kinetic challenge[1]. Both are carried out in solution, not on a surface, to take advantage of more favorable kinetics of binding and dissociation[1]. Briefly, the sample is incubated with a mixture of SOMAmers each containing a biotin, a photocleavable group, and a fluorescent tag followed by capture of all SOMAmer-protein complexes on streptavidin beads (catch-1) (Fig. 2a, 2b-1,2). After stringent washing of the beads to remove unbound proteins and labeling of bead-associated proteins with biotin under controlled conditions (Fig. 2b-3), the complexes are released from the beads back into solution by UV light irradiation and diluted into a high concentration of dextran sulfate, an anionic competitor. Note that the biotin that was originally part of the SOMAmer now remains on beads. The anionic competitor coupled with dilution selectively disrupts non-cognate complexes (see Fig. 3a) and since only the proteins now contain biotin, the complexes are re-captured on a second set of beads (catch-2) from which unbound SOMAmers are removed by a second stringent washing (Fig. 2b-5). The SOMAmers that remain attached to beads are eluted under high pH-denaturing conditions and hybridized to sequence-specific complementary probes printed on a standard DNA microarray (Fig. 2b-6,7).

The result is a mixture of SOMAmers that quantitatively reflects protein concentrations in the original sample. The modified nucleotides in SOMAmers are designed to maintain canonical base-pairing[17,20] (in a DNA duplex, adducts at the 5-

Nature Precedings : hdl:10101/npre.2010.4538.1 : Posted 14 Jun 2010

position of pyrimidines are directed toward the major groove of DNA) and hybridize effectively to unmodified DNA oligonucleotides on the array (this, of course, is also required for replication during SELEX). Thus, our assay takes advantage of the dual nature of aptamers as molecules capable of both folding into complex three-dimensional structures, which is the basis of their unique binding properties, and hybridization to specific capture probes.

The assay uses one SOMAmer per analyte rather than a sandwich of binding reagents and thus depends on equilibrium binding and kinetics for specificity. A key contribution to specificity in the assay is the difference in dissociation rates between cognate and non-cognate interactions as illustrated in Fig. 3a. For example, the half-life of dissociation of kallistatin, LBP, and TIG2 SOMAmers from their cognate targets (determined by using unlabeled SOMAmers) are 65, 44, and 65 minutes, compared to <1 minute for dissociation of the same SOMAmers from histone H1.2, a known DNA binding protein. Specific interactions are disrupted to a far lesser degree by dextran sulfate for all three SOMAmers (Fig. 3a). This translates to substantial enrichment in the specific signal following kinetic challenge and the two-bead capture steps.

It is worth noting that the use of sequential capture of protein-SOMAmer complexes on two sets of streptavidin beads, first through biotin-labeled SOMAmers (catch-1) and then through biotin-labeled proteins (catch-2), substantially reduces non-specific interactions. As shown in Fig. 3b, eluate from catch-1 beads generally contains the target protein as well as several other proteins that bind SOMAmers non-specifically. Eluate from catch-2 beads contains only the target protein in substantially pure form, along with its cognate SOMAmer (for these experiments, reversible protein attachment to monomeric avidin catch-2 beads is used) (SI). This is likely due, in part, to a reduction in the amount of total protein following catch-1 bead washing (only SOMAmer-bound or surface-bound proteins remain) as well as to release and recapture of complexes on separate beads in a reversed orientation (attachment through biotin on proteins).

Finally, capture of SOMAmers on a hybridization array permits quantitative determination of the protein present in the original sample by converting the assay signal (relative fluorescence units, RFUs) to analyte concentration (Fig. 3c). These

Nature Precedings : hdl:10101/npre.2010.4538.1 : Posted 14 Jun 2010

results show that specific SOMAmer-protein interactions can be detected efficiently in highly complex mixtures like serum or plasma.

With this format, we achieved our goal of developing a high-content, high-performance proteomics technology to power biomarker discovery in human disease. To assess the quantitative performance of the technology, we determined reproducibility and limits of quantification (LOQ). The assay analyzes 96 samples per run. We collected serum samples from 18 healthy volunteers and assayed five replicates of each sample in a single run and repeated this three times. The results show an overall low median CV of ~5% for intra-run and inter-run CV. We also determined the LOQ values of a representative subset (356) of target proteins in the context of all 813 SOMAmers (SI). The median lower limit of quantification (LLOQ) was ~1 pM, with LLOQs as low as 100 fM for some proteins, a median upper limit of quantification (ULOQ) of ~1.5 nM, and a median range of quantification (ROQ) of >3 logs. We found consistent performance in serum for proteins with low endogenous concentrations when titrated into 10% serum and plasma (SI). Overall, we achieve an ROQ for all proteins in a sample of ~7 logs (~100 fM – 1 μM) with three sample dilutions that span ~2.5 logs. The content of the discovery array is flexible and highly scalable, permitting us to continue adding content as our SOMAmer menu increases. This highly multiplexed technology therefore has the requisite reproducibility, sensitivity and range for high-content proteomics studies and unbiased biomarker discovery.

To demonstrate the utility of the platform in discovery of disease-related biomarkers, we analyzed plasma from subjects with chronic kidney disease (CKD), the slow loss of kidney function over time. CKD is a recently recognized global public health problem that is "common, harmful, and treatable" with an estimated prevalence of nearly 10% worldwide[21]. Early intervention in CKD can substantially improve prognosis, which is otherwise poor[21-24]. To achieve early diagnosis, predictive, and non-invasive CKD biomarkers are needed. Such markers also would be useful for monitoring disease progression and guiding treatment[21-24].

We chose CKD as a test case because kidney physiology provides filtration of serum molecules based on size (molecular mass) and charge[25] – thus CKD might lead to an increase in the concentration of small proteins (MW <45 kDa). Disease progression

Nature Precedings : hdl:10101/npre.2010.4538.1 : Posted 14 Jun 2010

is expected to be accompanied by an overall increase in plasma concentration of small proteins.

We obtained and analyzed plasma samples from 42 subjects with CKD. Eleven subjects had early-stage CKD based on estimated GFR (eGFR, defined as stages 1-2, median creatinine clearance 70 ml/min/m$^2$, range 62-97 ml/min/m$^2$) and 31 had late-stage CKD (stages 3-5, median creatinine clearance 25 ml/min/m$^2$, range 7-49 ml/min/m$^2$)[26]. We measured 614 human proteins (array size at the time analyses were conducted) simultaneously for each sample and compared the results of early- to late-stage CKD (Fig. 4a).

We identified 60 proteins that varied significantly between the two groups, using the Mann-Whitney test, with a q-value (false discovery rate-corrected p-value) of $4.2 \times 10^{-4}$ (Supplementary Table 10). Eleven proteins with the most highly significant variation (q-values $<3.5 \times 10^{-7}$) are highlighted in Fig. 4a and shown in Table 1. Nine out of eleven are relatively small proteins (<25 kDa). For all eleven proteins, there is an inverse correlation between eGFR and protein concentration (Fig. 4b), which supports the notion that these proteins are biomarkers for CKD progression. It is also worth noting that two of the eleven proteins, cystatin C and $\beta_2$-microglobulin, are important known biomarkers of CKD[22-24] and two additional proteins, complement factor D and TNF sR-I, have been reported to have elevated concentrations in CKD[27,2828,29].

Accumulation in plasma of some small proteins appears to be a major change in the proteome. However, the concentration of many low molecular weight proteins did not change appreciably with disease progression (Fig. 4c); pI also was uncorrelated with an increase in plasma concentration as a function eGFR (data not shown). The surprising fact that the biomarkers are not simply ranked according to their molecular masses shows that reduced kidney function is complex. The accumulation of some (but not all) low molecular weight proteins, sometimes called "middle molecules", in plasma of patients with impaired renal filtration has long been implicated in the pathology of kidney disease[29]. High-content proteomic analysis provides a means of unbiased discovery of such proteins and their relationship to disease progression..

This example demonstrates our ability to discover biomarkers to build diagnostic signatures of disease states for which there is an important medical need[21-24].

Nature Precedings : hdl:10101/npre.2010.4538.1 : Posted 14 Jun 2010

Combining multiple biomarkers might create a high resolution picture of CKD to help develop diagnostic tools.

In conclusion, we have presented the first highly multiplexed and efficient aptamer-based proteomics array technology that simultaneously measures large numbers of proteins ranging from low to high abundance in serum. In CKD, we have identified a multitude of biomarkers with large differences in concentration between early- and late-stage disease. Therefore, these biomarkers represent good candidates for use (alone or in combination) in diagnostic tests for CKD progression. A study of more than 500 additional patients at risk for cardiovascular disease (whose eGFRs were also determined) confirmed and extended the biomarkers associated with reduced filtration in this first CKD study (data not shown).

We have also conducted clinical studies in which no biomarkers have emerged. For example, in our prospective multicenter breast cancer study, we compared plasma proteomic signatures measuring 813 proteins (current array) of 336 women with suspicious mammogram findings. Based on breast biopsy results, 32 women had ductal carcinoma *in situ*, 57 had invasive breast cancer and 247 had benign disease. There were no statistically significant differences in the proteomic profile among these three groups. These results, while disappointing, demonstrate that biomarkers are not identified by chance merely because thousands of measurements are made. Of course, it is possible that with a larger array, new biomarkers of breast cancer will emerge.

Our experience to date suggests that in most cases, biomarker discovery using our technology lies someplace between these extremes, with potentially useful biomarkers identified in many critical medical areas including cancer, cardiovascular conditions, neurological disorders, and infectious diseases. Frequently, the distribution of biomarker concentrations among two populations contains considerable overlap which creates the impetus for combining multiple biomarkers to achieve the most accurate diagnosis. In an accompanying paper[30], we report the first large-scale application of our technology to discover and verify a novel biomarker panel for a major important medical condition, lung cancer, in one of the largest and most comprehensive proteomic biomarker studies to date.

Nature Precedings : hdl:10101/npre.2010.4538.1 : Posted 14 Jun 2010

### METHODS SUMMARY

We used rationally-designed, chemically-modified nucleotides to create a new class of aptamer – Slow Offrate Modified Aptamers (SOMAmers) – to use as protein binding reagents. We developed new SELEX methods, selected high affinity SOMAmers for >800 human proteins, and developed a highly multiplexed protein affinity assay that uses standard DNA quantification technologies as the final readout. We used affinity capture to demonstrate the specificity of SOMAmers for their target proteins. Assay reproducibility was measured with multiple technical replicates of serum and plasma. We determined the limits and range of quantification of target proteins with six-point, multiplexed standard curves of purified proteins in buffer. To demonstrate the utility of our new proteomics platform to discover potential biomarkers, we profiled 614 proteins in plasma samples from subjects with early-stage and late-stage CKD. The final readout was a custom DNA microarray. We compared the resulting measurements with the non-parametric Mann-Whitney U Test because the data were non-normally distributed ordinal variables. For significance, we used an alpha cutoff of $4.3 \times 10^{-04}$ for the q-value (p-value corrected for multiple comparisons using a false discovery rate estimate). Sixty of the 614 measured proteins were significantly different between early- and late-stage CKD subjects.

**Full Methods** and any associated references are available in the Supplementary Information.

**Supplementary Information** is linked to the online version of the paper at www.nature.com/nature.

Nature Precedings : hdl:10101/npre.2010.4538.1 : Posted 14 Jun 2010

**Acknowledgments**

We thank all our colleagues in the genesis and evolution of aptamer science and developing aptamer-based biotechnology over the past decades. They contributed immeasurably with countless insights about aptamers and the nature of biology. We especially thank past and present colleagues at the University of Colorado, NeXstar Pharmaceuticals, and SomaLogic.

Nature Precedings : hdl:10101/npre.2010.4538.1 : Posted 14 Jun 2010

**Author Contributions**

All authors contributed extensively to the work presented in this paper. D.L. and T.P. provided CKD samples and critically evaluated results. E.B., V.C., T.K., and R.O. conducted the prospective breast cancer study. N.J., J.J.W., S.K.W., and D.Z. wrote the manuscript with input from the entire team.

**Author Information**

Correspondence and requests for materials should be addressed to L.G. (lgold@somalogic.com) or J.J.W. (jwalker@somalogic.com).

### Tables

**Table 1. Top 11 Potential CKD Biomarkers [a]**

| Target | p-value | q-value | Mol. Mass (kDa) |
|---|---|---|---|
| $\beta_2$-Microglobulin | $1.2 \times 10^{-9}$ | $8.0 \times 10^{-8}$ | 11.7 |
| FSTL3 | $1.2 \times 10^{-9}$ | $8.0 \times 10^{-8}$ | 25.0 |
| Pleotrophin | $1.2 \times 10^{-9}$ | $8.0 \times 10^{-8}$ | 15.3 |
| TNF sR-I [b, c] | $1.2 \times 10^{-9}$ | $8.0 \times 10^{-8}$ | 21.2 |
| Factor D | $4.8 \times 10^{-9}$ | $2.1 \times 10^{-7}$ | 24.4 |
| IL-15 Rα [b, d] | $4.8 \times 10^{-9}$ | $2.1 \times 10^{-7}$ | 25.0 |
| MMP-7 | $8.4 \times 10^{-9}$ | $3.2 \times 10^{-7}$ | 19.1 |
| Angiopoietin-2 | $1.4 \times 10^{-8}$ | $3.5 \times 10^{-7}$ | 54.9 |
| Cystatin C | $1.4 \times 10^{-8}$ | $3.5 \times 10^{-7}$ | 13.3 |
| HCC-1[b] | $1.4 \times 10^{-8}$ | $3.5 \times 10^{-7}$ | 8.7 |
| URB [b] | $1.4 \times 10^{-8}$ | $3.5 \times 10^{-7}$ | 105.7 |

[a] Based on q-value ranking

[b] Smaller isoforms also exist.  For example, URB has a 10.3 kDa isoform

[c] Extracellular domain comprising amino acids 22-211

[d] Extracellular domain is 18.4 kDa

Nature Precedings : hdl:10101/npre.2010.4538.1 : Posted 14 Jun 2010

Nature Precedings : hdl:10101/npre.2010.4538.1 : Posted 14 Jun 2010

### FIGURES

Figure 1a

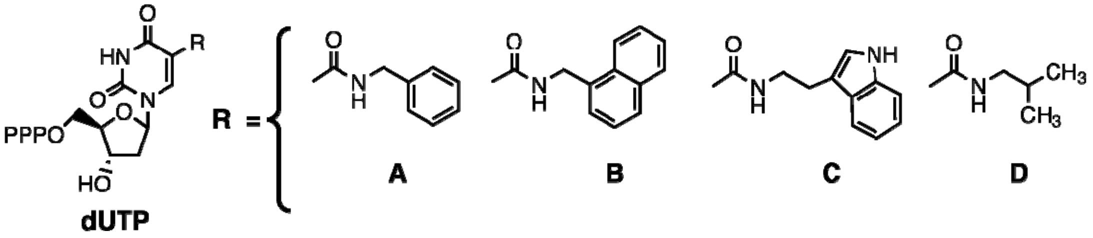

Figure 1b

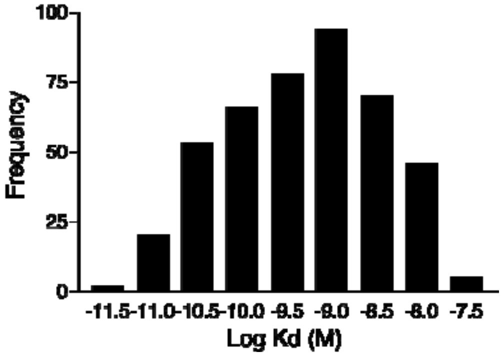

Figure 1c

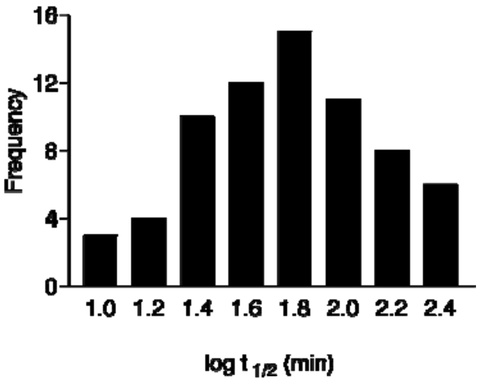

Figure 1d

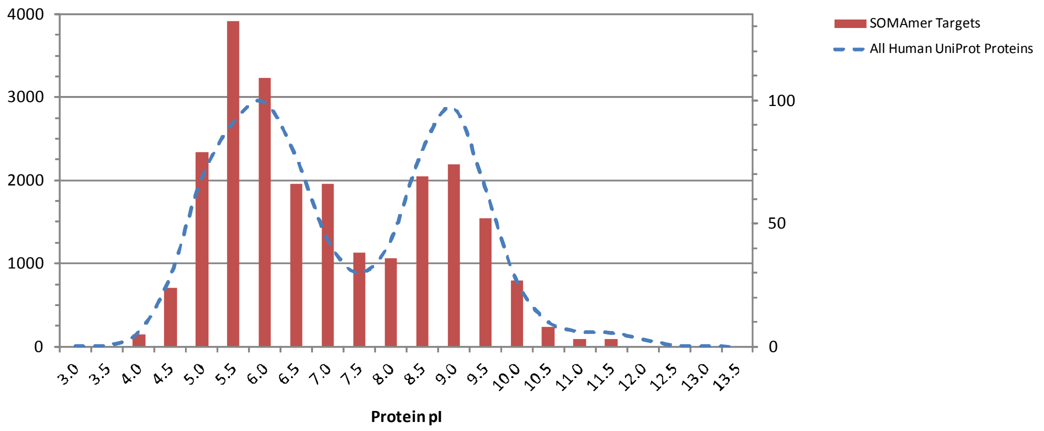

Figure 1e

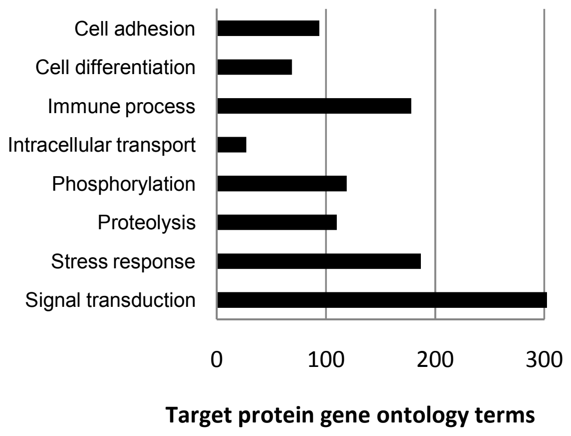

Nature Precedings : hdl:10101/npre.2010.4538.1 : Posted 14 Jun 2010

Nature Precedings : hdl:10101/npre.2010.4538.1 : Posted 14 Jun 2010

Figure 2

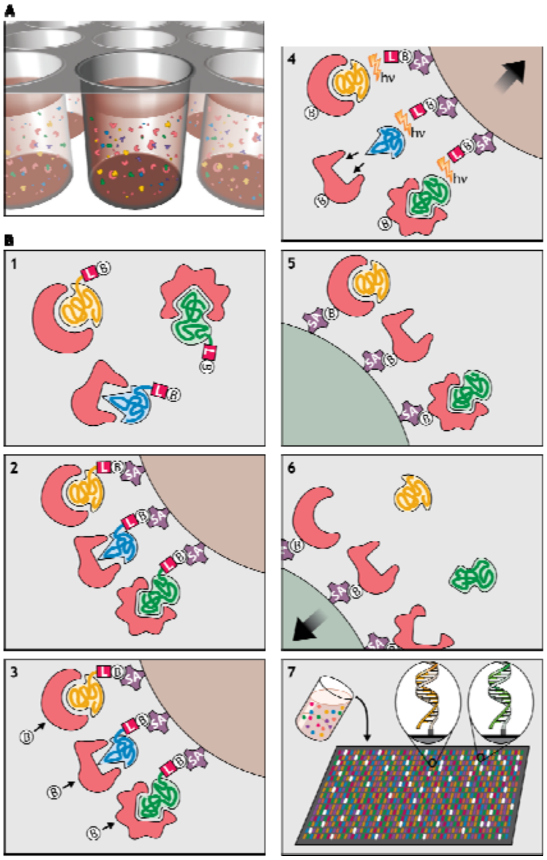

Figure 3a

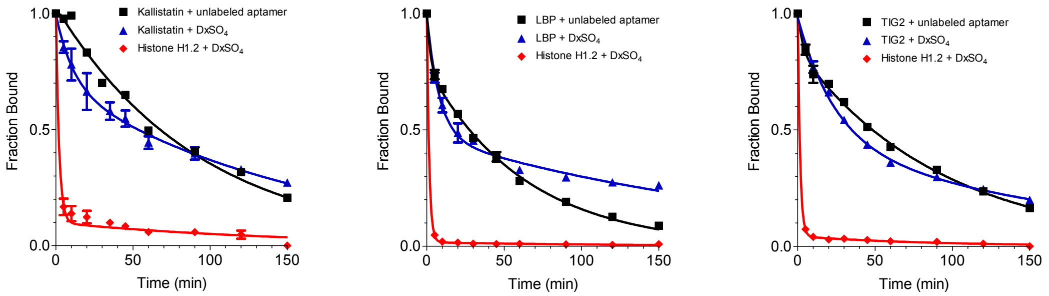

Figure 3b

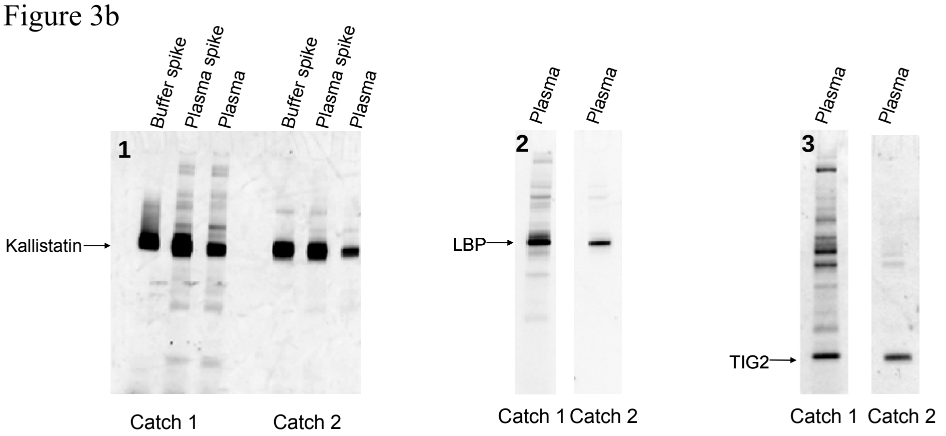

Figure 3c

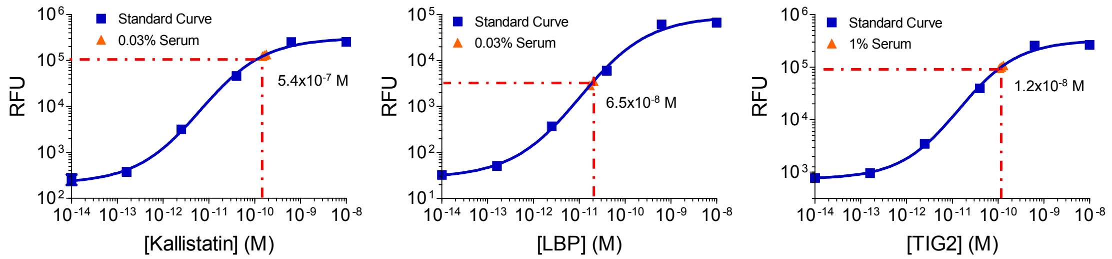

Nature Precedings : hdl:10101/npre.2010.4538.1 : Posted 14 Jun 2010

Figure 4a

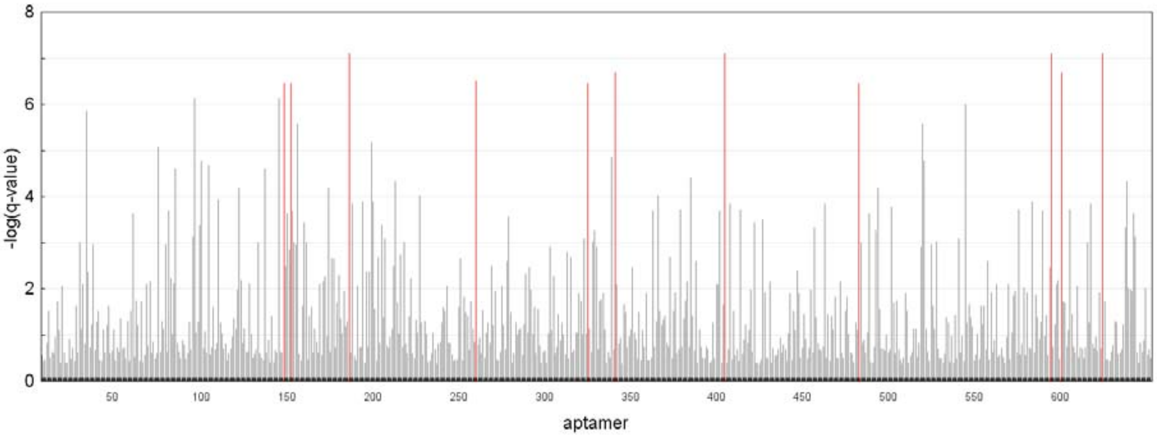

Figure 4b

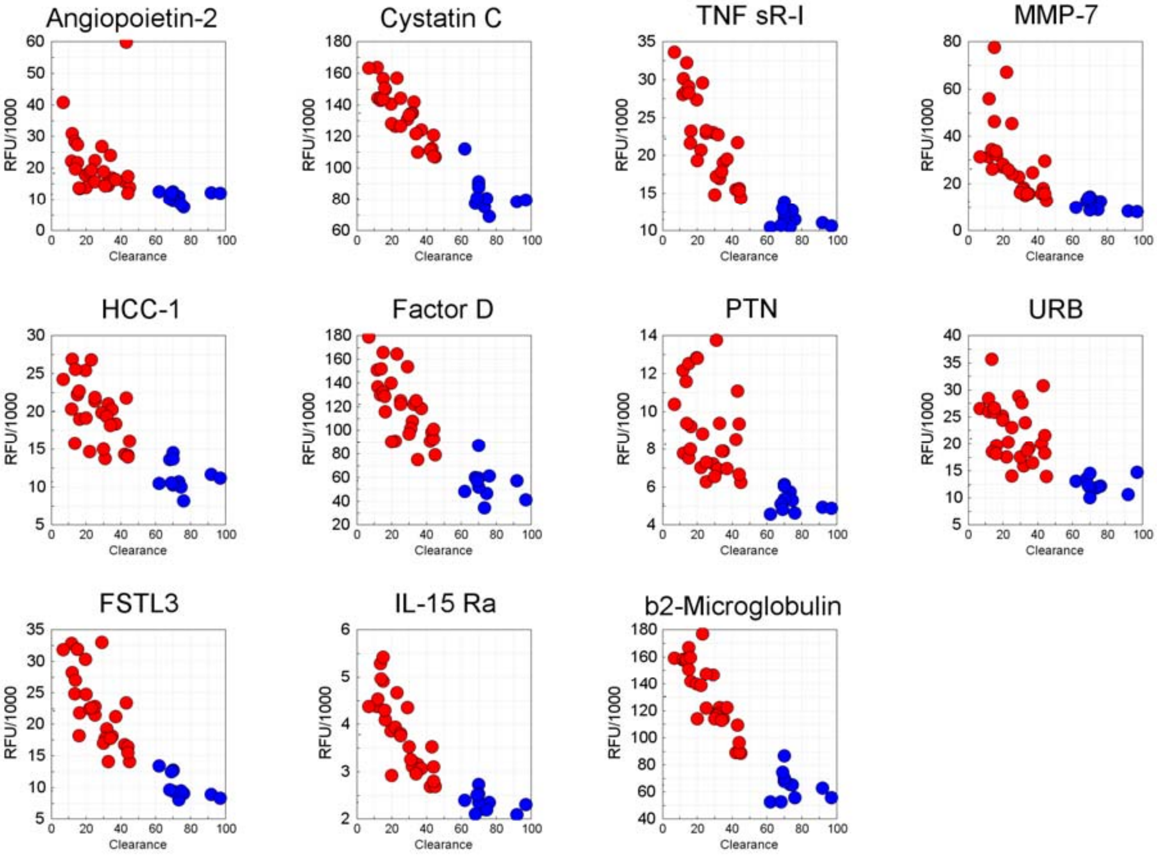

Nature Precedings : hdl:10101/npre.2010.4538.1 : Posted 14 Jun 2010

Figure 4c

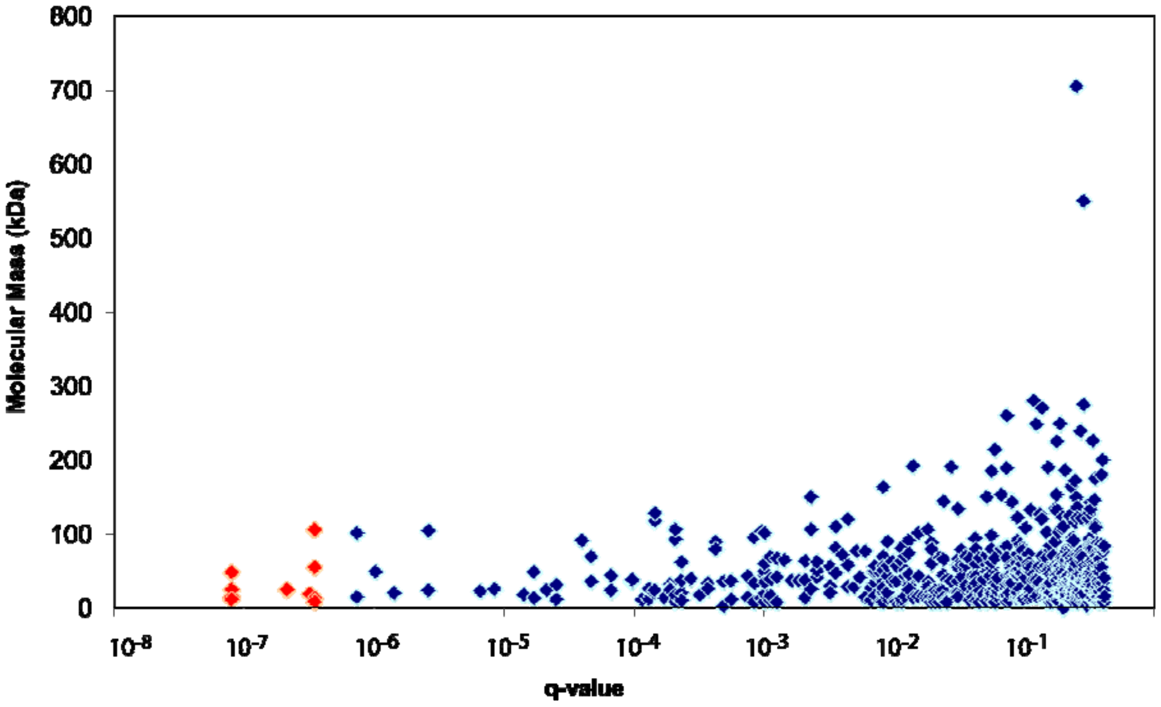

### FIGURE LEGENDS

**Figure 1. Unique properties of SOMAmers. a,** Nucleotide triphosphate analogs modified at the 5-position (R) of uridine (dUTP). The 5-position modifications include: (A) 5-benzylaminocarbonyl-dU (BndU); (B) 5-naphthylmethylaminocarbonyl-dU (NapdU): (C) 5-tryptaminocarbonyl-dU (TrpdU); and (D) 5-isobutylaminocarbonyl-dU (iBudU). **b,** Distribution of dissociation constant ($K_d$) values for 434 SOMAmers. **c,** Distribution of dissociation rate constant ($k_d$) values for 72 SOMAmers representative of those in proteomic arrays. **d,** Distribution of isoelectric points (pI) of proteins for which SOMAmers have been selected (solid red bars) and of all human protein chains in UniProt (dashed blue line). **e,** Distribution of most common gene ontology terms associated with the proteins measured by the current array.

Nature Precedings : hdl:10101/npre.2010.4538.1 : Posted 14 Jun 2010

Nature Precedings : hdl:10101/npre.2010.4538.1 : Posted 14 Jun 2010

**Figure 2**. **Principle of multiplex SOMAmer affinity assay. a,** Binding. SOMAmers and samples are mixed in 96-well micro-well plates and allowed to bind. Cognate and non-cognate SOMAmer-target protein complexes form. Free SOMAmer and protein are also present. **b,** Schematic sequence of assay steps leading to quantitative readout of target proteins. (1) SOMAmer-protein binding: DNA-based SOMAmer molecules (gold, blue, and green) have unique shapes selected to bind to a specific protein. SOMAmers contain biotin (B), a photo-cleavable linker (L) and a fluorescent tag at the 5' end. Most SOMAmers (gold and green) bind to cognate proteins (red), but some (blue) form non-cognate complexes. (2) Catch-1. SOMAmers are captured onto a bead coated with streptavidin (SA) which binds biotin. Un-complexed proteins are washed away. (3) Proteins are tagged with NHS-biotin. (4) Photocleavage and kinetic challenge. UV light (hv) cleaves the linker and SOMAmers are released from beads, leaving biotin on bead. Samples are challenged with anionic competitor (dextran sulfate). Non-cognate complexes (blue SOMAmer) preferentially dissociate. (5) Catch-2 SOMAmer-protein complexes are captured onto new avidin coated beads by protein biotin tag. Free SOMAmers are washed away. (6) SOMAmers are released from complexes into solution at high pH. (7) Remaining SOMAmers are quantified by hybridization to microarray containing single-stranded DNA probes complementary to SOMAmer DNA sequence, which form a double-stranded helix. Hybridized SOMAmers are detected by fluorescent tags when the array is scanned.

**Figure 3**. **Kinetic discrimination between cognate and non-cognate interactions. a,** Dissociation rate measurements for specific and non-specific protein interactions with representative Kallistatin, LBP, and TIG2 SOMAmers. Histone H1.2 binds to random

DNA sequences and was used to demonstrate non-specific binding. The fraction of radiolabeled SOMAmer (10 pM) bound to its cognate target is shown after addition of 50 nM unlabeled SOMAmer (■) or 0.3 mM dextran sulfate (▲) as a function of time. Rapid dissociation of non-specific complexes in the presence of 0.3 mM dextran sulfate is also shown. **b,** SDS-PAGE visualization of representative proteins bound by the SOMAmers selected against Kallistatin, LBP and TIG2. Gel 1 shows proteins bound to the Kallistatin SOMAmer for target added to buffer (lane 1), target added to 10% plasma (lane 2), and 10% plasma alone (lane 3). The first set of three lanes demonstrates all of the proteins eluted from catch-1 beads. The second set of lanes shows the SOMAmer-bound proteins eluted from catch-2 beads. Gels 2 and 3 demonstrate proteins recovered from 10% plasma using the LBP and TIG2 SOMAmers, respectively (without adding proteins to plasma for these three gels). The endogenous plasma proteins captured by the Kallistatin, LBP, and TIG2 SOMAmers were identified by LC-MS/MS as the intended target proteins (data not shown). **c,** Proteomics assay data for kallistatin, LBP and TIG2. Each plot shows the standard curve with error bars for eight replicates of target spiked into buffer (blue symbols). Triplicate measurements from diluted normal serum (red symbols) are plotted onto the standard curve, and the calculated normal concentrations in 100% serum are shown.

**Figure 4. Biomarker discovery in CKD. a**, Distribution of the false discovery rate (q-value) for the Mann-Whitney test statistic comparing late-stage *vs.* early-stage CKD for each protein measured (indicated as a bar on the x-axis) ordered as they appear on the array. Eleven analytes with the smallest q-values ($<3.5 \times 10^{-7}$) are shown in red bars. **b**, Protein concentrations (expressed as RFU values) as a function of renal clearance for

Nature Precedings : hdl:10101/npre.2010.4538.1 : Posted 14 Jun 2010

the eleven best biomarkers of late-stage (red symbols) *vs.* early-stage CKD (blue symbols). **c**, Comparison of a protein's molecular mass and the probability that it is a CKD biomarker (q-value (p-value corrected for false discovery rate)).

Nature Precedings : hdl:10101/npre.2010.4538.1 : Posted 14 Jun 2010

Nature Precedings : hdl:10101/npre.2010.4538.1 : Posted 14 Jun 2010

## Supplementary Information

### Aptamer-based multiplexed proteomic technology for biomarker discovery

## SUPPLEMENTARY RESULTS

**SELEX with modified nucleotides**

In order to select SOMAmers with the novel modified nucleotides described above, we developed new methods to incorporate modified nucleotides into the SELEX process. This included the synthesis of random libraries with modified nucleotides and the enzymatic amplification of SELEX pools that contain modified nucleotides. Several enzymes were screened for the ability to incorporate these modified nucleotides, as well as to amplify a modified template. We used *Thermococcus kodakaraensis* (KOD) DNA polymerase for PCR with a slightly modified buffer, although at low efficiency. Additionally, conditions have been defined to amplify selected DNA using a two-step process to avoid potential amplification biases. These methods are detailed in the Materials and Methods section below.

To test whether modified nucleotides improve SELEX to human proteins, we compared selections with modified and unmodified nucleotides against thirteen difficult human proteins for which unmodified SELEX had failed. As a control, we included a protein that previously yielded high-affinity SOMAmers with unmodified SELEX. The results of this experiment are shown in Table 1. Only SELEX with modified nucleotides yielded high-affinity SOMAmers to the difficult proteins.

We have selected SOMAmers to > 1000 human proteins. We first implemented 5-benzylaminocarbonyl-dU (BndU) in our high-throughput SELEX pipeline, and our success rate for selections to human proteins rose from < 30% to > 50% to a diversity of human proteins. This supported our hypothesis that we could develop one SELEX protocol that would work repeatedly for very different proteins. Since then, we have incorporated four modified nucleotides, BndU, 5-naphthylmethylaminocarbonyl-dU (NapdU), 5-tryptaminocarbonyl-dU (TrpdU), 5-isobutylaminocarbonyl-dU (iBudU). Since the incorporation of these modified nucleotides into SELEX experiments, our success overall success rate (pool $K_d$ < ~30nM) is ~84% (1204/1428) for high quality SOMAmers to a wide range of human proteins. The 813 human proteins measured by the current array are shown in Table 2 (at the end of this document).

Table 1. SELEX library affinities ($K_d$, M) with unmodified and modified nucleotides

| Target Protein | dT | BndU | iBudU | TrpdU |
|---|---|---|---|---|
| 4-1BB[a] | failed | $6 \times 10^{-9}$ | failed | $4 \times 10^{-9}$ |
| B7[a] | failed | $1 \times 10^{-8}$ | failed | $7 \times 10^{-9}$ |
| B7-2[a] | failed | failed | failed | $6 \times 10^{-9}$ |
| CTLA-4[a] | failed | failed | failed | $1 \times 10^{-9}$ |
| sE-Selectin[a] | failed | failed | failed | $2 \times 10^{-9}$ |
| Fractalkine/CX3CL-1 | failed | failed | failed | $5 \times 10^{-11}$ |
| GA733-1 protein[a] | $9 \times 10^{-9}$ | $3 \times 10^{-9}$ | $5 \times 10^{-9}$ | $5 \times 10^{-10}$ |
| gp130, soluble[a] | failed | $6 \times 10^{-9}$ | $2 \times 10^{-8}$ | $1 \times 10^{-9}$ |
| HMG-1 | failed | failed | $2 \times 10^{-8}$ | $5 \times 10^{-9}$ |
| IR | failed | $2 \times 10^{-9}$ | $1 \times 10^{-8}$ | $2 \times 10^{-10}$ |
| Osteoprotegerin[a] | $4 \times 10^{-8}$ | $5 \times 10^{-9}$ | $9 \times 10^{-9}$ | $2 \times 10^{-10}$ |
| PAI-1 | failed | $4 \times 10^{-10}$ | $9 \times 10^{-10}$ | $2 \times 10^{-10}$ |
| P-Cadherin[a] | failed | $4 \times 10^{-9}$ | $5 \times 10^{-9}$ | $3 \times 10^{-9}$ |
| sLeptin R[a] | failed | $2 \times 10^{-9}$ | failed | $5 \times 10^{-10}$ |

[a]The protein used was expressed as a fusion to the Fc of human $IgG_1$. No detectable binding of the active library to an alternate Fc fusion protein was observed.

Nature Precedings : hdl:10101/npre.2010.4538.1 : Posted 14 Jun 2010

### SOMAmer Specificity

We assessed the specificity of select SOMAmers for the targets they were selected against in an affinity binding assay that mimics our multiplexed proteomics assay. The experimental method is outlined in Figure 1 and detailed below in the Materials and Methods section. This experiment mimics Catch 1 and Catch 2 in the proteomics assay and then uses a third step to capture the bound SOMAmer-protein complex with an oligo that is complementary to a portion of the SOMAmer and acts as an affinity tag. This “Catch 3” step is analogous to the DNA microarray hybridization step in the proteomics assay. The captured complexes are then disrupted and the proteins are eluted and analyzed by denaturing poly-acrylamide gel electrophoresis (PAGE), as shown in Figure 3b (main document). Not all complexes are able to be captured in the

“Catch 3” step, and therefore analyzed by PAGE, since the 3’ end of the SOMAmer is sometimes involved in its structure or interaction with the target. Additional pull-down examples for the subset of the CKD-related targets whose complexes can be captured on “Catch 3” beads are shown in Figure 2.

To further assess the specificity of selected (>20) SOMAmers for their target proteins, we excised the resulting PAGE gel samples (entire lanes) from the plasma affinity binding experiment described above and analyzed them by mass spectrometry (MS). In all cases, the results confirmed that the gel band contained the target protein. This was evidenced by the identification of peptides that map to the target protein using their specific fragmentation patterns. Peptides that mapped to other proteins were also identified, although the number of spectra for these proteins was typically much lower than for the specific target. Such contaminants are a relatively small fraction because the PAGE gels show sharp uniform bands with very little background, which suggests that the majority of the material is the target protein.

Nature Precedings : hdl:10101/npre.2010.4538.1 : Posted 14 Jun 2010

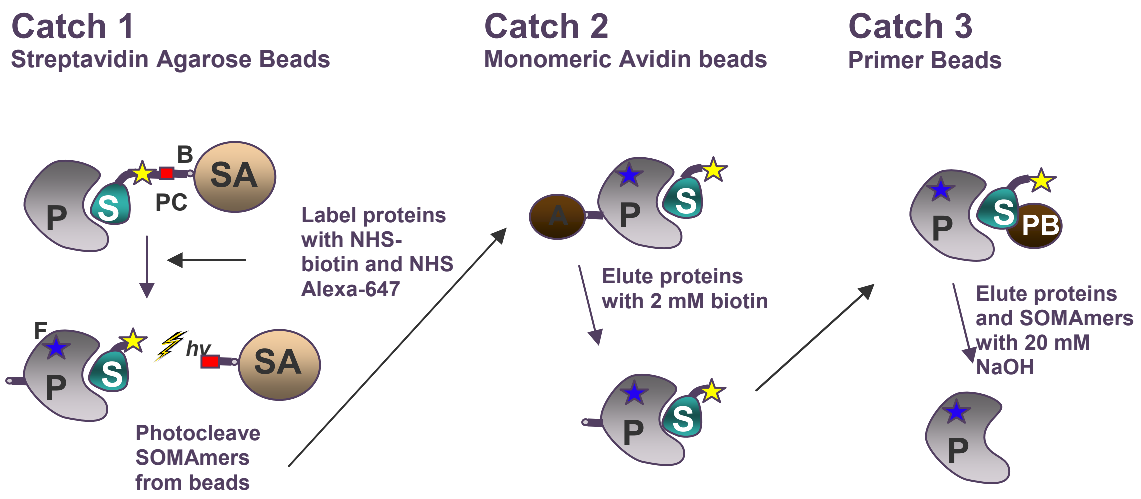

**Figure 1.** Affinity binding pulldown assay. SOMAmers are mixed with the target sample (purified protein or plasma) and incubated to bind to equilibrium. In **Catch 1** bound SOMAmer(S)-protein(P) complexes are captured onto streptavidin beads (SA) and the proteins are tagged with biotin (B) (NHS- biotin) and fluorescent label (F) (NHS Alexa 647). Unbound proteins are washed away. Bound complexes are released from the beads by cleaving the photo-cleavable linker (PC) with ultraviolet light. In **Catch 2** SOMAmer-protein complexes are captured onto monomeric avidin beads (A), washed, and eluted from the beads with 2 mM biotin. At this stage, SOMAmer-protein complexes are subjected to a kinetic challenge analogous to that used in the proteomics assay. Specific complexes survive the challenge and non-specific complexes dissociate. In the final step, **Catch 3**, bound complexes are captured onto primer beads (PB) by DNA primer that is complementary to a portion of the SOMAmer and any remaining unbound protein resulting from the kinetic challenge is washed away. Finally, the captured complexes are dissociated with 20 mM NaOH and the target protein is eluted for analysis by PAGE.

Nature Precedings : hdl:10101/npre.2010.4538.1 : Posted 14 Jun 2010

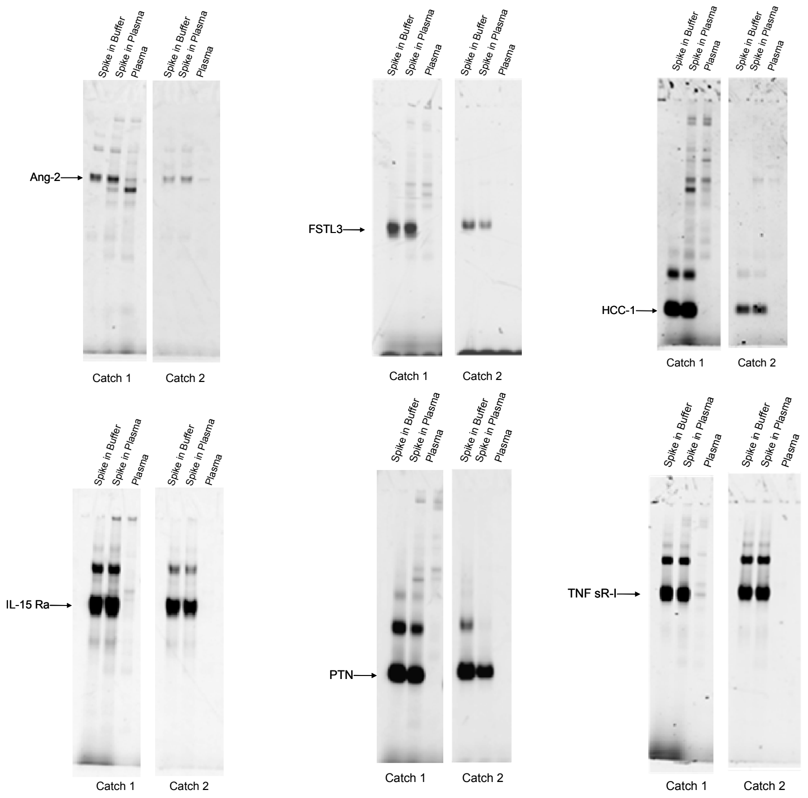

**Figure 2**. Affinity assay pulldowns for SOMAmers selected against six CKD-related proteins show specificity of SOMAmers for their target proteins. For each example the gel shows the results for purified target protein spiked into buffer (lane 1), purified target protein spiked into 10% plasma (lane 2), and 10% plasma (lane 3). The first set of three lanes demonstrates all of the proteins eluted from catch-1 beads. The second set of lanes shows the aptamer-bound proteins eluted from catch-2 beads.

Nature Precedings : hdl:10101/npre.2010.4538.1 : Posted 14 Jun 2010

### Assay Reproducibility

To assess the reproducibility of our proteomic measurements, we measured multiple replicate samples of serum and plasma in a single assay run. Each run spans an entire 96-well microtiter plate. Multiple replicate runs are performed and CVs are calculated for each analyte as a measure of reproducibility.

Three independent automated assay runs (A, B, and C) were initiated and completed on two different days. Each run was comprised of samples from eighteen different individuals run in five replicates along with six no-protein buffer controls to assess assay background. See Materials and Methods below for detailed methods.

**Reproducibility Measuring Plasma.** The CV for each SOMAmer was computed for each sample by averaging over the replicates and then averaging these CVs over all the samples. Both intra- and inter-plate CVs were computed for each dilution mix and are displayed in Figure 3 and summarized in Table 3 below. The median intra- and inter-plate CVs are 3.8% and 4.3% for SOMAmers in the 10% mix, 4.4% and 5.5% for SOMAmers in the 1% mix, and 5.6% and 6.8% for SOMAmers in the 0.03% mix.

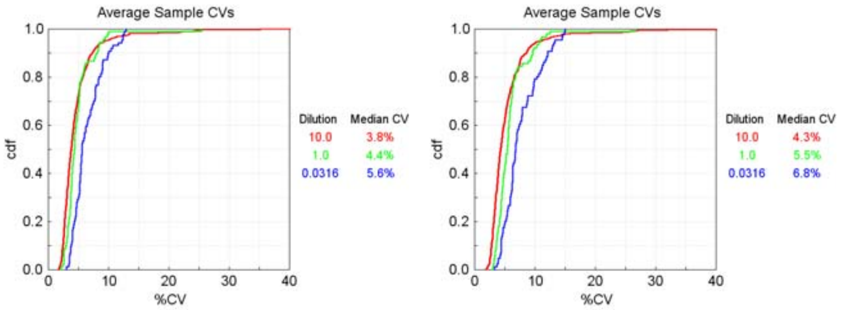

**Figure 3.** Distributions for intra-run and inter-run CVs for plasma. The cdfs for the intra-run CVs are on the left and the inter-run CVs are on the right for the three dilutions mixes, 10% (red), 1% (green), and 0.03% (blue).

| Pr[X≤x] = α | | | |
|---|---|---|---|
| Intra-Run | x | | |
| α | 10.00% | 1.00% | 0.03% |
| 0.05 | 2.28 | 2.66 | 3.57 |
| 0.25 | 2.94 | 3.70 | 4.67 |
| 0.50 | 3.85 | 4.40 | 5.63 |
| 0.75 | 5.24 | 5.27 | 7.87 |
| 0.95 | 9.31 | 9.15 | 12.22 |

| Pr[X≤x] = α | | | |
|---|---|---|---|
| Inter-Run | x | | |
| α | 10.00% | 1.00% | 0.03% |
| 0.05 | 2.63 | 3.34 | 3.99 |
| 0.25 | 3.41 | 4.40 | 5.59 |
| 0.50 | 4.34 | 5.48 | 6.84 |
| 0.75 | 6.02 | 6.38 | 9.76 |
| 0.95 | 10.72 | 10.96 | 13.33 |

**Table 3.** Summary statistics for intra- and inter-run CV*s* broken out by plasma dilution (10%, 1%, and 0.03%).

**Reproducibility Measuring Serum.** The CV for each SOMAmer was computed for each sample by averaging over the replicates and then averaging these CVs over all the samples. Both intra- and inter-plate CVs were computed for each dilution mix and are displayed in Figure 4 and summarized in Table 4 below. The median intra- and inter-plate CVs are 4.3% and 5.0% for SOMAmers in the 10% mix, 4.2% and 4.9% for SOMAmers in the 1% mix, and 5.3% and 6.4% for SOMAmers in the 0.03% mix

Nature Precedings : hdl:10101/npre.2010.4538.1 : Posted 14 Jun 2010

Nature Precedings : hdl:10101/npre.2010.4538.1 : Posted 14 Jun 2010

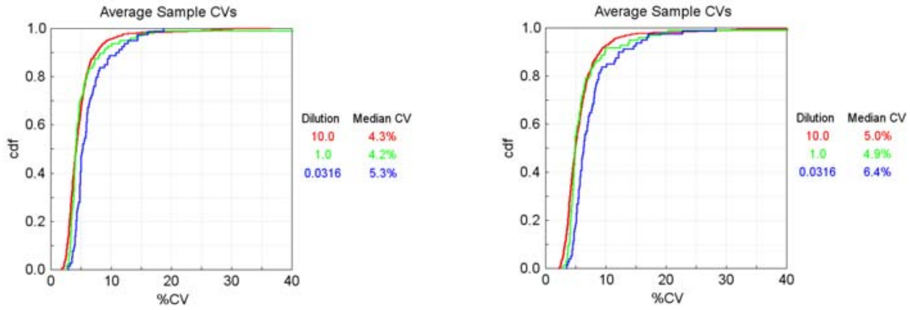

**Figure 4.** The distributions for intra-run and inter-run CVs for serum. The cdfs for the intra-run CVs are on the left and the inter-run CVs are on the right for the three dilutions mixes, 10% (red), 1% (blue), and 0.03% (green). The inter-run CVs are only slightly higher than the intra-run CVs.

| Pr[X≤x] = α | | | |
|---|---|---|---|
| **Intra-Run** | **x** | | |
| α | **10.00%** | **1.00%** | **0.03%** |
| 0.05 | 2.55 | 3.07 | 3.60 |
| 0.25 | 3.34 | 3.60 | 4.43 |
| 0.50 | 4.26 | 4.17 | 5.30 |
| 0.75 | 5.57 | 5.50 | 7.10 |
| 0.95 | 9.43 | 11.42 | 12.82 |

| Pr[X≤x] = α | | | |
|---|---|---|---|
| **Inter-Run** | **x** | | |
| α | **10.00%** | **1.00%** | **0.03%** |
| 0.05 | 2.96 | 3.67 | 4.27 |
| 0.25 | 3.94 | 4.35 | 5.24 |
| 0.50 | 4.97 | 4.93 | 6.39 |
| 0.75 | 6.62 | 6.45 | 8.39 |
| 0.95 | 11.36 | 13.97 | 15.94 |

**Table 4.** Summary statistics for intra- and inter-run CVs for serum.

### Limits and Ranges of Quantification

In order to determine the quantitative performance of our proteomics platform, we generated precision profiles for 356 analytes. The overall results are presented in Table 5 at the end of this document. A precision profile, which shows the variation in %CV for calculated concentration as a function of analyte concentration, provides an analytic measurement of assay performance and establishes the limits of quantification (LOQ) – both the upper and lower limits of quantification (ULOQ and LLOQ) – which define the dynamic range for analyte measurements. We have focused on optimizing and assessing LLOQs in the assay. Therefore, in some cases ULOQ measurements did not plateau in the measured range and represent a minimum estimate of ULOQ and, therefore, a minimum estimate of the range of quantification (ROQ).

**Buffer Experiments.** The LOQ experiments measured six-point standard curves spanning six logs in concentration, from 10 nM to 10 fM, for a series of analytes in a multiplexed fashion. A set of proteins was combined at the highest concentration and serially diluted 1:10 to create a set of standards that spanned six logs in concentration. Each analyte concentration was measured eight times to determine the assay error at each

Nature Precedings : hdl:10101/npre.2010.4538.1 : Posted 14 Jun 2010

concentration. Approximately 90 proteins were combined and mixed with their respective SOMAmer dilution mixes for equilibration. After Catch 1 these were combined for Catch 2 and hybridization as per standard assay protocol (see Supplementary Methods section below for assay details). Two such runs fit on each dilution plate, yielding a total of 356 analyte measurements for precision profiles.

**Data Analysis.** The precision profile was constructed from a series of standard curves. The assay variance as a function of concentration was computed from these data. Typically, the relative error (CV), the standard deviation ($\sigma$) of the calculated concentration divided by the concentration, is determined for computing LOQs. As analyte concentration approaches zero, the assay CV diverges. Similarly, for large analyte concentrations near the assay plateau, small changes in assay signal can give rise to large changes in calculated concentration, leading again to a divergence in CVs. In between these two divergences in CVs lies a concentration range for which the assay measurements have CVs of a desired limit or less.

We set this limit at 20% CV and determined the upper and lower LOQs as those high and low concentrations equal to 20% CV. Standard curves were computed by averaging the relative fluorescent units (RFUs) for eight replicate measurements at each concentration. A standard four parameter Hill model (Eq. 1) in log transformed RFU was used to fit the dose-response curves, where x denotes an analyte concentration.

$$\log RFU = (\log RFU_{plateau} - \log RFU_{baseline})\frac{x^{\alpha}}{x^{\alpha} + K^{\alpha}} + \log RFU_{baseline} \qquad (1)$$

A typical dose-response curve from the data set is displayed in Figure 5.

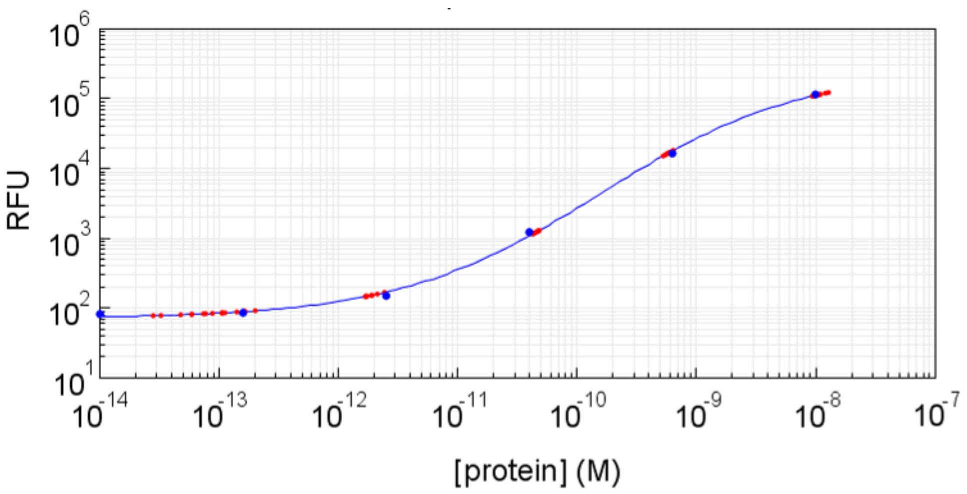

**Figure 5.** a2-Antiplasmin. Dose-response curve using a four-parameter fit. The average RFU at each concentration is denoted by the blue markers and the eight individual measurements used to compute the average are denoted by the red markers plotted on the curve fit (solid blue line).

Nature Precedings : hdl:10101/npre.2010.4538.1 : Posted 14 Jun 2010

Two distinct approaches were used to compute precision profiles from these data. The first approach modeled the standard deviation for calculated concentrations $\sigma_x$, obtained by averaging the eight replicates at each concentration, with a quadratic function from which the precision profile was directly obtained. Figure 6 illustrates this approach for the analyte displayed in Figure 5. The second approach is to model the standard deviation of the assay response $\sigma_{logRFU}$ with a quadratic function and then use the dose-response function to compute the variance in concentration from the response variance. This is not easily accomplished for the dose-response function used here but linearizing the function at a concentration $x$ leads to the following simplification (Eq. 2 and 3).

$$\sigma_x = \frac{\sigma_{\log RFU}}{\left(\dfrac{\partial \log RFU}{\partial x}\right)} \tag{2}$$

$$\left(\frac{\partial \log RFU}{\partial x}\right) = (\log RFU_{plateau} - \log RFU_{baseline})\frac{\alpha x^{\alpha-1} K^{\alpha}}{\left(x^{\alpha} + K^{\alpha}\right)^2} \tag{3}$$

Typically, the assay CV in response units ($\sigma_{logRFU}$ /$logRFU$) is fairly constant so using a quadratic function to model $\sigma_{RFU}$ as a function of concentration should suffice. Figure 7 illustrates this for the data in Figure 5.

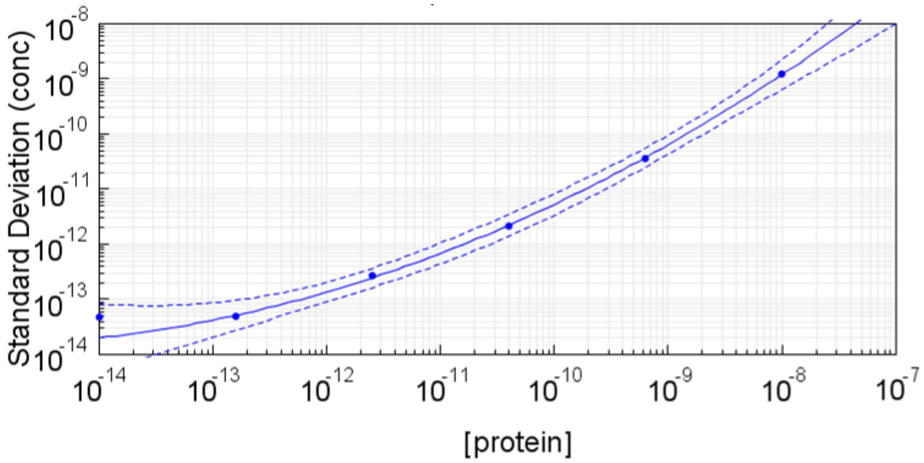

**Figure 6.** Standard deviation of calculated concentration. The standard deviation $\sigma_x$ for computed concentration is denoted by a blue marker. The quadratic fit is displayed as a solid blue line and the 95% confidence bands for the fit are displayed as dashed lines.

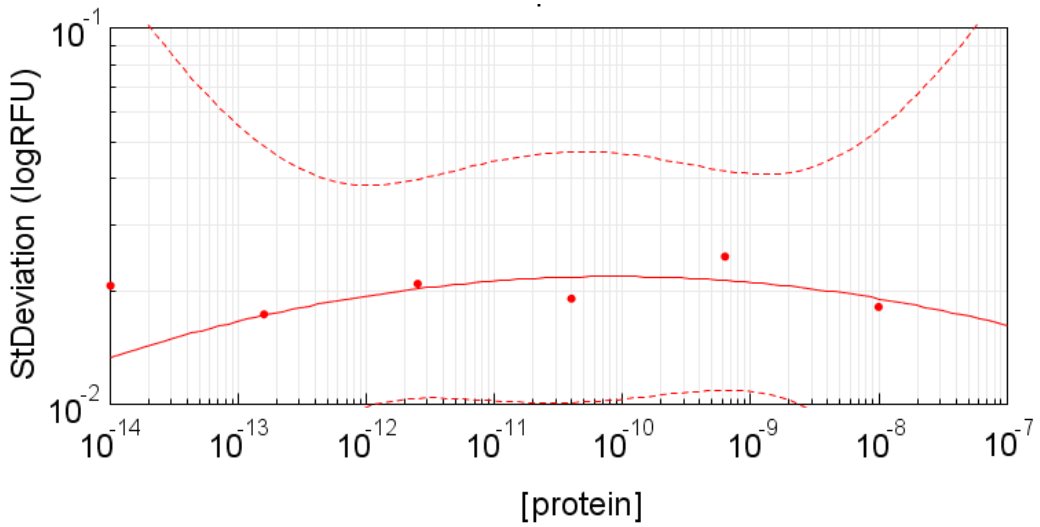

**Figure 7.** Standard deviation of assay response. The standard deviations for logRFU $\sigma_{logRFU}$ are denoted by the red markers. The quadratic fit is displayed as a solid red line and the 95% confidence bands for the fit are displayed as dashed lines.

We produced the full precision profile for each SOMAmer tested using both numerical approaches outlined above. The results for the analyte shown in Figure S5 are presented below in Figure S8 for (a) modeling $\sigma_x$ directly (blue line) and (b) modeling $\sigma_{logRFU}$ from which $\sigma_x$ is computed (red line). Both methods give essentially the same result in this case for LLOQ and ULOQ. This particular analyte shows a remarkable

Nature Precedings : hdl:10101/npre.2010.4538.1 : Posted 14 Jun 2010

five-log quantification range at a 20% CV cutoff with an LLOQ of 0.4-0.6 pM and a ULOQ of 40-50 nM. In general there is good agreement between the two different methods for computing precision profiles, and the assay response $\sigma_{logRFU}$ method was used to calculate the values shown in Table 5.

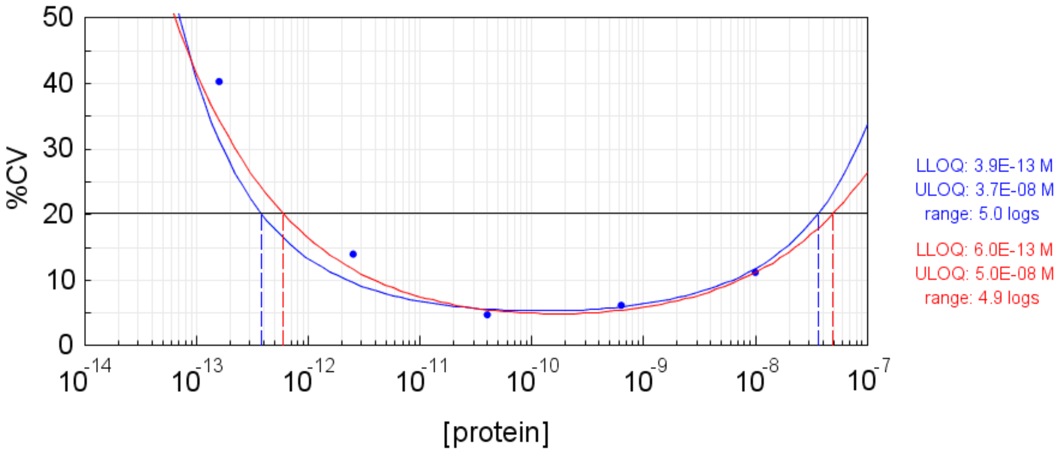

**Figure 8.** Precision profiles for assay response computed from two different methods. The precision profile computed by modeling $\sigma_x$ is displayed in blue and the profile for modeling $\sigma_{logRFU}$ is displayed in red. The methods are consistent.

**Plasma versus Buffer LOQs.** The LLOQs, ULOQs, and range of quantification were computed for twenty-eight analytes as spikes into both plasma and buffer. The results are summarized in Table 6 at the end of this document and in Figures S9-S11. The LOQs determined from spiking analytes into plasma and buffer agree well. Both the LLOQs and the ULOQs are consistent between the two fluids; see Figures S9 and S10 below. The ranges of quantification are also in good agreement, see Figure 11, and are centered at 3 logs of dynamic range. In general, these results illustrate that the quantitative behavior of our multiplexed assay, as characterized by precision profiles, is not affected by fluid matrix effects, allowing us to use protein spikes into buffer to assess the quantitative behavior of our multiplexed assay.

Nature Precedings : hdl:10101/npre.2010.4538.1 : Posted 14 Jun 2010

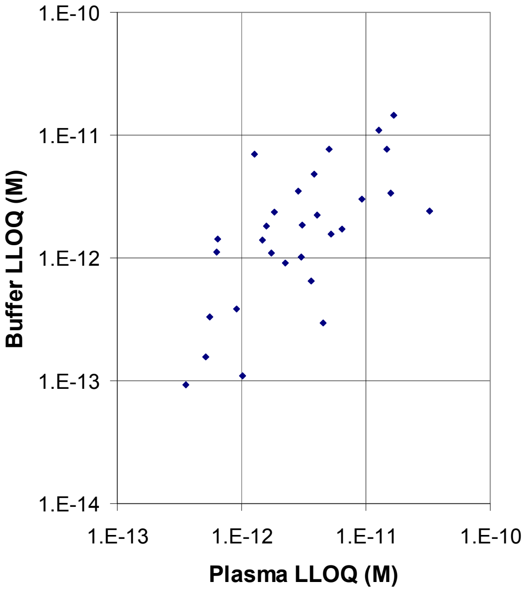

**Figure 9.** Scatter plot of LLOQs Determined in Plasma and Buffer for 28 analytes. These data were computed by modeling $\sigma_{logRFU}$ as described above.

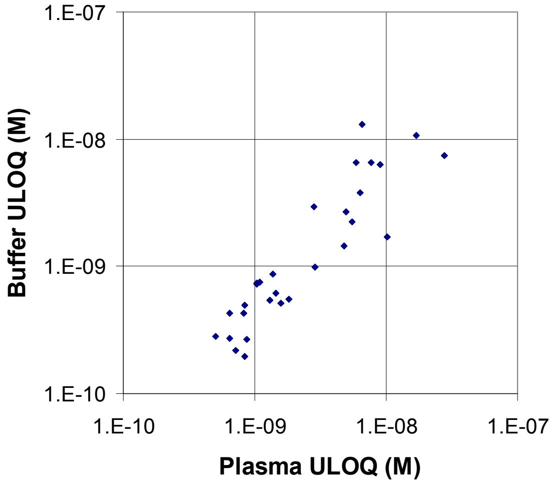

**Figure 10.** Scatter plot of ULOQs Determined in Plasma and Buffer for 28 analytes. These data were computed by modeling $\sigma_{logRFU}$ as described above.

Nature Precedings : hdl:10101/npre.2010.4538.1 : Posted 14 Jun 2010

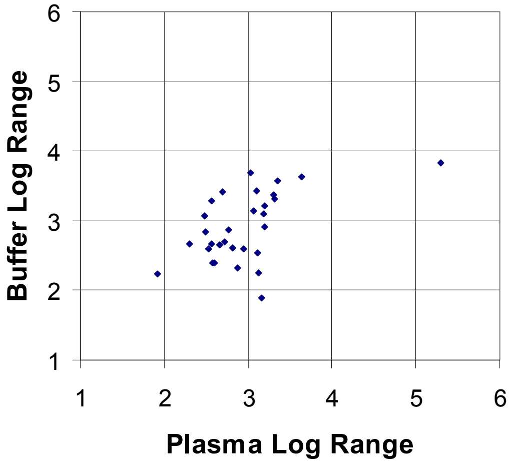

**Figure 11.** Scatter plot of Ranges of Quantification Determined in Plasma and Buffer for 28 analytes. These data were computed by modeling $\sigma_{logRFU}$ as described above.

**LLOQ.** The LLOQs were computed from the precision profiles of the analytes measured in buffer. Greater than 95% of the analytes examined produced precision profiles with quantification ranges below a 20% CV cutoff. The distribution of determined LLOQs is shown in Figure 12 and summarized in Table 7. The median LLOQ is 0.9 pM and the inter-quartile range is 0.3 pM – 3.9 pM. Over half of the analytes examined have LLOQs that are < 1.0 pM. Although some analytes appear quantitative below 10 fM, these need to be verified with lower measurements.

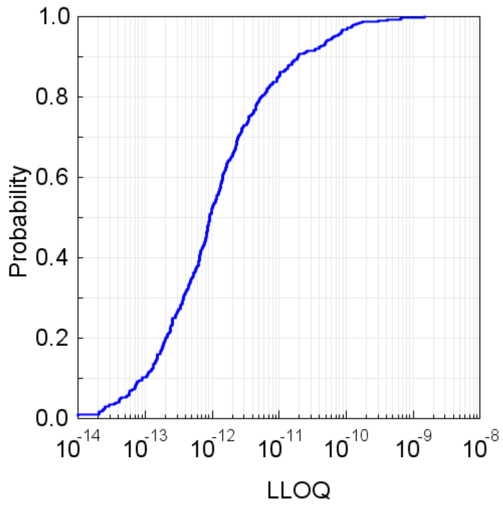

**Figure 12.** Distribution of the LLOQs for 356 analytes. The cumulative distribution function for LLOQ is displayed in the plot.

Nature Precedings : hdl:10101/npre.2010.4538.1 : Posted 14 Jun 2010

| Pr[X≤x] = α | |
|---|---|
| | LLOQ |
| α | x |
| 0.05 | 4.4E-14 |
| 0.25 | 2.9E-13 |
| 0.50 | 9.4E-13 |
| 0.75 | 3.9E-12 |
| 0.95 | 7.9E-11 |

**Table 7.** Summary of the LLOQs for 356 analytes.

**ULOQ.** The distribution of determined ULOQs computed from the buffer precision profiles is given in Figure S13 and summarized in Table 8. The median ULOQ is 1.5 nM and the inter-quartile range is 0.7 nM – 4.5 nM. Although some analytes in the present analysis appear to be quantitative above 10 nM, these results need to be verified by making measurements higher than those in this study. For example, albumin's dose-response curve is still increasing at 10 nM and so an accurate determination of the ULOQ is not possible from this data.

Nature Precedings : hdl:10101/npre.2010.4538.1 : Posted 14 Jun 2010

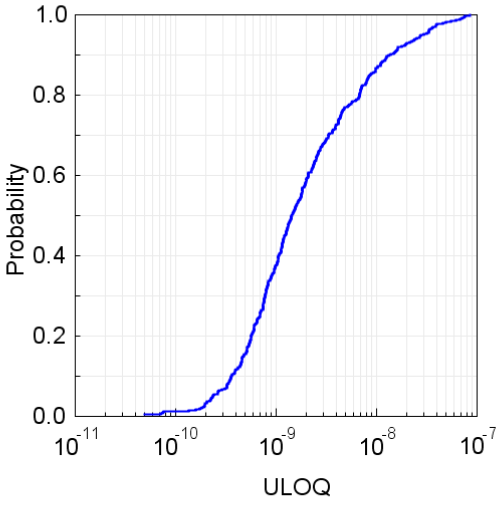

**Figure S13.** Distribution of the ULOQs for 356 analytes. The cumulative distribution function for ULOQ is displayed in the plot.

Nature Precedings : hdl:10101/npre.2010.4538.1 : Posted 14 Jun 2010

| Pr[X≤x] = α | |
|---|---|
| | ULOQ |
| α | x |
| 0.05 | 2.4E-10 |
| 0.25 | 7.0E-10 |
| 0.50 | 1.5E-09 |
| 0.75 | 4.5E-09 |
| 0.95 | 3.0E-08 |

**Table 8.** Summary of the ULOQs for 356 analytes. A summary of the ULOQ data is presented in the table.

**Range of Quantification.** The total quantitative range of quantification (ROQ) can be defined as the difference between log ULOQ and log LLOQ. Based on the average LLOQ and ULOQ, the expected median quantification range is ~ 3 logs. Figure S14 shows the distribution of the quantification range which is summarized in Table 9. The median range is 3.2 logs, consistent with the LOQs discussed above, and the center 50% have ranges from 2.8-3.7 logs. For those analytes that exceed four logs of quantification additional measurements are required for verification.

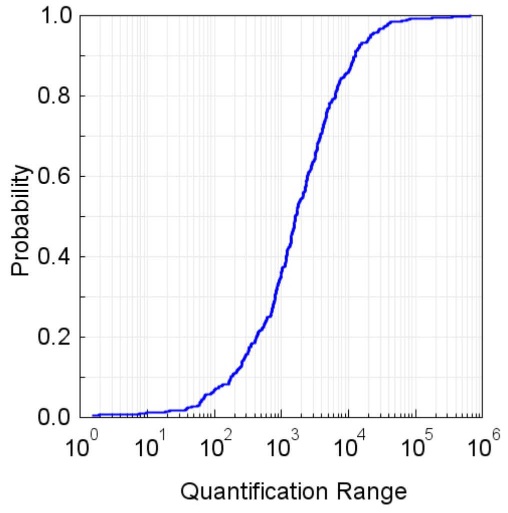

**Figure S14.** Distribution of the log quantification range for 356 analytes. The cumulative distribution function for the log of the quantification range is displayed in the plot.

| Pr[X≤x] = α | |
|---|---|
| | log Range |
| α | x |
| 0.05 | 1.9 |
| 0.25 | 2.8 |
| 0.50 | 3.2 |
| 0.75 | 3.7 |
| 0.95 | 4.3 |

**Table 9.** Summary of the log quantification range for 356 analytes. A summary of the quantification range data is presented in the table.

A summary of the calculated LLOQ, ULOQ quantitative range for each analyte is provided in Table 5 at the end of this document. The analytes have been grouped by their dilution mixes and sorted with respect to LLOQ. In addition to the quantification metrics, the four parameters used in the dose-response curves are provided as well. An entry of $10^{-6}$ M for LLOQ or ULOQ indicates that a limit has not been established from the data. There are sixteen analytes for which both LLOQ and ULOQ are not determined and so the range is denoted as zero or the standard curves were not properly fit and so have no parameters listed. Both these cases occur at the end of the lists.

### Chronic Kidney Disease Biomarkers

Table 10 lists sixty potential CKD biomarkers identified in this study comparing early- to late-stage disease with the Mann-Whitney U-test with an alpha of 4.3E-04 for the q-value (false discovery rate corrected p-value).

Nature Precedings : hdl:10101/npre.2010.4538.1 : Posted 14 Jun 2010

### Table 10. Potential CKD Biomarkers

| Target | p-value | q-value | Mol. Mass (kDa) |
|---|---|---|---|
| β2-Microglobulin | 1.19E-09 | 7.98E-08 | 11.7 |
| FSTL3 | 1.19E-09 | 7.98E-08 | 25.0 |
| Pleiotrophin | 1.19E-09 | 7.98E-08 | 15.3 |
| TNF sR-I | 1.19E-09 | 7.98E-08 | 48.3 |
| Factor D | 4.77E-09 | 2.13E-07 | 24.4 |
| IL-15 Rα | 4.77E-09 | 2.13E-07 | 25.0 |
| MMP-7 | 8.35E-09 | 3.19E-07 | 19.1 |
| Angiopoietin-2 | 1.43E-08 | 3.48E-07 | 55.1 |
| Cystatin C | 1.43E-08 | 3.48E-07 | 13.3 |
| HCC-1 | 1.43E-08 | 3.48E-07 | 8.7 |
| URB | 1.43E-08 | 3.48E-07 | 105.7 |
| Lysozyme | 3.58E-08 | 7.36E-07 | 14.7 |
| ROR1 | 3.58E-08 | 7.36E-07 | 101.2 |
| Chordin-Like 1 | 5.37E-08 | 1.03E-06 | 48.8 |
| Endostatin | 7.99E-08 | 1.43E-06 | 20.1 |
| Ephrin-A5 | 1.66E-07 | 2.61E-06 | 23.9 |
| Matrilin-2 | 1.66E-07 | 2.61E-06 | 104.4 |
| IGFBP-6 | 4.42E-07 | 6.56E-06 | 22.6 |
| Granzyme B | 5.98E-07 | 8.41E-06 | 25.5 |
| DAN | 1.06E-06 | 1.42E-05 | 17.7 |
| β-NGF | 1.39E-06 | 1.69E-05 | 13.5 |
| Nectin-like protein 2 | 1.39E-06 | 1.69E-05 | 48.6 |
| CXCL16, soluble | 1.82E-06 | 2.11E-05 | 24.2 |
| IGFBP-2 | 2.35E-06 | 2.51E-05 | 31.3 |
| SLPI | 2.35E-06 | 2.51E-05 | 11.7 |
| TGF-β R III | 3.84E-06 | 3.95E-05 | 91.3 |
| CNTFR α | 4.87E-06 | 4.66E-05 | 35.8 |
| Lymphotoxin α1/β2 | 4.87E-06 | 4.66E-05 | 69.4 |
| CD48 | 7.51E-06 | 6.64E-05 | 22.3 |
| Lymphotoxin β R | 7.69E-06 | 6.64E-05 | 43.7 |
| Troponin I | 7.69E-06 | 6.64E-05 | 23.9 |
| ESAM | 1.19E-05 | 9.63E-05 | 38.1 |
| NovH | 1.19E-05 | 9.63E-05 | 35.7 |
| HCC-4 | 1.47E-05 | 1.15E-04 | 11.2 |
| CD30 Ligand | 1.80E-05 | 1.30E-04 | 26.0 |
| MIA | 1.80E-05 | 1.30E-04 | 12.1 |
| BCMA | 2.20E-05 | 1.44E-04 | 20.1 |
| Insulysin | 2.20E-05 | 1.44E-04 | 117.9 |
| Thrombospondin-1 | 2.20E-05 | 1.44E-04 | 128.0 |
| Trypsin | 2.20E-05 | 1.44E-04 | 24.1 |
| Cystatin M | 2.69E-05 | 1.71E-04 | 13.6 |
| Bcl-2 | 3.26E-05 | 1.89E-04 | 26.3 |
| Kallikrein 6 | 3.26E-05 | 1.89E-04 | 24.5 |
| LSAMP | 3.26E-05 | 1.89E-04 | 31.8 |
| NKG2D | 3.26E-05 | 1.89E-04 | 25.3 |
| Desmoglein-1 | 3.94E-05 | 2.07E-04 | 107.7 |
| Dtk | 3.94E-05 | 2.07E-04 | 92.8 |
| EphA1 | 3.94E-05 | 2.07E-04 | 106.0 |
| MIP-3β | 3.94E-05 | 2.07E-04 | 8.8 |
| Myoglobin | 3.94E-05 | 2.07E-04 | 17.0 |
| Biglycan | 4.74E-05 | 2.31E-04 | 37.2 |
| CD30 | 4.74E-05 | 2.31E-04 | 61.9 |
| IL-17D | 4.74E-05 | 2.31E-04 | 20.3 |
| MIP-5 | 4.74E-05 | 2.31E-04 | 10.2 |
| Fractalkine/CX3CL-1 | 5.69E-05 | 2.72E-04 | 39.6 |
| IL-18 BPa | 6.81E-05 | 3.19E-04 | 17.6 |
| GA733-1 protein | 8.11E-05 | 3.68E-04 | 33.1 |
| Galectin-3 | 8.11E-05 | 3.68E-04 | 26.1 |
| bFGF-R | 9.64E-05 | 4.23E-04 | 89.4 |
| PECAM-1 | 9.64E-05 | 4.23E-04 | 79.6 |

Nature Precedings : hdl:10101/npre.2010.4538.1 : Posted 14 Jun 2010

Nature Precedings : hdl:10101/npre.2010.4538.1 : Posted 14 Jun 2010

## SUPPLEMENTARY METHODS

### SOMAmer development and SELEX

Selection methods have been developed for use with poly-His-tagged, biotinylated, and non-tagged proteins. Many variations on these protocol have been used to select the >800 SOMAmers for the proteomic platform, such as alternating selection conditions to increase stringency for slow off-rate SOMAmers or performing the equilibrium steps in solution rather than with targets pre-immobilized. The following protocol is representative and was used for the selection described in the main text of this paper (results shown in Table 1). Selection methods are further detailed in our patents and published patent applications[1,2].

**Preparation of Candidate Mixtures.** Candidate mixtures were prepared with dATP, dGTP, 5-methyl-dCTP (MedCTP) and either dTTP or one of three dUTP analogs: 5-benzylaminocarbonyl-dU (BndU), 5-tryptaminocarbonyl-dU = TrpdU, and 5-isobutylaminocarbonyl-dU (iBudU) (Figure 1 of paper). Candidate mixtures were prepared by polymerase extension of a primer annealed to a biotinylated template. For each candidate mixture composition, 4.8 nmol forward PCR primer and 4 nmol template were combined in 100 μL 1X *Thermococcus kodakaraensis* (KOD) XL DNA Polymerase Buffer (EMD), heated to 95°C for 8 minutes, and cooled on ice. Each 100 μL primer:template mixture was added to a 400 μL extension reaction containing 1X KOD DNA Polymerase Buffer, 0.125 U/μL KOD DNA Polymerase, and 0.5 mM each dATP, MedCTP, dGTP, and dTTP or dUTP analog, and incubated 70°C, 30 minutes. Double-stranded product was captured via the template strand biotins by adding 1 mL streptavidin-coated magnetic beads (MagnaBind Streptavidin, Pierce, 5 mg/mL in 1M NaCl + 0.05% TWEEN-20) and incubating 25°C for 10 minutes with mixing. Beads were washed three times with 0.75 mL SB1T Buffer (40 mM HEPES, pH 7.5, 125 mM NaCl, 5 mM KCl, 1 mM $MgCl_2$, 1 mM $CaCl_2$, 0.05% TWEEN-20). The SOMAmer strand was eluted from the beads with 1.2 mL 20 mM NaOH, neutralized with 0.3 mL 80 mM HCl, and buffered with 15 μL 1 M HEPES, pH 7.5. Candidate mixtures were concentrated with a Centricon-30 to approximately 0.2 mL, and quantified by UV absorbance spectroscopy.

**Immobilization of Target Proteins.** Target proteins were purchased with $(His)_6$ tags (R&D Systems, Millipore) and immobilized on $Co^{+2}$-NTA paramagnetic beads (TALON, Dynal). Target proteins were diluted to 0.2 mg/mL in 0.5 mL B/W Buffer (50 mM Na-phosphate, pH 8.0, 300 mM NaCl, 0.01% TWEEN-20), and added to 0.5 mL TALON beads (pre-washed three times with B/W Buffer and resuspended to 10 mg/mL in B/W Buffer). The mixture was rotated for 30 minutes at 25°C and stored at 4°C until use. TALON beads coated with $(His)_6$ peptide were also prepared and stored as above. Prior to use, beads were washed 3 times with B/W Buffer, once with SB1T, and resuspended in SB1T.

**SOMAmer Selection.** Affinity selections were performed separately with each candidate mixture, comparing binding between target protein beads (signal) and $(His)_6$ beads (background). For each sample, a 0.5 μM candidate DNA mixture was prepared in 40 μL SB1T. 1 μL of 1 mM competitor oligo was added to the DNA, along with 10 μL of a protein competitor mixture (0.1% HSA, 10 μM casein, and 10 μM prothrombin in SB1T).

Binding reactions were performed by adding 50 μL target protein-coated beads or $(His)_6$-coated beads (5 mg/mL in SB1T) to the DNA mixture and incubating 37°C for 15 minutes with mixing. The DNA solution was removed and the beads were washed 5 times at 37°C with SB1T containing 0.1 mg/mL herring sperm DNA (Sigma). Unless indicated, all washes were performed by resuspending the beads in 100 μL wash solution, mixing for 30 seconds, separating the beads with a magnet, and removing the wash solution. Bound SOMAmers were eluted from the beads by adding 100 μL SB1T + 2 M Guanidine-HCl and incubating 37°C, 5 minutes with mixing. The SOMAmer eluate was transferred to a new tube after magnetic separation. After the first two selection rounds, the final two of five target beads washes were done for 5 minutes instead of 30 seconds.

Primer beads were prepared by immobilizing biotinylated reverse PCR primer to streptavidin-coated paramagnetic beads (MyOne-SA, Dynal). 5 mL MyOne-SA beads (10 mg/mL) were washed once with NaClT (5 M NaCl, 0.01% TWEEN-20), and

Nature Precedings : hdl:10101/npre.2010.4538.1 : Posted 14 Jun 2010

resuspended in 5 mL biotinylated reverse PCR primer (5 μM in NaClT). The sample was incubated 25°C, 15 minutes, washed twice with 5 mL NaClT, resuspended in 12.5 mL NaClT (4 mg/mL), and stored at 4°C.

25 μL primer beads (4 mg/mL in NaClT) were added to the 100 μL SOMAmer solution in Guanidine Buffer and incubated 50°C, 15 minutes with mixing. The SOMAmer solution was removed, and the beads were washed 5 times with SB1T. SOMAmer was eluted from the beads by adding 85 μL 20 mM NaOH, and incubating 37°C, 1 minute with mixing. 80 μL SOMAmer eluate was transferred to a new tube after magnetic separation, neutralized with 20 μL 80 mM HCl, and buffered with 1 μL 0.5M Tris-HCl, pH 7.5.

Nature Precedings : hdl:10101/npre.2010.4538.1 : Posted 14 Jun 2010

**SOMAmer Amplification and Purification.** Selected SOMAmer DNA was amplified and quantified by QPCR. 48 μL DNA was added to 12 μL QPCR Mix (5X KOD DNA Polymerase Buffer, 25 mM $MgCl_2$, 10 μM forward PCR primer, 10 μM biotinylated reverse PCR primer, 5X SYBR Green I, 0.125 U/μL KOD DNA Polymerase, and 1 mM each dATP, dCTP, dGTP, and dTTP) and thermal cycled in an ABI5700 QPCR instrument with the following protocol: 1 cycle of 99.9°C, 15 seconds, 55°C, 10 seconds, 70°C, 30 minutes; 30 cycles of 99.9°C, 15 seconds, 72°C, 1 minute. Quantification was done with the instrument software and the number of copies of DNA selected with target beads and $(His)_6$ beads were compared to determine signal/background ratios.

Following amplification, the PCR product was captured on MyOne-SA beads via the biotinylated antisense strand. 1.25 mL MyOne-SA beads (10 mg/mL) were washed twice with 0.5 mL 20 mM NaOH, once with 0.5 mL SB1T, resuspended in 2.5 mL 3 M NaCl, and stored at 4°C. 25 μL MyOne-SA beads (4 mg/mL in 3 M NaCl) were added to 50 μL double-stranded QPCR product and incubated 25°C, 5 minutes with mixing. The beads were washed once with SB1T, and the “sense” strand was eluted from the beads by adding 200 μL 20 mM NaOH, and incubating 37°C, 1 minute with mixing. The eluted strand was discarded and the beads were washed 3 times with SB1T and once with 16 mM NaCl.

SOMAmer sense strand was prepared with the appropriate nucleotide composition by primer extension from the immobilized antisense strand. The beads were resuspended in 20 μL primer extension reaction mix (1X KOD DNA Polymerase Buffer, 1.5 mM $MgCl_2$, 5 μM forward PCR primer, 0.125 U/μL KOD DNA Polymerase, 0.5 mM each dATP, MedCTP, dGTP, and either dTTP or dUTP analog) and incubated 68°C, 30 minutes with mixing. The beads were washed 3 times with SB1T, and the SOMAmer strand was eluted from the beads by adding 85 μL 20 mM NaOH, and incubating 37°C, 1 minute with mixing. 80 μL SOMAmer eluate was transferred to a new tube after magnetic separation, neutralized with 20 μL 80 mM HCl, and buffered with 5 μL 0.1 M HEPES, pH 7.5.

**Selection Strategy and Feedback.** The relative target protein concentration of the selection step was lowered each round in response to the S/B ratio as follows:

if $S/B < 10$, $[P]_{(i+1)} = [P]_i$
if $10 \leq S/B < 100$, $[P]_{(i+1)} = [P]_i / 3.2$
if $S/B \geq 100$, $[P]_{(i+1)} = [P]_i / 10$

where [P] = protein concentration and *i* = current round number. Target protein concentration was lowered by adjusting the mass of target protein beads (and $(His)_6$ beads) added to the selection step. After each selection round, the convergence state of the enriched DNA mixture was determined. 5 μL double-stranded QPCR product was diluted to 200 μL with 4 mM $MgCl_2$ containing 1X SYBR Green I. Samples were overlaid with 75 μL silicon oil and analyzed for convergence as follows.

**Nucleic Acid Reassociation Kinetics ($C_0t$) Assay.** The sample was thermal cycled with the following protocol: 3 cycles of 98°C, 1 minute, 85°C, 1 minute; 1 cycle of 93°C, 1 minute, 85°C, 15 minutes. During the 15 minutes at 85°C, fluorescent images were measured at 5-second intervals. The fluorescence intensity was plotted as a function of log (time) to evaluate the diversity of the sequences.

**Measurement of Equilibrium Binding Constants.** The equilibrium binding constants of the enriched libraries were measured using TALON bead partitioning. Radiolabled DNA was renatured by heating to 95°C for 3 minutes in SB1T and slowly

Nature Precedings : hdl:10101/npre.2010.4538.1 : Posted 14 Jun 2010

cooling to 37°C. Complexes were formed by mixing a low concentration of DNA (~1x10-$^{11}$ M) with a range of concentrations of target protein (1x10$^{-7}$ M to 1x 10$^{-12}$ M final) in SB1T, and incubating at 37°C. One-twelfth of each reaction was transferred to a nylon membrane and dried to determine total counts in each reaction. 25 μg of MyOne TALON beads (Invitrogen) was added to the remainder of each reaction and mixed at 37°C for one minute. Two-thirds of the reaction was then passed through a MultiScreen HV Plate (Millipore) under vacuum to separate protein-bound complexes from unbound DNA and washed with 100 μL SB1T. The nylon membrane and MultiScreen HV Plates were phosphorimaged and the amount of radioactivity in each sample quantified using a FUJI FLA-3000. The fraction of captured DNA was plotted as a function of protein concentration and a non-linear curve-fitting algorithm was used to extract equilibrium binding constants ($K_d$ values) from the data.

**Measurement of Dissociation Rate Constants.** The rate constant for SOMAmer:protein complex dissociation was determined for each aptamer by measuring the fraction of pre-formed aptamer:protein complexes that remain bound after addition of a competitor as a function of time. Radiolabled SOMAmer was renatured as described above. Approximately 5x10$^{-11}$ M SOMAmer was equilibrated in SB18T (40 mM HEPES, 100 mM NaCl, 5 mM KCl, 5 mM $MgCl_2$, 0.05% Tween-20 at pH 7.5) at 37°C with protein at a concentration 10X greater than the measured $K_d$ value. Samples were then diluted 2X with 40 nM non-labeled SOMAmer or 0.3 mM dextran sulfate in SB18T at various time points. Complexes were partitioned to separate free aptamer from protein:aptamer complexes. The type of partitioning was dependent upon the protein used since not all proteins bind to the same type of partitioning resin. For LBP and Histone H1.2, Zorbax PSM-300A (Agilent) resin was used for partitioning; for Kallistatin, MyOne TALON beads were used; for biotinylated-TIG2, MyOne Streptavidin beads were used. Complexes were captured on the appropriate resin, and the sample was passed through a MultiScreen HV Plate under vacuum. The samples were washed with SB18T. The MultiScreen HV Plates were phosphorimaged and the amount of radioactivity in each sample quantified using a FUJI FLA-3000. The fraction of complex remaining was plotted as a function of time, and the dissociation rate constant

Nature Precedings : hdl:10101/npre.2010.4538.1 : Posted 14 Jun 2010

was determined by fitting the data to an analytic expression for bimolecular dissociation kinetics using non-linear regression.

**Pull-down Assay.** 50% plasma samples were prepared by EDTA-plasma 2X in SB18T with 2 μM Z-Block_2 (the modified nucleotide sequence $(AC\text{-}BnBn)_7$-AC). The plasma spike samples were prepared by diluting 500 ng protein with the 50% plasma in SB17T (SB18T with 1 mM EDTA) with AEBSF and EGTA. The plasma samples were prepared by diluting the 50% plasma in SB17T with AEBSF and EGTA. The buffer spike samples were prepared by diluting 500 ng protein in SB17T with AEBSF and EGTA. These samples were combined with 10 pmoles of SOMAmer to give final concentrations of 10% plasma, 2 mM AEBSF, 0.5 mM EGTA, and 100 nM SOMAmer. Complexes were formed by incubated at incubating at 37°C for 45 minutes. 50 μL of a 20% slurry of Streptavidin agarose beads (ThermoFisher Scientific) was added to each sample and shaken for 10 minutes at room temperature. The samples were added to a MultiScreen HV Plate to perform washes under vacuum filtration. Each sample was washed one time quickly with 200 μL of SB17T, one time for one minute with 200 μL of 100 μM biotin in SB17T with shaking, one time with 200 μL of SB17T for one minute with shaking, and one time with 200 μL of SB17T for nine minutes with shaking. Proteins in the sample were labeled with both biotin and a fluorophore by incubating each samples in 100 μL of 1 mM EZ Link NHS-$PEO_4$-biotin (Pierce), 0.25 mM NHS-Alexa-647 (Invitrogen) in SB17T for five minutes with shaking. Each sample was washed one time with 200 μL of 20 mM glycine in SB17T and five times with 200 μL of SB17T, shaking each wash for one minute. The final wash was removed using centrifugation at 1000 rcf for 30 seconds. The beads were resuspended with 100 μL of SB17T. SOMAmers (complexed and free) were released from the beads by exposure under a BlackRay light source (UVP XX-Series Bench Lamps, 365 nm) for ten minutes with shaking. The samples were spun out of the plate by centrifugation at 1000 rcf for 30 seconds. 10 μL of each sample was removed and reserved as “Catch 1 eluate” for SDS-PAGE analysis. The remainder of the samples was captured through the biotinylated proteins by adding 20 μL of a 20% slurry of monomeric Avidin beads and shaking for ten minutes. The beads were transferred to a MultiScreen HV Plate and washed four times

Nature Precedings : hdl:10101/npre.2010.4538.1 : Posted 14 Jun 2010

with 100 μL of SB17T for one minute with shaking. The final wash was removed using centrifugation at 1000 rcf for 30 seconds. Proteins were eluted from the beads by incubating each sample with 100 μL of 2 mM biotin in SB17T for five minutes with shaking. Each eluate was transferred to 0.4 mg MyOne Streptavidin beads with a bound biotinylated-primer complementary to the 3' fixed region of the SOMAmer. The samples were incubated for five minutes with shaking to anneal the bead-bound fixed region to the SOMAmer complexes. Each sample was washed two times with 100 μL of 1XSB17T for one minute each with shaking and one time with 100 μL of 1XSB19T (5 mM HEPES, 100 mM NaCl, 5 mM KCl, 5 mM $MgCl_2$, 1mM EDTA, 0.05% Tween-20, pH 7.5) for one minute with shaking, all by magnetic separation. The complexes were eluted by incubating with 45 μL of 20 mM NaOH for two minutes with shaking. 40 μL of each eluate was added to 10 μL of 80 mM HCl with 0.05% Tween-20 in a new plate. 10 μL of each sample was removed and reserved as "Catch 2 aptamer-bound eluate" for SDS-PAGE analysis. Gel samples were run on NuPAGE 4-12% Bis Tris Glycine gels (Invitrogen) under reducing and denaturing conditions according to the manufacturer's directions. Gels were imaged on an Alpha Innotech FluorChem Q scanner in the Cy5 channel to image the proteins.

Nature Precedings : hdl:10101/npre.2010.4538.1 : Posted 14 Jun 2010

**SomaLogic Proteomic Affinity Assay Method**

All steps were performed at room temperature unless otherwise indicated.

**Sample thawing and plating.** Aliquots of 100% serum or EDTA- plasma, stored at -80°C, were thawed by incubating in a 25°C water bath for ten minutes. After thawing the samples were stored on ice during mixing and prior to sample dilution. Samples were mixed by gentle vortexing (setting # 4 on Vortex Genie) for 8 seconds. A 20% sample solution was prepared by transferring 16 μL of thawed sample into 96-well plates (Hybaid Omnitube 0.3 mL, ThermoFisher Scientific) containing 64 µL per well of the appropriate sample diluent at 4°C. Sample diluent for serum was 0.8x SB17 with 0.6 mM $MgCl_2$, 2 mM EGTA, 2 μM Z-Block_2, 0.05% Tween and for EDTA-plasma was 0.8x SB18 with 0.8 mM MgCl2, 2 mM EGTA, 2 μM Z-Block_2, 0.05% Tween. This plate was stored on ice until the next sample dilution steps were initiated.

**Preparation of 10%, 1% and 0.03% SOMAmer Solutions.** SOMAmers were grouped into three unique mixes. The placing of a SOMAmer within a mix was empirically determined by assaying a dilution series of serum or plasma with each SOMAmer and identifying the sample dilution that gave the largest linear range of signal. The segregation of SOMAmers and mixing with different dilutions of sample (10%, 1% or 0.03%) allow the assay to span a $10^7$-fold range of protein concentration. The composition of the custom SOMAmer mixes was slightly different between plasma and serum as expected due to variation in protein composition of these two media. The custom stock SOMAmer solutions for 10%, 1% and 0.03% serum and plasma were prepared and stored at 8x concentration in SB17T.

For each assay run, the three 8x SOMAmer solutions were diluted separately 1:4 into SB17T to achieve 2x concentration. Each diluted SOMAmer master mix was heated to 95°C for five minutes and then to 37°C for 15 minutes. 55 µL of each 2x SOMAmer mix was manually pipetted into a 96-well plate resulting in three plates with 10%, 1% or 0.03% SOMAmer mixes. After mixing with sample, the final individual SOMAmer concentration ranged from 0.25-4 nM for serum, 0.5nM for plasma.

**Equilibration.** A 2% sample plate was prepared by diluting the 20% sample 1:10 into SB17T using the Beckman Coulter Biomek $Fx^P$. A 0.06% sample plate was prepared by diluting the 2% sample plate 1:31 into SB17T. The three sample dilutions were then transferred to their respective SOMAmer solutions by adding 55 µL of the sample to 55 µL of the appropriate 2x SOMAmer mix. The plates were sealed with a foil seal (Plate Bio-Rad Microseal 'F' Foil, MJ Research) and incubated for 3.5 hours at 37°C.

**Preparation of Catch 1 Bead Plates.** 133.3 µL of a 7.5% Streptavidin-agarose bead slurry in SB17T was added to each well of three pre-washed 0.45 um filter plates. Each well of beads was washed once with 200 µL SB17T using vacuum filtration to remove the wash and then resuspended in 200 µL SB17T.

**Catch 1 Bead Capture.** All subsequent steps were performed by the Beckman Coulter Biomek $Fx^P$ robot unless otherwise noted. After the 3.5 hour equilibration, 100 µL of the 10%, 1% and 0.03% equilibration binding reactions was transferred to their respective Catch 1 Streptavidin agarose filter plates and incubated with shaking for ten minutes. Unbound solution was removed via vacuum filtration. Each set of Catch 1 beads was washed with 190 µL of 100 µM biotin in SB17T and then 190 µL of SB17T using vacuum filtration to remove the wash.

Nature Precedings : hdl:10101/npre.2010.4538.1 : Posted 14 Jun 2010

190 µL SB17T was added to each well in the Catch 1 plates and incubated with shaking for ten minutes at 25°C. The wash was removed via vacuum filtration and the bottom of the filter plates blotted to remove droplets using the on-deck blot station.

**Biotinylation of Proteins.** An aliquot of 100 mM NHS-PEO4-biotin in DMSO was thawed at 37°C for six minutes and diluted to 1 mM with SB17T at pH 7.25. 100 µL of the NHS-PEO4-biotin was added to each well of each Catch 1 filter plate and incubated with shaking for five minutes. Each biotinylation reaction was quenched by adding 150 µL of 20 mM glycine in SB17T to the Catch 1 plates with the NHS-PEO4-biotin. Plates were incubated for one minute with shaking, vacuum filtrated, and 190 µL 20 mM glycine SB17T was added to each well in the plate. The plates were incubated for one minute, shaking before removal by vacuum filtration. 190 µL of SB17T was added to each well and removed by vacuum filtration. The wells of the Catch 1 plates were subsequently washed three times by adding 190 µL SB17T, incubating for one minute with shaking followed by vacuum filtration. After the last wash the plates were centrifuged at 1000 rpm for one minute over a 1 mL deep-well plate to remove extraneous volume before elution. Centrifugation was performed off deck.

**Kinetic Challenge and Photo-Cleavage.** 85 µL of 10 mM $DxSO_4$ in SB17T was added to each well of the filter plates. The filter plates were placed onto a Thermal Shaker (Eppendorf) under a BlackRay light source and irradiated for ten minutes with shaking. The photo-cleaved solutions were sequentially eluted from each Catch 1 plate into a common deep well plate by centrifugation at 1000 rpm for one minute each.

**Catch 2 Bead Capture.** In bulk, MyOne-Streptavidin C1 beads were washed two times for 5 minutes each with equal volume of 20 mM NaOH and three times with an equal volume of SB17T. Beads were resuspended in SB17T to a concentration of 10 mg/mL. After resuspension, 50 µL of this solution was manually pipetted into each well of a 96-well plate and stored at 4°C until Catch 2. During Catch 2, the wash supernatant was removed via magnetic separation. All of the photo-cleaved eluate was pipetted onto the MyOne magnetic beads and incubated with shaking for five minutes at 25°C. The supernatant was removed from the MyOne beads via magnetic separation and 75 µL of SB17T was transferred to each well. The plate was mixed for one minute at 37°C with shaking and then 75 µL of 60% glycerol (in SB17T) at 37°C was transferred to each well. The plate was mixed for another minute at 37°C with shaking. The wash was removed via magnetic separation. These washes were repeated two more times. After

Nature Precedings : hdl:10101/npre.2010.4538.1 : Posted 14 Jun 2010

removal of the third glycerol wash from the MyOne beads, 150 µL of SB17T was added to each well and the plates incubated at 37°C with shaking for one minute before removal by magnetic separation. The MyOne beads were washed a final time using 150 µL SB19T with incubation for one minute, prior to magnetic separation.

**Catch 2 Bead Elution and Neutralization.** SOMAmers were eluted from MyOne beads by incubating each well of beads with 105 µL of 100 mM CAPSO pH 10, 1 M NaCl, 0.05% Tween with shaking for five minutes. 90 µL of each eluate was transferred during magnetic separation to a new 96-well plate containing 10 µL of 500 mM HCl, 500 mM HEPES, 0.05% Tween-20.

**Hybridization.** 20 µL of each neutralized catch 2 eluate was transferred to a new 96-well plate and 5 µL of 10x Agilent Block (Oligo aCGH/ChIP-on-chip Hybridization Kit, Large Volume, Agilent 5188-5380), containing a 10x spike of hybridization controls (10 Cy3 SOMAmers) was added to each well. After removing the plate from the robot, 25 µL of 2x Agilent Hybridization buffer (Oligo aCGH/ChIP-on-chip Hybridization Kit) was manually pipetted to the each well of the plate containing the neutralized samples and blocking buffer. 40 µL of this solution was manually pipetted into each "well" of the hybridization gasket slide (Hybridization Gasket Slide - 8 microarray per slide format, Agilent). Custom Agilent microarray slides containing 10 probes per array complementary to 40 nucleotide selected region of each SOMAmer with a 20x dT linker were placed onto the gasket slides according to the manufacturer's protocol. Each assembly (Hybridization Chamber Kit - SureHyb enabled, Agilent) was tightly clamped and loaded into a hybridization oven for 19 hours at 60°C rotating at 20 rpm.

**Post Hybridization Washing.** Approximately 400 mL Wash Buffer 1 (Oligo aCGH/ChIP-on-chip Wash Buffer 1, Agilent) was placed into each of two separate glass staining dishes. Six of the twelve slide/gasket assemblies were sequentially disassembled into the first staining dish containing Wash Buffer 1. Once disassembled, the slide was quickly transferred into a slide rack in a second staining dish containing Wash Buffer 1. The slides were incubated for five minutes in Wash Buffer 1 with mixing via magnetic stir bar. The slide rack was then transferred to the 37°C Wash Buffer 2 (Oligo aCGH/ChIP-on-chip Wash Buffer 2, Agilent) and allowed to incubate for five minutes with stirring. The slide rack was transferred to a fourth staining dish containing acetonitrile and incubated for five minutes with stirring.

Nature Precedings : hdl:10101/npre.2010.4538.1 : Posted 14 Jun 2010

**Microarray Imaging.** The microarray slides were imaged with a microarray scanner (Agilent G2565CA Microarray Scanner System) in the Cy3-channel at 5 μm resolution at 100% PMT setting and the XRD option enabled at 0.05. The resulting tiff images were processed using Agilent feature extraction software version 10.5.1.1 with the GE1_105_Dec08 protocol.

#### Serum and Plasma Reproducibility Studies

For each plate, five aliquots of plasma or serum from 18 individuals were thawed and plated as described below. Six wells containing only buffer were run on every plate. Serum and plasma samples were run on separate plates because they require slightly different buffers as indicated above. Three plates of each sample type were run over the course of several days and included using different lots of buffers and other reagents that might be expected to change within a large study.

Nature Precedings : hdl:10101/npre.2010.4538.1 : Posted 14 Jun 2010

#### Limits of Quantification (LOQ) Experiment

For the LOQ experiments, four different sets of protein mixes were prepared for each of the three SOMAmer mixes, 10%, 1% or 0.03%, for a total of 12 mixes and 356 proteins. The proteins for each mix were chosen to avoid combining known protein binding partners and known protease-substrate pairs.

The proteins were diluted into SB17T containing 2 μM Z-Block_2 so that each protein was at a final concentration of 20 nM. The protein solutions were serially diluted 15.8-fold into SB17T for a total of six points (lowest concentration: 20.3 fM). All of the protein preparation was maintained on ice. Eight replicate protein titrations per set were pipetted into 96-well plates.

#### Clinical Data Processing

**Assay Normalization.** Assay normalization was performed to reduce signal variation potentially introduced during the assay. Each sample in a study was normalized using a set of SOMAmers that have the lowest overall relative signal variation across all samples within a study. For each normalization SOMAmer, its median value was calculated from all samples in the study, and together these median values were used to calculate a scaling factor for each individual sample. The scaling factor was the mean of a series of values, one for each normalization SOMAmer, calculated as the sample signal divided by the median signal for the study.  When applied to a sample, this procedure brings the signals corresponding to SOMAmers in the normalization set closer to the median values across the assay, and reduces the observed variation between replicate samples for all SOMAmers.  Because the assay splits each clinical sample into three dilutions, assay normalization was performed separately on the three SOMAmer groups corresponding to the 10%, 1%, and 0.1% dilutions.  Dilution normalization applies the same constant factor to every signal in that dilution from any given sample.  This factor varied between samples in the range from 0.8 to 1.2, and was typically within 10% of unity.

**Between Run Calibration.** To compare samples between assay runs with slightly different conditions, we have applied calibration to the individual SOMAmer*s* signals.  For this we apply a multiplicative correction factor specific to each SOMAmer, but invariant with respect to the sample (in contrast to normalization in which the factor was specific to the sample and did not vary from SOMAmer to SOMAmer within a sample).  To calculate the calibration constant for each SOMAmer, we measure a set of eight calibrator samples derived from blood from the same individual in each sample set. From these calibrator sample measurements, we can standardize the signals from a sample within one run by applying the calibration coefficient for each SOMAmer that scales the median calibrator signal of that aptamer to a reference standard for that aptamer.

Nature Precedings : hdl:10101/npre.2010.4538.1 : Posted 14 Jun 2010

**Chronic Kidney Disease**

CKD serum samples were collected by the Rogosin Institute for the clinical study entitled “Quantification of inflammatory and immune mediators of CKD in patient serum, whole blood and urine: Correlation with CKD disease stage progression”.  Both the original study and the biomarker study reported here were approved by the Institutional Review Board at Weil Medical College of Cornell University.  The clinical study design specified that samples be collected from 25 healthy controls with no renal disease and 25 subjects at each stage of CKD (1-5) for a total of 150 subjects.  Our biomarker study included serum samples from 55 subjects that were available at the time this study was conducted. Table 11 summarizes the population demographics. The groups are well matched by gender, ethnicity, age, weight and body mass index. Renal function, measured by the estimated glomerular filtration rate (eGFR, calculated with the MDRD formula for creatinine clearance[3]), is substantially different in the two groups (see figure S15).

Table 11. CKD Population Demographics

| | Early stage CKD | Late stage CKD |
|---|---|---|
| N (total = 42) | 11 | 31 |
| Gender %F (F/M) | 33% (4/11) | 45% (14/31) |
| Ethnicity (A/B/C/H)[a] | 1/5/1/4 | 2/9/0/20 |
| Age (avg. yrs) | 62 [51-68] | 67 [57-77] |
| Wt. (avg. kg) | 89 [73-98] | 88 [75-104] |
| BMI (avg.) | 30.5 [26.6-36.5] | 31.8 [27.1-36.6] |
| eGFR (median)[b] | 70 [62-97] | 25 [7-49] |

[a]A, Asian; B, African American; C, Caucasian; H, Hispanic

[b]Estimated glomerular filtration from creatinine clearance (MDRD formula) ml/min/m$^2$

Nature Precedings : hdl:10101/npre.2010.4538.1 : Posted 14 Jun 2010

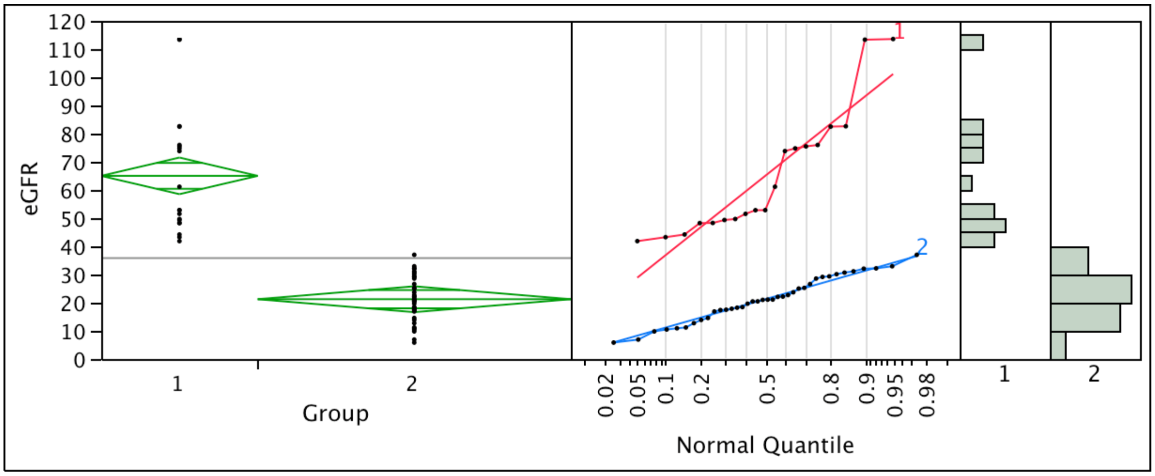

**Figure S15.** Renal function (eGFR) in diabetic patients stratified into 18 early stage subjects (CKD Stage 1-3) and 37 late stage subjects (CKD Stage 4-5).

### Supplementary References

1. Schneider, D. et al. *Multiplexed Analyses of Test Samples.* US2009/0042206. USA. (2009).
2. Zichi, D. et al. *Method for Generating Aptamers with Improved Off-Rates.* US2009/0004667. USA. (2009).
3. Levey, A. S. et al. A more accurate method to estimate glomerular filtration rate from serum creatinine: a new prediction equation. Modification of Diet in Renal Disease Study Group. *Ann. Intern. Med.* **130**, 461-70 (1999).

Nature Precedings : hdl:10101/npre.2010.4538.1 : Posted 14 Jun 2010

Table 2. Protein Target Menu

| Target | Uni-Prot Protein Name | Uni-Prot Acc # | CKD |
|---|---|---|---|
| 14-3-3 protein zeta/delta | 14-3-3 protein zeta/delta | P63104 | |
| 4-1BB | Tumor necrosis factor receptor superfamily member 9 | Q07011 | Y |
| 4-1BB ligand | Tumor necrosis factor ligand superfamily member 9 | P41273 | |
| 6Ckine | Small-inducible cytokine A21 | O00585 | Y |
| α1-Antichymotrypsin | Alpha-1-antichymotrypsin | P01011 | Y |
| α1-Antitrypsin | Alpha-1-antitrypsin | P01009 | |
| α2-Antiplasmin | Alpha-2-antiplasmin | P08697 | Y |
| α2-HS-Glycoprotein | Alpha-2-HS-glycoprotein | P02765 | |
| α2-Macroglobulin | Alpha-2-macroglobulin | P01023 | |
| ABL1 | Proto-oncogene tyrosine-protein kinase ABL1 | P00519 | Y |
| ABL2 | Tyrosine-protein kinase ABL2 | P42684 | Y |
| ACE-2 | Angiotensin-converting enzyme 2 | Q9BYF1 | Y |
| Activated Protein C | Vitamin K-dependent protein C (activated form) | P04070 | Y |
| Activin A | Inhibin beta A chain | P08476 | Y |
| Activin RIB | Activin receptor type-1B | P36896 | Y |
| ADAM 9 | Disintegrin and metalloproteinase domain-containing protein 9 | Q13443 | |
| ADAMTS-1 | ADAMTS-1 | Q9UHI8 | |
| ADAMTS-4 | ADAMTS-4 | O75173 | Y |
| ADAMTS-5 | ADAMTS-5 | Q9UNA0 | Y |
| ADAMTS-13 | ADAMTS-13 | Q76LX8 | Y |
| Adenosylhomocysteinase | Adenosylhomocysteinase | P23526 | |
| Adiponectin | Adiponectin | Q15848 | |
| aFGF | Heparin-binding growth factor 1 | P05230 | Y |
| AGGF1 | Angiogenic factor with G patch and FHA domains 1 | Q8N302 | Y |
| Aggrecan | Aggrecan core protein | P16112 | Y |
| AgRP | Agouti-related protein | O00253 | Y |
| AIF-1 | Allograft inflammatory factor 1 | P55008 | Y |
| AIP | AH receptor-interacting protein | O00170 | |
| Albumin | Serum albumin | P02768 | |
| ALCAM | Activated leukocyte cell adhesion molecule | Q13740 | Y |
| aldolase A | Fructose-bisphosphate aldolase A | P04075 | |
| ALK-1 | Serine/threonine-protein kinase receptor R3 | P37023 | Y |
| Alkaline phosphatase, bone | Alkaline phosphatase, tissue-nonspecific isozyme | P05186 | Y |
| ALT | Alanine aminotransferase 1 | P24298 | |
| Aminoacylase-1 | Aminoacylase-1 | Q03154 | Y |
| amphiregulin | Amphiregulin | P15514 | Y |
| amyloid precursor protein | Amyloid beta A4 protein | P05067 | |
| Angiogenin | Angiogenin | P03950 | Y |
| Angiopoietin-1 | Angiopoietin-1 | Q15389 | Y |
| Angiopoietin-2 | Angiopoietin-2 | O15123 | Y |
| Angiopoietin-4 | Angiopoietin-4 | Q9Y264 | Y |
| Angiostatin | Angiostatin | P00747 | |
| Angiotensinogen | Angiotensinogen | P01019 | Y |
| Angplt4 | Angiopoietin-related protein 4 | Q9BY76 | |
| Angptl3 | Angiopoietin-related protein 3 | Q9Y5C1 | Y |
| Antithrombin III | Antithrombin-III | P01008 | Y |
| Apo A-I | Apolipoprotein A-I | P02647 | Y |
| Apo B | Apolipoprotein B-100 and Apolipoprotein B-48 | P04114 | Y |
| Apo E | Apolipoprotein E | P02649 | Y |
| Apo E2 | Apolipoprotein E (isoform E2) | P02649 | Y |
| Apo E3 | Apolipoprotein E (isoform E3) | P02649 | Y |
| Apo E4 | Apolipoprotein E (isoform E4) | P02649 | Y |
| ApoE R2 | Low-density lipoprotein receptor-related protein 8 | Q14114 | Y |
| APRIL | Tumor necrosis factor ligand superfamily member 13 | O75888 | Y |
| Arginase-1 | Arginase-1 | P05089 | Y |
| ARID3A | AT-rich interactive domain-containing protein 3A | Q99856 | |
| ARSB | Arylsulfatase B | P15848 | Y |
| Artemin | Artemin | Q5T4W7 | Y |
| Arylsulfatase A | Arylsulfatase A | P15289 | |

Nature Precedings : hdl:10101/npre.2010.4538.1 : Posted 14 Jun 2010

| Target | Uni-Prot Protein Name | Uni-Prot Acc # | CKD |
|---|---|---|---|
| ASAH2 | Neutral ceramidase | Q9NR71 | Y |
| ASAHL | N-acylethanolamine-hydrolyzing acid amidase | Q02083 | |
| ASGPR1 | Asialoglycoprotein receptor 1 | P07306 | |
| Aurora B | Serine/threonine-protein kinase 12 | Q96GD4 | Y |
| Aurora kinase A | Serine/threonine-protein kinase 6 | O14965 | Y |
| Azurocidin | Azurocidin | P20160 | Y |
| β2-Microglobulin | Beta-2-microglobulin | P61769 | Y |
| B7 | T-lymphocyte activation antigen CD80 | P33681 | Y |
| β-Endorphin | Beta-endorphin | P01189 | Y |
| β-NGF | Beta-nerve growth factor | P01138 | Y |
| BAFF | Tumor necrosis factor ligand superfamily member 13B | Q9Y275 | Y |
| BAFF Receptor | Tumor necrosis factor receptor superfamily member 13C | Q96RJ3 | |
| BARK1 | Beta-adrenergic receptor kinase 1 | P25098 | Y |
| BCA-1 | Small-inducible cytokine B13 | O43927 | |
| BCAM | Lutheran blood group glycoprotein | P50895 | Y |
| Bcl-2 | Apoptosis regulator Bcl-2 | P10415 | Y |
| BCL2A1 | Bcl-2-related protein A1 | Q16548 | Y |
| BCMA | Tumor necrosis factor receptor superfamily member 17 | Q02223 | Y |
| BDNF | Brain-derived neurotrophic factor | P23560 | Y |
| bFGF | Heparin-binding growth factor 2 | P09038 | Y |
| bFGF-R | Basic fibroblast growth factor receptor 1 | P11362 | Y |
| βIGH3 | Transforming growth factor-beta-induced protein ig-h3 | Q15582 | Y |
| Biglycan | Biglycan | P21810 | Y |
| BMP RII | Bone morphogenetic protein receptor type-2 | Q13873 | Y |
| BMP-1 | Bone morphogenetic protein 1 | P13497 | Y |
| BMP10 | Bone morphogenetic protein 10 | O95393 | |
| BMP-14 | Growth/differentiation factor 5 | P43026 | Y |
| BMP-6 | Bone morphogenetic protein 6 | P22004 | |
| BMP-7 | Bone morphogenetic protein 7 | P18075 | Y |
| BMPER | BMP-binding endothelial regulator protein | Q8N8U9 | Y |
| BMPR1A | Bone morphogenetic protein receptor type IA | P36894 | |
| BMX | Cytoplasmic tyrosine-protein kinase BMX | P51813 | Y |
| BNP-32 | Brain natriuretic peptide 32 | P16860 | |
| Bone proteoglycan II | Decorin | P07585 | Y |
| BPI | Bactericidal permeability-increasing protein | P17213 | Y |
| Brevican | Brevican core protein | Q96GW7 | |
| BSP | Bone sialoprotein 2 | P21815 | Y |
| BTK | Tyrosine-protein kinase BTK | Q06187 | Y |
| C1q | Complement C1q subcomponent subunits A, B, and C | P02747,P02746,P02745 | Y |
| C1r | Complement C1r subcomponent | P00736 | Y |
| C1s | Complement C1s subcomponent | P09871 | |
| C2 | Complement C2 | P06681 | Y |
| C3 | Complement C3 | P01024 | Y |
| C3a | C3a anaphylatoxin | P01024 | Y |
| C3adesArg | C3a anaphylatoxin des Arginine | P01024 | Y |
| C3b | Complement C3b | P01024 | Y |
| C3d | Complement C3d fragment | P01024 | Y |
| C4 | Complement C4-A and Complement C4-B | P0C0L4, P0C0L5 | Y |
| C4b | C4b-A | P0C0L4 P0C0L5 | Y |
| C5 | Complement C5 | P01031 | Y |
| C5a | C5a anaphylatoxin | P01031 | Y |
| C5b,6 Complex | Complement C5b, and Complement component C6 | P01031 P13671 | Y |
| C6 | Complement component C6 | P13671 | Y |
| C7 | Complement component C7 | P10643 | Y |
| C8 | Complement component C8 alpha, beta, and gamma chains | P07357,P07358,P07360 | Y |
| C9 | Complement component C9 | P02748 | Y |
| Cadherin-1 | Epithelial cadherin | P12830 | Y |
| Cadherin-2 | Neural cadherin | P19022 | |
| Cadherin-5 | Vascular endothelial cadherin | P33151 | Y |
| Cadherin-6 | Kidney cadherin | P55285 | |
| Cadherin-12 | Brain cadherin | P55289 | Y |

Nature Precedings : hdl:10101/npre.2010.4538.1 : Posted 14 Jun 2010

Nature Precedings : hdl:10101/npre.2010.4538.1 : Posted 14 Jun 2010

| Target | Uni-Prot Protein Name | Uni-Prot Acc # | CKD |
|---|---|---|---|
| Calcineurin B α | Calcineurin subunit B type 1 | P63098 | |
| Calpain I | Calpain-1 catalytic subunit and Calpain small subunit 1 | P07384, P04632 | Y |
| Calpastatin | Calpastatin | P20810 | Y |
| CAMK1 | Calcium/calmodulin-dependent protein kinase type 1 | Q14012 | |
| CAMK1D | Calcium/calmodulin-dependent protein kinase type 1D | Q8IU85 | Y |
| CAMK2A | Calcium/calmodulin-dependent protein kinase type II alpha chain | Q9UQM7 | Y |
| CAMK2B | Calcium/calmodulin-dependent protein kinase type II beta chain | Q13554 | Y |
| CAMK2D | Calcium/calmodulin-dependent protein kinase type II delta chain | Q13557 | Y |
| CaMKK α | Calcium/calmodulin-dependent protein kinase kinase 1 | Q8N5S9 | |
| Carbonic anhydrase III | Carbonic anhydrase 3 | P07451 | |
| Carbonic anhydrase IV | Carbonic anhydrase 4 | P22748 | Y |
| Carbonic anhydrase VI | Carbonic anhydrase 6 | P23280 | Y |
| Carbonic anhydrase VII | Carbonic anhydrase 7 | P43166 | Y |
| Carbonic anhydrase IX | Carbonic anhydrase 9 | Q16790 | |
| Carbonic anhydrase XIII | Carbonic anhydrase 13 | Q8N1Q1 | Y |
| Carbonic anhydrase-related protein X | Carbonic anhydrase-related protein 10 | Q9NS85 | Y |
| Cardiotrophin-1 | Cardiotrophin-1 | Q16619 | Y |
| Carnosine dipeptidase 1 | Beta-Ala-His dipeptidase | Q96KN2 | |
| Caspase-3 | Caspase-3 (pro form) | P42574 | |
| Catalase | Catalase | P04040 | Y |
| Cathepsin A | Lysosomal protective protein | P10619 | Y |
| Cathepsin B | Cathepsin B | P07858 | Y |
| Cathepsin C | Dipeptidyl-peptidase 1 | P53634 | |
| Cathepsin D | Cathepsin D | P07339 | |
| Cathepsin E | Cathepsin E | P14091 | |
| Cathepsin G | Cathepsin G | P08311 | Y |
| Cathepsin H | Cathepsin H | P09668 | |
| Cathepsin S | Cathepsin S | P25774 | Y |
| Cathepsin V | Cathepsin L2 | O60911 | Y |
| CCL1 | Small-inducible cytokine A1 | P22362 | Y |
| CCL28 | Small-inducible cytokine A28 | Q9NRJ3 | Y |
| CD22 | B-cell receptor CD22 | P20273 | Y |
| CD23 | Low affinity immunoglobulin epsilon Fc receptor | P06734 | Y |
| CD30 | Tumor necrosis factor receptor superfamily member 8 | P28908 | Y |
| CD30 Ligand | Tumor necrosis factor ligand superfamily member 8 | P32971 | Y |
| CD36 ANTIGEN | Platelet glycoprotein 4 | P16671 | Y |
| CD39 | Ectonucleoside triphosphate diphosphohydrolase 1 | P49961 | Y |
| CD40 ligand, soluble | CD40 ligand | P29965 | |
| CD48 | CD48 antigen | P09326 | Y |
| CD5L | CD5 antigen-like | O43866 | Y |
| CD70 | CD70 antigen | P32970 | |
| CD97 | CD97 antigen | P48960 | Y |
| CD109 | CD109 antigen | Q6YHK3 | |
| CDC37 | Hsp90 co-chaperone Cdc37 | Q16543 | |
| CDK1/cyclin B | Cell division control protein 2 homolog, G2/mitotic-specific cyclin-B1 Complex | P06493, P14635 | Y |
| CDK2/cyclin A | Cell division protein kinase 2, Cyclin-A2 Complex | P24941, P20248 | Y |
| CDK5/p35 | Cell division protein kinase 5, Cyclin-dependent kinase 5 activator 1, p35 Complex | Q00535, Q15078 | Y |
| CDK8/cyclin C | Cell division protein kinase 8, Cyclin-C Complex | P49336, P24863 | Y |
| CEA | Carcinoembryonic antigen-related cell adhesion molecule 5 | P06731 | |
| Chemerin | Retinoic acid receptor responder protein 2 | Q99969 | Y |
| Chitotriosidase-1 | Chitotriosidase-1 | Q13231 | |
| Chk1 | Serine/threonine-protein kinase Chk1 | O14757 | Y |
| Chk2 | Serine/threonine-protein kinase Chk2 | O96017 | Y |
| CHL1 | Neural cell adhesion molecule L1-like protein | O00533 | |
| Chordin-Like 1 | Chordin-like protein 1 | Q9BU40 | Y |
| Chymase | Chymase | P23946 | Y |
| CK-BB | Creatine kinase B-type | P12277 | Y |
| CK-MB | Creatine kinase B-type, Creatine kinase M-type | P12277, P06732 | |
| CK-MM | Creatine kinase M-type | P06732 | Y |

| Target | Uni-Prot Protein Name | Uni-Prot Acc # | CKD |
|---|---|---|---|
| Ck-β-8-1 | Small-inducible cytokine A23 | P55773 | Y |
| CLF-1/CLC Complex | Cytokine receptor-like factor 1 and Cardiotrophin-like cytokine factor 1 | O75462, Q9UBD9 | Y |
| CN166 | UPF0568 protein C14orf166 | Q9Y224 | |
| CNTF | Ciliary neurotrophic factor | P26441 | Y |
| CNTFR α | Ciliary neurotrophic factor receptor alpha | P26992 | Y |
| Coagulation Factor V | Coagulation factor V | P12259 | Y |
| Coagulation Factor VII | Coagulation factor VII | P08709 | Y |
| Coagulation Factor IX | Coagulation factor IX | P00740 | Y |
| Coagulation Factor IXab | Coagulation factor IX (activated form) | P00740 | Y |
| Coagulation Factor X | Coagulation factor X | P00742 | Y |
| Coagulation Factor Xa | Coagulation factor X (activated form) | P00742 | Y |
| Coagulation Factor XI | Coagulation factor XI | P03951 | Y |
| COLEC12 | Collectin-12 | Q5KU26 | Y |
| COMMD7 | COMM domain-containing protein 7 | Q86VX2 | Y |
| complement factor H-related 5 | Complement factor H-related protein 5 | Q9BXR6 | |
| Contactin-1 | Contactin-1 | Q12860 | Y |
| Contactin-2 | Contactin-2 | Q02246 | Y |
| Contactin-4 | Contactin-4 | Q8IWV2 | Y |
| Contactin-5 | Contactin-5 | O94779 | Y |
| COX-2 | Prostaglandin G/H synthase 2 | P35354 | Y |
| Cripto | Teratocarcinoma-derived growth factor 1 | P13385 | Y |
| CRISP-3 | Cysteine-rich secretory protein 3 | P54108 | Y |
| CRP | C-reactive protein | P02741 | |
| Cryptic | Cryptic protein | Q9GZR3 | Y |
| CSK | Tyrosine-protein kinase CSK | P41240 | |
| CSK21 | Casein kinase II subunit alpha | P68400 | Y |
| CTACK | Small-inducible cytokine A27 | Q9Y4X3 | Y |
| CTGF | Connective tissue growth factor | P29279 | Y |
| CTLA-4 | Cytotoxic T-lymphocyte protein 4 | P16410 | Y |
| CXCL16, soluble | Small-inducible cytokine B16 | Q9H2A7 | Y |
| Cyclophilin A | Peptidyl-prolyl cis-trans isomerase A | P62937 | |
| Cystatin C | Cystatin-C | P01034 | Y |
| Cystatin D | Cystatin-D | P28325 | |
| Cystatin F | Cystatin-F | O76096 | Y |
| Cystatin M | Cystatin-M | Q15828 | Y |
| Cystatin S | Cystatin-S | P01036 | |
| Cystatin SN | Cystatin-SN | P01037 | Y |
| Cytochrome c | Cytochrome c | P99999 | Y |
| Cytochrome P450 3A4 | Cytochrome P450 3A4 | P08684 | Y |
| DAN | Neuroblastoma suppressor of tumorigenicity 1 | P41271 | Y |
| DAPK2 | Death-associated protein kinase 2 | Q9UIK4 | |
| DARPP-32 | Protein phosphatase 1 regulatory subunit 1B | Q9UD71 | Y |
| DC-SIGN | CD209 antigen | Q9NNX6 | Y |
| DC-SIGNR | C-type lectin domain family 4 member M | Q9H2X3 | Y |
| DEAD-box protein 19B | ATP-dependent RNA helicase DDX19B | Q9UMR2 | Y |
| Dectin-1 | C-type lectin domain family 7 member A | Q9BXN2 | |
| Desmoglein-1 | Desmoglein-1 | Q02413 | Y |
| Discoidin domain receptor 1 | Epithelial discoidin domain-containing receptor 1 | Q08345 | |
| Discoidin domain receptor 2 | Discoidin domain-containing receptor 2 | Q16832 | |
| Dkk-1 | Dickkopf-related protein 1 | O94907 | Y |
| Dkk-3 | Dickkopf-related protein 3 | Q9UBP4 | |
| Dkk-4 | Dickkopf-related protein 4 | Q9UBT3 | Y |
| DLC8 | Dynein light chain 1, cytoplasmic | P63167 | |
| DLL4 | Delta-like protein 4 | Q9NR61 | Y |
| DLRB1 | Dynein light chain roadblock-type 1 | Q9NP97 | |
| DMP1 | Dentin matrix acidic phosphoprotein 1 | Q13316 | |
| dopa decarboxylase | Aromatic-L-amino-acid decarboxylase | P20711 | Y |
| DPP2 | Dipeptidyl-peptidase 2 | Q9UHL4 | |
| DRG-1 | Vacuolar protein sorting-associated protein VTA1 homolog | Q9NP79 | Y |
| DRR1 | Protein FAM107A | O95990 | Y |

Nature Precedings : hdl:10101/npre.2010.4538.1 : Posted 14 Jun 2010

Nature Precedings : hdl:10101/npre.2010.4538.1 : Posted 14 Jun 2010

| Target | Uni-Prot Protein Name | Uni-Prot Acc # | CKD |
|---|---|---|---|
| Dtk | Tyrosine-protein kinase receptor TYRO3 | Q06418 | Y |
| DYRK3 | Dual specificity tyrosine-phosphorylation-regulated kinase 3 | O43781 | |
| ECM1 | Extracellular matrix protein 1 | Q16610 | Y |
| EDA | Ectodysplasin-A (splice variant A2) | Q92838 | Y |
| EDAR | Tumor necrosis factor receptor superfamily member EDAR | Q9UNE0 | Y |
| EF-1-β | Elongation factor 1-beta | P24534 | |
| EF-1-γ | Elongation factor 1-gamma | P26641 | |
| EG-VEGF | Prokineticin-1 | P58294 | Y |
| eIF-4H | Eukaryotic translation initiation factor 4H | Q15056 | |
| eIF-5 | Eukaryotic translation initiation factor 5 | P55010 | Y |
| eIF-5A-1 | Eukaryotic translation initiation factor 5A-1 | P63241 | |
| Elastase | Leukocyte elastase | P08246 | Y |
| EMAP-2 | Endothelial monocyte-activating polypeptide 2 | Q12904 | Y |
| EMMPRIN | Basigin | P35613 | |
| ENA-78 | C-X-C motif chemokine 5 | P42830 | Y |
| Endocan | cDNA FLJ50870, moderately similar to Endothelial cell-specific molecule 1 | Q3V4E3 | |
| Endostatin | Endostatin | P39060 | Y |
| Endothelin-converting enzyme 1 | Endothelin-converting enzyme 1 | P42892 | |
| Enterokinase | Enteropeptidase | P98073 | Y |
| Eotaxin | Eotaxin | P51671 | Y |
| Eotaxin-2 | Small-inducible cytokine A24 | O00175 | Y |
| EphA1 | Ephrin type-A receptor 1 | P21709 | Y |
| EPHA3 | Ephrin type-A receptor 3 | P29320 | Y |
| EphA5 | Ephrin type-A receptor 5 | P54756 | |
| EphB4 | Ephrin type-B receptor 4 | P54760 | |
| Ephrin-A4 | Ephrin-A4 | P52798 | Y |
| Ephrin-A5 | Ephrin-A5 | P52803 | Y |
| Ephrin-B3 | Ephrin-B3 | Q15768 | Y |
| Epithelial cell kinase | Ephrin type-A receptor 2 | P29317 | Y |
| EPO-R | Erythropoietin receptor | P19235 | Y |
| ER | Estrogen receptor | P03372 | Y |
| ERBB1 | Epidermal growth factor receptor | P00533 | Y |
| ERBB2 | Receptor tyrosine-protein kinase erbB-2 | P04626 | Y |
| ERBB3 | Receptor tyrosine-protein kinase erbB-3 | P21860 | Y |
| ERBB4 | Receptor tyrosine-protein kinase erbB-4 | Q15303 | Y |
| Erythropoietin | Erythropoietin | P01588 | Y |
| ESAM | Endothelial cell-selective adhesion molecule | Q96AP7 | Y |
| ETHE1 | Protein ETHE1, mitochondrial | O95571 | |
| Factor B | Complement factor B | P00751 | Y |
| Factor D | Complement factor D | P00746 | Y |
| Factor H | Complement factor H | P08603 | Y |
| Factor I | Complement factor I | P05156 | Y |
| Fas ligand, soluble | Tumor necrosis factor ligand superfamily member 6 | P48023 | Y |
| FCγ2A | Low affinity immunoglobulin gamma Fc region receptor II-a | P12318 | Y |
| FCγ2B | Low affinity immunoglobulin gamma Fc region receptor II-b | P31994 | Y |
| FCγ3B | Low affinity immunoglobulin gamma Fc region receptor III-B | O75015 | |
| FCγR1 | High affinity immunoglobulin gamma Fc receptor I | P12314 | Y |
| Ferritin | Ferritin heavy and light chains | P02794, P02792 | Y |
| Fetuin B | Fetuin-B | Q9UGM5 | Y |
| FGF-4 | Fibroblast growth factor 4 | P08620 | |
| FGF-5 | Fibroblast growth factor 5 | P12034 | Y |
| FGF-6 | Fibroblast growth factor 6 | P10767 | Y |
| FGF-7 | Keratinocyte growth factor | P21781 | Y |
| FGF-8B | Fibroblast growth factor 8 - isoform 8B | P55075 | Y |
| FGF-9 | Glia-activating factor | P31371 | Y |
| FGF-10 | Fibroblast growth factor 10 | O15520 | Y |
| FGF-16 | Fibroblast growth factor 16 | O43320 | Y |
| FGF-17 | Fibroblast growth factor 17 | O60258 | Y |
| FGF-18 | Fibroblast growth factor 18 | O76093 | Y |
| FGF-19 | Fibroblast growth factor 19 | O95750 | Y |

| Target | Uni-Prot Protein Name | Uni-Prot Acc # | CKD |
|---|---|---|---|
| FGF-20 | Fibroblast growth factor 20 | Q9NP95 | Y |
| FGF-23 | Fibroblast growth factor 23 | Q9GZV9 | Y |
| FGFR-2 | Fibroblast growth factor receptor 2 | P21802 | |
| FGFR-3 | Fibroblast growth factor receptor 3 | P22607 | |
| FGR | Proto-oncogene tyrosine-protein kinase FGR | P09769 | |
| Fibrinogen | Fibrinogen alpha, beta, and gamma chains | P02671, P02675, P02679 | Y |
| Fibronectin | Fibronectin | P02751 | Y |
| Fibronectin FN1.3 | Fibronectin-1 Fragment 3 | P02751 | Y |
| Fibronectin FN1.4 | Fibronectin-1 Fragment 4 | P02751 | Y |
| Ficolin-1 | Ficolin-1 | O00602 | |
| Ficolin-2 | Ficolin-2 | Q15485 | Y |
| Ficolin-3 | Ficolin-3 | O75636 | |
| Flt-3 | FL cytokine receptor | P36888 | Y |
| Flt-3 ligand | SL cytokine | P49771 | Y |
| Follistatin | Follistatin | P19883 | Y |
| Fortilin | Translationally-controlled tumor protein | P13693 | |
| Fractalkine/CX3CL-1 | Fractalkine | P78423 | Y |
| FRP-1, soluble | Secreted frizzled-related protein 1 | Q8N474 | Y |
| FRP-3, soluble | Secreted frizzled-related protein 3 | Q92765 | Y |
| FSH | Glycoprotein hormones alpha chain and Follitropin subunit beta | P01215, P01225 | Y |
| FSTL3 | Follistatin-related protein 3 | O95633 | Y |
| FYN | Proto-oncogene tyrosine-protein kinase Fyn | P06241 | Y |
| GA733-1 protein | Tumor-associated calcium signal transducer 2 | P09758 | Y |
| Galectin-2 | Galectin-2 | P05162 | Y |
| Galectin-3 | Galectin-3 | P17931 | Y |
| Galectin-4 | Galectin-4 | P56470 | Y |
| GAPDH | Glyceraldehyde-3-phosphate dehydrogenase | P04406 | |
| Gas1 | Growth arrest-specific protein 1 | P54826 | Y |
| GASP-1 | WAP, kazal, immunoglobulin, kunitz and NTR domain-containing protein 2 | Q8TEU8 | Y |
| GASP-2 | G-protein coupled receptor-associated sorting protein 2 | Q96D09 | Y |
| Gastrin-releasing peptide | Gastrin-releasing peptide | P07492 | |
| GCP-2 | C-X-C motif chemokine 6 | P80162 | Y |
| G-CSF-R | Granulocyte colony-stimulating factor receptor | Q99062 | Y |
| GDF-11 | Growth/differentiation factor 11 | O95390 | Y |
| GDF-9 | Growth/differentiation factor 9 | O60383 | Y |
| GDNF | Glial cell line-derived neurotrophic factor | P39905 | Y |
| GFAP | Glial fibrillary acidic protein | P14136 | Y |
| GFR α-1 | GDNF family receptor alpha-1 | P56159 | Y |
| GFR α-2 | GDNF family receptor alpha-2 | O00451 | Y |
| GFR α-3 | GDNF family receptor alpha-3 | O60609 | Y |
| Glucocorticoid receptor | Glucocorticoid receptor | P04150 | Y |
| Glutamate carboxypeptidase | Cytosolic non-specific dipeptidase | Q96KP4 | Y |
| Glycoprotein VI | Platelet glycoprotein VI | Q9HCN6 | Y |
| Glypican 2 | Glypican-2 | Q8N158 | Y |
| Glypican 3 | Glypican-3 | P51654 | Y |
| GNS | N-acetylglucosamine-6-sulfatase | P15586 | |
| gp130, soluble | Interleukin-6 receptor subunit beta | P40189 | Y |
| GPIIbIIIa | Integrin alpha-Iib and Integrin beta-3 | P08514,P05106 | |
| Granulysin | Granulysin | P22749 | |
| Granzyme A | Granzyme A | P12544 | Y |
| Granzyme B | Granzyme B | P10144 | Y |
| Granzyme H | Granzyme H | P20718 | Y |
| Group IB phospholipase A2 | Phospholipase A2 | P04054 | Y |
| Group IIA phospholipase A2 | Phospholipase A2, membrane associated | P14555 | Y |
| Group IIE phospholipase A2 | Group IIE secretory phospholipase A2 | Q9NZK7 | Y |
| Group V phospholipase A2 | Calcium-dependent phospholipase A2 | P39877 | Y |
| Group X phospholipase A2 | Group 10 secretory phospholipase A2 | O15496 | Y |
| Growth hormone receptor | Growth hormone receptor | P10912 | Y |
| Gro-α | Growth-regulated alpha protein | P09341 | Y |
| Gro-β | Macrophage inflammatory protein 2-alpha | P19875 | |

Nature Precedings : hdl:10101/npre.2010.4538.1 : Posted 14 Jun 2010

Nature Precedings : hdl:10101/npre.2010.4538.1 : Posted 14 Jun 2010

| Target | Uni-Prot Protein Name | Uni-Prot Acc # | CKD |
|---|---|---|---|
| Gro-γ | Macrophage inflammatory protein 2-beta | P19876 | Y |
| GSK-3 α | Glycogen synthase kinase-3 alpha | P49840 | Y |
| GSK-3 β | Glycogen synthase kinase-3 beta | P49841 | Y |
| HAI-1 | Kunitz-type protease inhibitor 1 | O43278 | Y |
| HAI-2 | Kunitz-type protease inhibitor 2 | O43291 | |
| HAPLN1 | Hyaluronan and proteoglycan link protein 1 | P10915 | Y |
| Haptoglobin, Mixed Type | Haptoglobin | P00738 | Y |
| Hat1 | Histone acetyltransferase type B catalytic subunit | O14929 | Y |
| HB-EGF | Heparin-binding EGF-like growth factor | Q99075 | Y |
| HCC-1 | C-C motif chemokine 14 | Q16627 | Y |
| HCC-4 | Small-inducible cytokine A16 | O15467 | Y |
| HCK | Tyrosine-protein kinase HCK | P08631 | Y |
| HDAC8 | Histone deacetylase 8 | Q9BY41 | Y |
| HE4 | WAP four-disulfide core domain protein 2 | Q14508 | Y |
| Heme oxygenase 2 | Heme oxygenase 2 | P30519 | Y |
| Hemopexin | Hemopexin | P02790 | Y |
| Heparin cofactor II | Heparin cofactor 2 | P05546 | Y |
| Hepcidin-25 | Hepcidin | P81172 | Y |
| HGF | Hepatocyte growth factor | P14210 | Y |
| HGF activator | Hepatocyte growth factor activator | Q04756 | |
| HGF R | Hepatocyte growth factor receptor | P08581 | Y |
| HINT1 | Histidine triad nucleotide-binding protein 1 | P49773 | |
| HIPK3 | Homeodomain-interacting protein kinase 3 | Q9H422 | Y |
| Histone H1.2 | Histone H1.2 | P16403 | Y |
| Histone H2A.z | Histone H2A.Z | P0C0S5 | |
| HIV-2 Rev | Protein Rev (Human Immunodeficiency Virus) | P18093 | Y |
| HMG-1 | High mobility group protein B1 | P09429 | Y |
| HMTase G9a | Histone-lysine N-methyltransferase, H3 lysine-9 specific 3 | Q96KQ7 | Y |
| HPV E7 Type 16 | Protein E7 (Human Papillomavirus Type 16) | P03129 | Y |
| HPV E7 Type 18 | Protein E7 (Human Papillomavirus Type 18) | P06788 | Y |
| HSP 40 | DnaJ homolog subfamily B member 1 | P25685 | |
| HSP 60 | 60 kDa heat shock protein, mitochondrial | P10809 | Y |
| HSP 70 | Heat shock 70 kDa protein 1 | P08107 | |
| HSP 90α | Heat shock protein HSP 90-alpha | P07900 | Y |
| HSP 90β | Heat shock protein HSP 90-beta | P08238 | Y |
| HTRA2 | Serine protease HTRA2, mitochondrial | O43464 | Y |
| iC3b | Complement C3b, incactivated | P01024 | Y |
| ICOS | Inducible T-cell co-stimulator | Q9Y6W8 | Y |
| IDUA | Alpha-L-iduronidase | P35475 | Y |
| Iduronate 2-sulfatase | Iduronate 2-sulfatase | P22304 | Y |
| IFN-αA | Interferon alpha-2 | P01563 | Y |
| IFN-γ | Interferon gamma | P01579 | Y |
| IFN-γ R1 | Interferon-gamma receptor alpha chain | P15260 | |
| IFN-λ 1 | Interleukin-29 | Q8IU54 | Y |
| IFN-λ 2 | Interleukin-28A | Q8IZJ0 | Y |
| IgE | Immunoglobulin E | | Y |
| IGFBP-1 | Insulin-like growth factor-binding protein 1 | P08833 | Y |
| IGFBP-2 | Insulin-like growth factor-binding protein 2 | P18065 | Y |
| IGFBP-3 | Insulin-like growth factor-binding protein 3 | P17936 | Y |
| IGFBP-4 | Insulin-like growth factor-binding protein 4 | P22692 | Y |
| IGFBP-5 | Insulin-like growth factor-binding protein 5 | P24593 | Y |
| IGFBP-6 | Insulin-like growth factor-binding protein 6 | P24592 | Y |
| IGFBP-7 | Insulin-like growth factor-binding protein 7 | Q16270 | Y |
| IGF-I | Insulin-like growth factor IA and IB | P01343, P05019 | Y |
| IGF-II receptor | Cation-independent mannose-6-phosphate receptor | P11717 | Y |
| IgG | Immunoglobulin G | | |
| IgM | Immunoglobulin M | | Y |
| IL-1β | Interleukin-1 beta | P01584 | Y |
| IL-1 F7 | Interleukin-1 family member 7 | Q9NZH6 | Y |
| IL-1 R AcP | Interleukin-1 receptor accessory protein | Q9NPH3 | Y |
| IL-1 R4 | Interleukin-1 receptor-like 1 | Q01638 | |

Nature Precedings : hdl:10101/npre.2010.4538.1 : Posted 14 Jun 2010

| Target | Uni-Prot Protein Name | Uni-Prot Acc # | CKD |
|---|---|---|---|
| IL-1 Rrp2 | Interleukin-1 receptor-like 2 | Q9HB29 | Y |
| IL-1 sRI | Interleukin-1 receptor type I | P14778 | Y |
| IL-2 | Interleukin-2 | P60568 | Y |
| IL-2 sRα | Interleukin-2 receptor alpha chain | P01589 | |
| IL-2 sRγ | Cytokine receptor common gamma chain | P31785 | Y |
| IL-4 | Interleukin-4 | P05112 | Y |
| IL-4 sR | Interleukin-4 receptor alpha chain | P24394 | Y |
| IL-5 | Interleukin-5 | P05113 | |
| IL-6 | Interleukin-6 | P05231 | Y |
| IL-6 sRα | Interleukin-6 receptor alpha chain | P08887 | Y |
| IL-7 | Interleukin-7 | P13232 | Y |
| IL-7 Rα | Interleukin-7 receptor alpha chain | P16871 | Y |
| IL-8 | Interleukin-8 | P10145 | Y |
| IL-9 | Interleukin-9 | P15248 | Y |
| IL-10 | Interleukin-10 | P22301 | Y |
| IL-10 Rβ | Interleukin-10 receptor beta chain | Q08334 | Y |
| IL-11 | Interleukin-11 | P20809 | Y |
| IL-11 Rα | Interleukin-11 receptor alpha chain | Q14626 | |
| IL-12 | Interleukin-12 subunits alpha and beta | P29459, P29460 | |
| IL-12 Rβ1 | Interleukin-12 receptor beta-1 chain | P42701 | Y |
| IL-12 Rβ2 | Interleukin-12 receptor beta-2 chain | Q99665 | Y |
| IL-13 | Interleukin-13 | P35225 | Y |
| IL-13 Rα1 | Interleukin-13 receptor alpha-1 chain | P78552 | Y |
| IL-15 Rα | Interleukin-15 receptor alpha chain | Q13261 | Y |
| IL-16 | Interleukin-16 | Q14005 | Y |
| IL-17 | Interleukin-17A | Q16552 | Y |
| IL-17B | Interleukin-17B | Q9UHF5 | Y |
| IL-17D | Interleukin-17D | Q8TAD2 | Y |
| IL-17E | Interleukin-25 | Q9H293 | Y |
| IL-17F | Interleukin-17F | Q96PD4 | Y |
| IL-17 RC | Interleukin-17 receptor C | Q8NAC3 | |
| IL-17 RD | Interleukin-17 receptor D | Q8NFM7 | Y |
| IL-17 sR | Interleukin-17 receptor A | Q96F46 | Y |
| IL-18 BPa | Interleukin-18-binding protein | O95998 | Y |
| IL-18 Rα | Interleukin-18 receptor 1 | Q13478 | Y |
| IL-18 Rβ | Interleukin-18 receptor accessory protein | O95256 | Y |
| IL-19 | Interleukin-19 | Q9UHD0 | Y |
| IL-20 | Interleukin-20 | Q9NYY1 | Y |
| IL-22 | Interleukin-22 | Q9GZX6 | Y |
| IL-22 Rα1 | Interleukin-22 receptor subunit alpha-1 | Q8N6P7 | |
| IL-24 | Interleukin-24 | Q13007 | Y |
| IL-27 | Interleukin 27 | Q8NEV9 | Y |
| Importin β1 | Importin subunit beta-1 | Q14974 | |
| ING1 | Inhibitor of growth protein 1 | Q9UK53 | |
| Insulysin | Insulin-degrading enzyme | P14735 | Y |
| Integrin α1β1 | Integrin alpha-1 and Integrin beta-1 | P56199, P05556 | Y |
| IP-10 | Small-inducible cytokine B10 | P02778 | Y |
| IR | Insulin receptor | P06213 | Y |
| I-TAC | Small-inducible cytokine B11 | O14625 | Y |
| ICAM-1, soluble | Intercellular adhesion molecule 1 | P05362 | |
| ICAM-2, soluble | Intercellular adhesion molecule 2 | P13598 | Y |
| ICAM-3, soluble | Intercellular adhesion molecule 3 | P32942 | Y |
| JAM-B | Junctional adhesion molecule B | P57087 | Y |
| JAM-C | Junctional adhesion molecule C | Q9BX67 | Y |
| Kallikrein 4 | Kallikrein-4 | Q9Y5K2 | Y |
| Kallikrein 5 | Kallikrein-5 | Q9Y337 | Y |
| Kallikrein 6 | Kallikrein-6 | Q92876 | Y |
| Kallikrein 7 | Kallikrein-7 | P49862 | Y |
| Kallikrein 8 | Neuropsin | O60259 | Y |
| Kallikrein 11 | Kallikrein-11 | Q9UBX7 | |
| Kallikrein 12 | Kallikrein-12 | Q9UKR0 | Y |

Nature Precedings : hdl:10101/npre.2010.4538.1 : Posted 14 Jun 2010

| Target | Uni-Prot Protein Name | Uni-Prot Acc # | CKD |
|---|---|---|---|
| Kallikrein 13 | Kallikrein-13 | Q9UKR3 | Y |
| Kallikrein 14 | Kallikrein-14 | Q9P0G3 | |
| Kallistatin | Kallistatin | P29622 | Y |
| Karyopherin-α2 | Importin subunit alpha-2 | P52292 | Y |
| Kininogen, HMW, Single Chain | Kininogen-1 (single chain form) | P01042 | Y |
| Kininogen, HMW, Two Chain | Kininogen-1 (two-chain form) | P01042 | Y |
| Kremen2 | Kremen protein 2 | Q8NCW0 | Y |
| Ku70 | ATP-dependent DNA helicase 2 subunit 1 | P12956 | Y |
| Lactoferrin | Lactotransferrin | P02788 | Y |
| LAG-1 | Macrophage inflammatory protein-1b2 | Q8NHW4 | Y |
| Lamin-B1 | Lamin-B1 | P20700 | |
| Laminin | Laminin subunits alpha-1, beta-1, and gamma-1 | P25391, P07942, P11047 | Y |
| Langerin | C-type lectin domain family 4 member K | Q9UJ71 | Y |
| Layilin | Layilin | Q6UX15 | Y |
| LBP | Lipopolysaccharide-binding protein | P18428 | Y |
| LCK | Proto-oncogene tyrosine-protein kinase LCK | P06239 | Y |
| LD78-β | Small-inducible cytokine A3-like 1 | P16619 | Y |
| LDH-H 1 | L-lactate dehydrogenase B chain | P07195 | |
| Legumain | Legumain | Q99538 | |
| Leptin | Leptin | P41159 | Y |
| Lipocalin 2 | Neutrophil gelatinase-associated lipocalin | P80188 | Y |
| LOX-1 | Oxidized low-density lipoprotein receptor 1 | P78380 | Y |
| LRIG3 | Leucine-rich repeats and immunoglobulin-like domains protein 3 | Q6UXM1 | Y |
| LRPAP | Alpha-2-macroglobulin receptor-associated protein | P30533 | Y |
| LSAMP | Limbic system-associated membrane protein | Q13449 | Y |
| LTA-4 hydrolase | Leukotriene A-4 hydrolase | P09960 | |
| Luteinizing hormone | Glycoprotein hormones alpha chain and Lutropin subunit beta | P01215, P01229 | Y |
| LY9 | T-lymphocyte surface antigen Ly-9 | Q9HBG7 | Y |
| Lymphotactin | Lymphotactin | P47992 | Y |
| Lymphotoxin α1/β2 | Lymphotoxin-alpha (1) and Lymphotoxin-beta (2) | P01374, Q06643 | Y |
| Lymphotoxin α2/β1 | Lymphotoxin-alpha (2) and Lymphotoxin-beta (1) | P01374, Q06643 | Y |
| Lymphotoxin β R | Tumor necrosis factor receptor superfamily member 3 | P36941 | Y |
| LYN A | Tyrosine-protein kinase Lyn | P07948 | Y |
| LYN B | Tyrosine-protein kinase Lyn, isoform B | P07948-2 | Y |
| Lysozyme | Lysozyme C | P61626 | Y |
| LYVE-1 | Lymphatic vessel endothelial hyaluronic acid receptor 1 | Q9Y5Y7 | Y |
| Macrophage mannose receptor | Macrophage mannose receptor 1 | P22897 | Y |
| Macrophage scavenger receptor | Macrophage scavenger receptor types I and II | P21757 | |
| MAP2K2 | Dual specificity mitogen-activated protein kinase kinase 2 | P36507 | |
| MAPK1 | Mitogen-activated protein kinase 1 | P28482 | Y |
| MAPK12 | Mitogen-activated protein kinase 12 | P53778 | |
| MAPK13 | Mitogen-activated protein kinase 13 | O15264 | Y |
| MAPK14 | Mitogen-activated protein kinase 14 | Q16539 | Y |
| MAPK3 | Mitogen-activated protein kinase 3 | P27361 | Y |
| MAPK8 | Mitogen-activated protein kinase 8 | P45983 | |
| MAPKAPK2 | MAP kinase-activated protein kinase 2 | P49137 | |
| MAPKAPK3 | MAP kinase-activated protein kinase 3 | Q16644 | |
| MAPKAPK5 | MAP kinase-activated protein kinase 5 | Q8IW41 | |
| Marapsin | Serine protease 27 | Q9BQR3 | |
| MASP3 | Complement-activating component of Ra-reactive factor splice variant MASP3 | Q96RS4 | |
| MATK | Megakaryocyte-associated tyrosine-protein kinase | P42679 | Y |
| Matrilin-2 | Matrilin-2 | O00339 | Y |
| Matrilin-3 | Matrilin-3 | O15232 | Y |
| MBD4 | Methyl-CpG-binding domain protein 4 | O95243 | |
| MBL | Mannose-binding protein C | P11226 | Y |
| MCM2 | DNA replication licensing factor MCM2 | P49736 | |
| MCP-1 | Small-inducible cytokine A2 | P13500 | Y |
| MCP-2 | Small-inducible cytokine A8 | P80075 | Y |
| MCP-3 | Small-inducible cytokine A7 | P80098 | Y |
| MCP-4 | Small-inducible cytokine A13 | Q99616 | Y |

Nature Precedings : hdl:10101/npre.2010.4538.1 : Posted 14 Jun 2010

| Target | Uni-Prot Protein Name | Uni-Prot Acc # | CKD |
|---|---|---|---|
| M-CSF | Macrophage colony-stimulating factor 1 | P09603 | |
| M-CSF R | Macrophage colony-stimulating factor 1 receptor | P07333 | Y |
| MD-1 | Lymphocyte antigen 86 | O95711 | Y |
| MDC | Small-inducible cytokine A22 | O00626 | Y |
| MDHC | Malate dehydrogenase, cytoplasmic | P40925 | |
| Mediator complex subunit 1 | Mediator of RNA polymerase II transcription subunit 1 | Q15648 | |
| MEK1 | Dual specificity mitogen-activated protein kinase kinase 1 | Q02750 | Y |
| MEPE | Matrix extracellular phosphoglycoprotein | Q9NQ76 | Y |
| Mesothelin | Mesothelin | Q13421 | |
| MetAP 1 | Methionine aminopeptidase 1 | P53582 | Y |
| MetAP2 | Methionine aminopeptidase 2 | P50579 | |
| MFRP | Membrane frizzled-related protein | Q9BY79 | |
| MIA | Melanoma-derived growth regulatory protein | Q16674 | Y |
| MICA | MHC class I chain-related protein A | Q29983 | Y |
| Midkine | Midkine | P21741 | Y |
| MIG | Small-inducible cytokine B9 | Q07325 | Y |
| MIP-1α | C-C motif chemokine 3 | P10147 | Y |
| MIP-1β | Small-inducible cytokine A4 | P13236 | Y |
| MIP-3α | Small-inducible cytokine A20 | P78556 | Y |
| MIP-3β | Small-inducible cytokine A19 | Q99731 | Y |
| MIP-4 | C-C motif chemokine 18 | P55774 | Y |
| MIP-5 | C-C motif chemokine 15 | Q16663 | Y |
| MMP-2 | 72 kDa type IV collagenase | P08253 | |
| MMP-3 | Stromelysin-1 | P08254 | |
| MMP-7 | Matrilysin | P09237 | Y |
| MMP-8 | Neutrophil collagenase | P22894 | Y |
| MMP-9 | Matrix metalloproteinase-9 | P14780 | Y |
| MMP-10 | Stromelysin-2 | P09238 | Y |
| MMP-17 | Matrix metalloproteinase-17 | Q9ULZ9 | |
| MOZ | Histone acetyltransferase MYST3 | Q92794 | Y |
| MPIF-1 | C-C motif chemokine 23 | P55773 | Y |
| MRC2 | Macrophage mannose receptor 2 | Q9UBG0 | Y |
| MRCKβ | Serine/threonine-protein kinase MRCK beta | Q9Y5S2 | |
| MSP R | Macrophage-stimulating protein receptor | Q04912 | Y |
| Myeloperoxidase | Myeloperoxidase | P05164 | Y |
| Myoglobin | Myoglobin | P02144 | Y |
| Myosin regulatory light chain 2 | Myosin regulatory light chain 2, ventricular/cardiac muscle isoform | P10916 | Y |
| NACα | Nascent polypeptide-associated complex subunit alpha | Q13765 | |
| NADPH-P450 Oxidoreductase | NADPH--cytochrome P450 reductase | P16435 | Y |
| NAGK | N-acetyl-D-glucosamine kinase | Q9UJ70 | |
| NANOG | Homeobox protein NANOG | Q9H9S0 | Y |
| NAP-2 | Neutrophil-activating peptide 2 | P02775 | Y |
| Nectin-like protein 1 | Cell adhesion molecule 3 | Q8N126 | |
| Nectin-like protein 2 | Cell adhesion molecule 1 | Q9BY67 | Y |
| Neprilysin-2 | Membrane metallo-endopeptidase-like 1 | Q495T6 | |
| Netrin-4 | Netrin-4 | Q9HB63 | Y |
| NEUREGULIN-1 | Neuregulin-1 | Q02297 | Y |
| Neurotrophin-3 | Neurotrophin-3 | P20783 | Y |
| Neurotrophin-5 | Neurotrophin-5 | P34130 | Y |
| Nidogen | Nidogen-1 | P14543 | Y |
| Nidogen-2 | Nidogen-2 | Q14112 | |
| NKG2D | NKG2-D type II integral membrane protein | P26718 | Y |
| NKp30 | Natural cytotoxicity triggering receptor 3 | O14931 | Y |
| NKp44 | Natural cytotoxicity triggering receptor 2 | O95944 | Y |
| Noggin | Noggin | Q13253 | Y |
| Nogo Receptor | Reticulon-4 receptor | Q9BZR6 | Y |
| NovH | Protein NOV homolog | P48745 | Y |
| NRP1 | Neuropilin-1 | O14786 | Y |
| OBCAM | Opioid-binding protein/cell adhesion molecule | Q14982 | |
| OCIAD1 | OCIA domain-containing protein 1 | Q9NX40 | |
| Oncostatin M | Oncostatin-M | P13725 | Y |

| Target | Uni-Prot Protein Name | Uni-Prot Acc # | CKD |
|---|---|---|---|
| RUNX-2 | Runt-related transcription factor 2 | Q13950 | Y |
| Osteonectin | SPARC | P09486 | Y |
| Osteoprotegerin | Tumor necrosis factor receptor superfamily member 11B | O00300 | Y |
| Otubain-1 | Ubiquitin thioesterase OTUB1 | Q96FW1 | |
| OX40 Ligand | Tumor necrosis factor ligand superfamily member 4 | P23510 | Y |
| p27Kip1 | Cyclin-dependent kinase inhibitor 1B | P46527 | |
| PAFAH β subunit | Platelet-activating factor acetylhydrolase IB subunit beta | P68402 | Y |
| PAI-1 | Plasminogen activator inhibitor 1 | P05121 | Y |
| PAK3 | Serine/threonine-protein kinase PAK 3 | O75914 | Y |
| PAK6 | Serine/threonine-protein kinase PAK 6 | Q9NQU5 | |
| PAK7 | Serine/threonine-protein kinase PAK 7 | Q9P286 | Y |
| PAPP-A | Pappalysin-1 | Q13219 | Y |
| P-Cadherin | Cadherin-3 | P22223 | Y |
| PCNA | Proliferating cell nuclear antigen | P12004 | Y |
| PDGF Rβ | Beta-type platelet-derived growth factor receptor | P09619 | Y |
| PDGF-AA | Platelet-derived growth factor A chain | P04085 | Y |
| PDGF-BB | Platelet-derived growth factor B chain | P01127 | Y |
| PDGF-CC | Platelet-derived growth factor C chain | Q9NRA1 | |
| PD-L2 | Programmed cell death 1 ligand 2 | Q9BQ51 | Y |
| PDPK1 | 3-phosphoinositide-dependent protein kinase 1 | O15530 | Y |
| PECAM-1 | Platelet endothelial cell adhesion molecule | P16284 | Y |
| Peptide YY | Peptide YY | P10082 | |
| Peroxiredoxin-1 | Peroxiredoxin-1 | Q06830 | |
| Persephin | Persephin | O60542 | Y |
| PF-4 | Platelet factor 4 | P02776 | Y |
| PGRP-S | Peptidoglycan recognition protein | O75594 | Y |
| Phosphoglycerate mutase 1 | Phosphoglycerate mutase 1 | P18669 | |
| pIgR | Polymeric immunoglobulin receptor | P01833 | Y |
| PIK3Cα/PIK3R1 | Phosphatidylinositol-4,5-bisphosphate 3-kinase catalytic subunit alpha isoform, Phosphatidylinositol 3-kinase regulatory subunit alpha Complex | P42336, P27986 | Y |
| PK3CG | Phosphatidylinositol-4,5-bisphosphate 3-kinase catalytic subunit gamma isoform | P48736 | Y |
| PKB | RAC-alpha serine/threonine-protein kinase | P31749 | Y |
| PKB γ | RAC-gamma serine/threonine-protein kinase | Q9Y243 | Y |
| PKC-α | Protein kinase C alpha type | P17252 | Y |
| PKC-β-II | Protein kinase C beta type (splice variant Beta-II) | P05771 | Y |
| PKC-γ | Protein kinase C gamma type | P05129 | |
| PKC-δ | Protein kinase C delta type | Q05655 | Y |
| PKC-ζ | Protein kinase C zeta type | Q05513 | Y |
| Plasmin | Plasmin heavy chain A and light chain B | P00747 | Y |
| Plasminogen | Plasminogen | P00747 | Y |
| Pleiotrophin | Pleiotrophin | P21246 | Y |
| PLGF | Placenta growth factor | P49763 | Y |
| PLK-1 | Serine/threonine-protein kinase PLK1 | P53350 | Y |
| PLPP | Pyridoxal phosphate phosphatase | Q96GD0 | |
| PPAC | Low molecular weight phosphotyrosine protein phosphatase | P24666 | |
| Prekallikrein | Plasma kallikrein (precursor) | P03952 | Y |
| PRKA C-α | cAMP-dependent protein kinase catalytic subunit alpha | P17612 | Y |
| PRKCI | Protein kinase C iota type | P41743 | Y |
| PRKCQ | Protein kinase C theta type | Q04759 | |
| PRL | Prolactin | P01236 | Y |
| Properdin | Properdin | P27918 | Y |
| Protease nexin I | Glia-derived nexin | P07093 | Y |
| Proteasome subunit p40 | 26S proteasome non-ATPase regulatory subunit 7 | P51665 | |
| Proteasome subunit α1 | Proteasome subunit alpha type-1 | P25786 | |
| Proteasome subunit α6 | Proteasome subunit alpha type-6 | P60900 | |
| Protein C | Vitamin K-dependent protein C | P04070 | Y |
| Protein C Inhibitor | Plasma serine protease inhibitor | P05154 | Y |
| Protein S | Vitamin K-dependent protein S | P07225 | Y |
| Proteinase-3 | Myeloblastin | P24158 | Y |

Nature Precedings : hdl:10101/npre.2010.4538.1 : Posted 14 Jun 2010

| Target | Uni-Prot Protein Name | Uni-Prot Acc # | CKD |
|---|---|---|---|
| Prothrombin | Prothrombin | P00734 | Y |
| PSA | Prostate-specific antigen | P07288 | Y |
| PSA-ACT | Prostate-specific antigen and Alpha-1-antichymotrypsin | P07288, P01011 | Y |
| P-Selectin | P-selectin | P16109 | Y |
| PSMA | Glutamate carboxypeptidase 2 | Q04609 | |
| pTEN | Phosphatidylinositol-3,4,5-trisphosphate 3-phosphatase and dual-specificity protein phosphatase PTEN | P60484 | Y |
| PTH | Parathyroid hormone | P01270 | |
| PTHrP | Parathyroid hormone-related protein | P12272 | Y |
| PTK6 | Tyrosine-protein kinase 6 | Q13882 | |
| PTP-1B | Tyrosine-protein phosphatase non-receptor type 1 | P18031 | Y |
| Rab GDP dissociation inhibitor β | Rab GDP dissociation inhibitor beta | P50395 | Y |
| RAC1 | Ras-related C3 botulinum toxin substrate 1 | P63000 | Y |
| RACK1 | Guanine nucleotide-binding protein subunit beta-2-like 1 | P63244 | |
| RAD51 | DNA repair protein RAD51 homolog 1 | Q06609 | Y |
| RANTES | Small-inducible cytokine A5 | P13501 | Y |
| RBP | Retinol-binding protein 4 | P02753 | |
| RELT | Tumor necrosis factor receptor superfamily member 19L | Q969Z4 | Y |
| Renin | Renin | P00797 | Y |
| resistin | Resistin | Q9HD89 | Y |
| RET | Proto-oncogene tyrosine-protein kinase receptor ret | P07949 | Y |
| RGM-A | Repulsive guidance molecule A | Q96B86 | |
| RGM-B | RGM domain family member B | Q6NW40 | |
| RGM-C | Hemojuvelin | Q6ZVN8 | Y |
| RNAbp 39 | RNA-binding protein 39 | Q14498 | |
| ROR1 | Tyrosine-protein kinase transmembrane receptor ROR1 | Q01973 | Y |
| RPS6Kα3 | Ribosomal protein S6 kinase alpha-3 | P51812 | Y |
| RS3A | 40S ribosomal protein S3a | P61247 | |
| RS7 | 40S ribosomal protein S7 | P62081 | |
| RSK-like protein kinase | Ribosomal protein S6 kinase alpha-5 | O75582 | |
| S100A12 | Protein S100-A12 | P80511 | |
| SAA | Serum amyloid A protein | P02735 | |
| SAP | Serum amyloid P-component | P02743 | Y |
| SBDS | Ribosome maturation protein SBDS | Q9Y3A5 | |
| SCF sR | Mast/stem cell growth factor receptor | P10721 | Y |
| SCGF-α | C-type lectin domain family 11 member A (alpha form) | Q9Y240 | Y |
| SCGF-β | C-type lectin domain family 11 member A (beta form) | Q9Y240 | Y |
| SDF-1α | SDF-1-alpha | P48061 | Y |
| SDF-1β | SDF-1-beta | P48061 | Y |
| Secretin | Secretin | P09683 | |
| Semaphorin 3A | Semaphorin-3A | Q14563 | |
| sE-Selectin | E-selectin | P16581 | Y |
| SET9 | Histone-lysine N-methyltransferase, H3 lysine-4 specific SET7 | Q8WTS6 | Y |
| SEZ6L2 | Seizure 6-like protein 2 | Q6UXD5 | |
| SGTα | Small glutamine-rich tetratricopeptide repeat-containing protein alpha | O43765 | |
| SHP-2 | Tyrosine-protein phosphatase non-receptor type 11 | Q06124 | Y |
| Siglec-3 | Myeloid cell surface antigen CD33 | P20138 | |
| Siglec-6 | Sialic acid-binding Ig-like lectin 6 | O43699 | Y |
| Siglec-7 | Sialic acid-binding Ig-like lectin 7 | Q9Y286 | Y |
| Siglec-9 | Sialic acid-binding Ig-like lectin 9 | Q9Y336 | Y |
| SKP1 | S-phase kinase-associated protein 1 | P63208 | |
| SLAMF5 | SLAM family member 5 | Q9UIB8 | |
| SLITRK1 | SLIT and NTRK-like protein 1 | Q96PX8 | |
| SLPI | Antileukoproteinase | P03973 | Y |
| sL-Selectin | L-selectin | P14151 | Y |
| SMAC/Diablo | Diablo homolog, mitochondrial | Q9NR28 | Y |
| SOD1 | Superoxide dismutase [Cu-Zn] | P00441 | Y |
| Soggy-1 | Dickkopf-like protein 1 | Q9UK85 | Y |
| Somatostatin-28 | Somatostatin-28 | P61278 | |
| Sonic Hedgehog | Sonic hedgehog protein | Q15465 | Y |

Nature Precedings : hdl:10101/npre.2010.4538.1 : Posted 14 Jun 2010

| Target | Uni-Prot Protein Name | Uni-Prot Acc # | CKD |
|---|---|---|---|
| Sorting nexin 4 | Sorting nexin-4 | O95219 | |
| sRAGE | Advanced glycosylation end product-specific receptor | Q15109 | |
| sRANKL | Tumor necrosis factor ligand superfamily member 11 | O14788 | Y |
| SRCN1 | V-src sarcoma (Schmidt-Ruppin A-2) viral oncogene homolog (Avian) | Q76P87 | |
| Stabilin-2 | Stabilin-2 | Q8WWQ8 | Y |
| sTie-1 | Tyrosine-protein kinase receptor Tie-1 | P35590 | Y |
| sTie-2 | Angiopoietin-1 receptor | Q02763 | Y |
| STK16 | Serine/threonine-protein kinase 16 | O75716 | Y |
| Stress-induced-phosphoprotein 1 | Stress-induced-phosphoprotein 1 | P31948 | |
| suPAR | Urokinase plasminogen activator surface receptor | Q03405 | Y |
| Survivin | Baculoviral IAP repeat-containing protein 5 | O15392 | Y |
| Syntaxin 1A | Syntaxin-1A | Q16623 | Y |
| TACI | Tumor necrosis factor receptor superfamily member 13B | O14836 | Y |
| TAFI | Carboxypeptidase B2 | Q96IY4 | |
| TARC | Small-inducible cytokine A17 | Q92583 | |
| tau | Microtubule-associated protein tau | P10636 | Y |
| TBK1 | Serine/threonine-protein kinase TBK1 | Q9UHD2 | Y |
| TBP | TATA-box-binding protein | P20226 | Y |
| TCPTP | Tyrosine-protein phosphatase non-receptor type 2 | P17706 | Y |
| TEC | Tyrosine-protein kinase Tec | P42680 | |
| TECK | Small-inducible cytokine A25 | O15444 | Y |
| Tenascin | Tenascin | P24821 | Y |
| Testican-1 | Testican-1 | Q08629 | |
| Testican-2 | Testican-2 | Q92563 | Y |
| TFPI | Tissue factor pathway inhibitor | P10646 | Y |
| TGF-β1 | Transforming growth factor beta-1 | P01137 | Y |
| TGF-β2 | Transforming growth factor beta-2 | P61812 | Y |
| TGF-β3 | Transforming growth factor beta-3 | P10600 | Y |
| TGF-β R III | TGF-beta receptor type III | Q03167 | Y |
| Thrombin | Thrombin heavy and light chains | P00734 | Y |
| Thrombopoietin | Thrombopoietin | P40225 | Y |
| Thrombopoietin Receptor | Thrombopoietin receptor | P40238 | |
| Thrombospondin-1 | Thrombospondin-1 | P07996 | Y |
| Thrombospondin-2 | Thrombospondin-2 | P35442 | |
| Thrombospondin-4 | Thrombospondin-4 | P35443 | Y |
| Thyroglobulin | Thyroglobulin | P01266 | |
| Thyroid peroxidase | Thyroid peroxidase | P07202 | |
| Thyroxine-Binding Globulin | Thyroxine-binding globulin | P05543 | Y |
| TIMP-1 | Metalloproteinase inhibitor 1 | P01033 | Y |
| TIMP-2 | Metalloproteinase inhibitor 2 | P16035 | Y |
| TIMP-3 | Metalloproteinase inhibitor 3 | P35625 | Y |
| TLR2 | Toll-like receptor 2 | O60603 | |
| TLR4 | Toll-like receptor 4 | O00206 | |
| TNF sR-I | Tumor necrosis factor receptor superfamily member 1A | P19438 | Y |
| TNF sR-II | Tumor necrosis factor receptor superfamily member 1B | P20333 | Y |
| TNFSF15 | Tumor necrosis factor ligand superfamily member 15 | O95150 | Y |
| TNFSF18 | Tumor necrosis factor ligand superfamily member 18 | Q9UNG2 | Y |
| TNR4 | Tumor necrosis factor receptor superfamily member 4 | P43489 | |
| Topoisomerase I | DNA topoisomerase 1 | P11387 | Y |
| tPA | Tissue-type plasminogen activator | P00750 | Y |
| TRAIL R4 | Tumor necrosis factor receptor superfamily member 10D | Q9UBN6 | Y |
| Transferrin | Serotransferrin | P02787 | |
| TrATPase | Tartrate-resistant acid phosphatase type 5 | P13686 | Y |
| TrkA | High affinity nerve growth factor receptor | P04629 | Y |
| TrkC | NT-3 growth factor receptor | Q16288 | Y |
| Troponin I | Troponin I, cardiac muscle | P19429 | Y |
| Troponin T | Troponin T, cardiac muscle | P45379 | Y |
| Trypsin | Trypsin-1 | P07477 | Y |
| Trypsin 3 | Trypsin-3 | P35030 | Y |
| Tryptase β-2 | Tryptase beta-2 | P20231 | Y |

Nature Precedings : hdl:10101/npre.2010.4538.1 : Posted 14 Jun 2010

| Target | Uni-Prot Protein Name | Uni-Prot Acc # | CKD |
|---|---|---|---|
| Tryptase γ | Tryptase gamma | Q9NRR2 | Y |
| TSH | Glycoprotein hormones alpha and Thyrotropin subunit beta chains | P01215, P01222 | Y |
| TSLP | Thymic stromal lymphopoietin | Q969D9 | Y |
| TSLP R | Thymic stromal lymphopoietin protein receptor | Q9HC73 | Y |
| TWEAK | Tumor necrosis factor ligand superfamily member 12 | O43508 | Y |
| UB2L3 | Ubiquitin-conjugating enzyme E2 L3 | P68036 | |
| UBC9 | SUMO-conjugating enzyme UBC9 | P63279 | Y |
| UBE2N | Ubiquitin-conjugating enzyme E2 N | P61088 | |
| Ubiquitin+1 | Ubiquitin | P62988 | Y |
| UFC1 | Ufm1-conjugating enzyme 1 | Q9Y3C8 | Y |
| UFM1 | Ubiquitin-fold modifier 1 | P61960 | |
| ULBP-1 | NKG2D ligand 1 | Q9BZM6 | Y |
| ULBP-2 | NKG2D ligand 2 | Q9BZM5 | Y |
| ULBP-3 | NKG2D ligand 3 | Q9BZM4 | Y |
| uPA | Urokinase-type plasminogen activator | P00749 | Y |
| URB | Coiled-coil domain-containing protein 80 | Q76M96 | Y |
| Vasoactive Intestinal Peptide | Vasoactive intestinal peptide | P01282 | |
| VCAM-1 | Vascular cell adhesion protein 1 | P19320 | Y |
| VEGF | Vascular endothelial growth factor A | P15692 | Y |
| VEGF-C | Vascular endothelial growth factor C | P49767 | Y |
| VEGF-D | Vascular endothelial growth factor D | O43915 | Y |
| VEGF sR2 | Vascular endothelial growth factor receptor 2 | P35968 | Y |
| VEGF sR3 | Vascular endothelial growth factor receptor 3 | P35916 | Y |
| VHR | Dual specificity protein phosphatase 3 | P51452 | Y |
| vWF | von Willebrand factor | P04275 | Y |
| WIF-1 | Wnt inhibitory factor 1 | Q9Y5W5 | Y |
| WISP-1 | WNT1-inducible-signaling pathway protein 1 | O95388 | Y |
| WISP-3 | WNT1-inducible-signaling pathway protein 3 | O95389 | Y |
| WNK3 | Serine/threonine-protein kinase WNK3 | Q9BYP7 | Y |
| XEDAR | Tumor necrosis factor receptor superfamily member 27 | Q9HAV5 | Y |
| X-Pro aminopeptidase 1 | Xaa-Pro aminopeptidase 1 | Q9NQW7 | Y |
| YES | Proto-oncogene tyrosine-protein kinase Yes | P07947 | Y |
| YKL-40 | Chitinase-3-like protein 1 | P36222 | |
| ZAP70 | Tyrosine-protein kinase ZAP-70 | P43403 | Y |

Nature Precedings : hdl:10101/npre.2010.4538.1 : Posted 14 Jun 2010

Table 5. Limits of quantification

| Target | Concentration (M) ($\sigma_{logRFU}$ Fit) | | |
|---|---|---|---|
| | LLOQ | ULOQ | log Range |
| α1-Antichymotrypsin | 2.4E-13 | 9.1E-10 | 3.6 |
| α1-Antitrypsin | 1.6E-11 | 7.6E-08 | 3.7 |
| α2-Antiplasmin | 6.0E-13 | 5.0E-08 | 4.9 |
| α2-HS-Glycoprotein | 4.1E-13 | 1.8E-08 | 4.6 |
| α2-Macroglobulin | 3.5E-12 | 3.9E-08 | 4.0 |
| Activated Protein C | 7.3E-13 | 2.6E-09 | 3.6 |
| Activin A | 3.5E-13 | 4.2E-09 | 4.1 |
| ADAMTS-4 | 4.8E-13 | 2.7E-10 | 2.8 |
| Aggrecan | 1.2E-12 | 2.0E-09 | 3.2 |
| AIF-1 | 2.5E-12 | 8.1E-10 | 2.5 |
| Albumin | 4.9E-11 | 2.0E-08 | 2.6 |
| Alkaline phosphatase, bone | 8.0E-12 | 1.2E-08 | 3.2 |
| ALT | 4.3E-12 | 6.9E-09 | 3.2 |
| amyloid precursor protein | 2.6E-13 | 7.6E-10 | 3.5 |
| Angiogenin | 6.6E-13 | 1.1E-09 | 3.2 |
| Angiopoietin-1 | 5.2E-11 | 6.9E-09 | 2.1 |
| Angiopoietin-2 | 1.8E-13 | 1.2E-09 | 3.8 |
| Angiopoietin-4 | 8.5E-13 | 2.9E-09 | 3.5 |
| Angiostatin | 1.5E-13 | 2.4E-09 | 4.2 |
| Angiotensinogen | 4.7E-12 | 5.5E-08 | 4.1 |
| Apo A-I | 2.0E-11 | 2.5E-08 | 3.1 |
| Apo B | 1.1E-12 | 4.5E-09 | 3.6 |
| Apo E | 3.5E-11 | 7.8E-09 | 2.4 |
| Apo E2 | 1.8E-10 | 6.8E-09 | 1.6 |
| Apo E3 | 1.9E-12 | 6.5E-10 | 2.5 |
| Apo E4 | 9.1E-13 | 1.3E-09 | 3.2 |
| APRIL | 1.3E-10 | 5.1E-09 | 1.6 |
| β2-Microglobulin | 5.9E-13 | 1.8E-09 | 3.5 |
| B7 | 1.9E-11 | 3.4E-08 | 3.3 |
| BARK1 | 1.3E-13 | 5.5E-10 | 3.6 |
| BCA-1 | 2.0E-13 | 2.7E-10 | 3.1 |
| Bcl-2 | 1.0E-12 | 2.4E-09 | 3.4 |
| BCL2A1 | 2.4E-13 | 7.8E-10 | 3.5 |
| BDNF | 1.4E-11 | 1.1E-08 | 2.9 |
| βIGH3 | 1.5E-12 | 1.3E-09 | 2.9 |
| BMP-1 | 9.1E-13 | 1.1E-09 | 3.1 |
| BMP10 | 1.3E-10 | 3.4E-08 | 2.4 |
| BMP-14 | 1.3E-12 | 2.4E-09 | 3.3 |
| BMP-6 | 2.2E-12 | 7.5E-10 | 2.5 |
| BMP-7 | 1.5E-12 | 1.3E-09 | 3.0 |
| BMPR1A | 1.6E-11 | 2.4E-08 | 3.2 |
| Bone proteoglycan II | 7.3E-12 | 1.6E-10 | 1.3 |
| BPI | 2.6E-13 | 4.6E-10 | 3.3 |
| BTK | 1.6E-13 | 2.0E-09 | 4.1 |
| C1q | 8.2E-13 | 2.9E-10 | 2.6 |
| C1r | 1.3E-13 | 1.6E-09 | 4.1 |
| C1s | 6.7E-12 | 1.6E-08 | 3.4 |
| C2 | 5.4E-14 | 6.0E-10 | 4.0 |
| C3 | 6.5E-13 | 8.4E-10 | 3.1 |
| C3a | 7.0E-11 | 2.4E-08 | 2.5 |
| C3adesArg | 4.2E-14 | 4.4E-10 | 4.0 |

Nature Precedings : hdl:10101/npre.2010.4538.1 : Posted 14 Jun 2010

| Target | Concentration (M) ($\sigma_{logRFU}$ Fit) | | |
|---|---|---|---|
| | LLOQ | ULOQ | log Range |
| C3b | 6.5E-11 | 4.9E-09 | 1.9 |
| C4 | 6.0E-14 | 4.6E-09 | 4.9 |
| C4b | 7.6E-12 | 7.8E-09 | 3.0 |
| C5 | 4.3E-12 | 2.7E-09 | 2.8 |
| C5a | 3.4E-13 | 1.3E-09 | 3.6 |
| C5b,6 Complex | 2.4E-12 | 9.0E-10 | 2.6 |
| C6 | 1.4E-12 | 4.6E-10 | 2.5 |
| C7 | 5.6E-12 | 7.9E-09 | 3.2 |
| C8 | 2.0E-11 | 4.2E-09 | 2.3 |
| C9 | 2.6E-14 | 3.6E-10 | 4.1 |
| Cadherin-1 | 1.8E-12 | 7.2E-09 | 3.6 |
| Cadherin-12 | 4.3E-10 | 1.0E-06 | 3.4 |
| Cadherin-2 | 4.0E-11 | 3.4E-08 | 2.9 |
| Cadherin-5 | 3.9E-12 | 7.0E-09 | 3.3 |
| Cadherin-6 | 2.3E-12 | 6.8E-09 | 3.5 |
| Calpain I | 7.0E-14 | 3.3E-10 | 3.7 |
| CAMK1D | 7.9E-11 | 6.1E-10 | 0.9 |
| Carbonic anhydrase VI | 5.6E-11 | 1.4E-08 | 2.4 |
| Carbonic anhydrase IX | 1.0E-06 | 1.0E-06 | 0.0 |
| Cardiotrophin-1 | 1.4E-12 | 1.1E-09 | 2.9 |
| Caspase-3 | 4.2E-13 | 3.2E-09 | 3.9 |
| Catalase | 7.8E-14 | 8.2E-10 | 4.0 |
| Cathepsin A | 5.4E-14 | 1.2E-09 | 4.3 |
| Cathepsin B | 5.8E-13 | 5.4E-10 | 3.0 |
| Cathepsin G | 5.9E-12 | 1.8E-09 | 2.5 |
| Cathepsin H | 8.0E-13 | 2.1E-09 | 3.4 |
| Cathepsin S | 2.5E-12 | 1.5E-09 | 2.8 |
| Cathepsin V | 8.7E-13 | 6.5E-10 | 2.9 |
| CCL28 | 8.8E-13 | 3.6E-10 | 2.6 |
| CD109 | 2.1E-14 | 8.2E-10 | 4.6 |
| CD23 | 2.0E-13 | 2.4E-10 | 3.1 |
| CD30 | 6.9E-13 | 1.7E-09 | 3.4 |
| CD30 Ligand | 6.7E-13 | 1.2E-09 | 3.2 |
| CD48 | 3.4E-13 | 8.0E-10 | 3.4 |
| CD5L | 2.1E-14 | 2.1E-10 | 4.0 |
| CD70 | 1.1E-10 | 1.3E-08 | 2.1 |
| CDK1/cyclin B | 1.7E-12 | 3.0E-09 | 3.3 |
| Chemerin | 2.2E-13 | 4.4E-10 | 3.3 |
| CHL1 | 1.9E-13 | 3.4E-08 | 5.3 |
| Chordin-Like 1 | 2.3E-12 | 6.5E-10 | 2.5 |
| CNTF | 1.1E-13 | 5.7E-10 | 3.7 |
| Coagulation Factor IX | 2.4E-13 | 2.8E-09 | 4.1 |
| Coagulation Factor IXab | 1.4E-13 | 1.0E-09 | 3.9 |
| Coagulation Factor V | 9.0E-13 | 4.8E-10 | 2.7 |
| Coagulation Factor VII | 2.1E-12 | 2.1E-09 | 3.0 |
| Coagulation Factor X | 2.4E-11 | 1.4E-09 | 1.8 |
| Coagulation Factor Xa | 4.3E-13 | 2.0E-10 | 2.7 |
| Coagulation Factor XI | 3.8E-13 | 1.9E-09 | 3.7 |
| complement factor H-related 5 | 5.2E-13 | 2.4E-10 | 2.7 |
| contactin-1 | 7.5E-14 | 5.3E-10 | 3.9 |
| Contactin-4 | 8.3E-13 | 1.2E-09 | 3.2 |
| Cryptic | 8.8E-13 | 1.4E-09 | 3.2 |

Nature Precedings : hdl:10101/npre.2010.4538.1 : Posted 14 Jun 2010

| Target | Concentration (M) ($\sigma_{logRFU}$ Fit) | | |
|---|---|---|---|
| | LLOQ | ULOQ | log Range |
| CSK | 5.3E-13 | 7.7E-10 | 3.2 |
| CTACK | 2.4E-12 | 1.2E-08 | 3.7 |
| CTLA-4 | 6.9E-13 | 1.1E-09 | 3.2 |
| CXCL16, soluble | 1.1E-13 | 4.5E-10 | 3.6 |
| Cyclophilin A | 1.8E-13 | 9.7E-10 | 3.7 |
| Cystatin C | 5.4E-13 | 1.5E-08 | 4.4 |
| Cystatin M | 4.8E-13 | 7.7E-10 | 3.2 |
| Cystatin SN | 1.1E-12 | 6.4E-09 | 3.8 |
| Cytochrome c | 2.2E-12 | 3.1E-09 | 3.2 |
| DAN | 2.1E-12 | 4.8E-10 | 2.4 |
| DPP2 | 3.6E-12 | 3.1E-09 | 2.9 |
| ECM1 | 1.6E-13 | 2.0E-10 | 3.1 |
| EG-VEGF | 1.5E-12 | 8.8E-09 | 3.8 |
| eIF-4H | 8.6E-11 | 1.5E-08 | 2.2 |
| Endostatin | 1.3E-13 | 9.9E-10 | 3.9 |
| Eotaxin | 6.2E-11 | 2.5E-09 | 1.6 |
| Eotaxin-2 | 1.0E-12 | 1.0E-09 | 3.0 |
| EphA3 | 1.5E-12 | 1.1E-09 | 2.9 |
| Ephrin-A5 | 4.6E-13 | 2.0E-09 | 3.6 |
| Epo-R | 8.2E-11 | 5.5E-09 | 1.8 |
| ERBB1 | 3.8E-14 | 3.3E-10 | 3.9 |
| ERBB2 | 1.7E-11 | 1.6E-08 | 3.0 |
| ERBB3 | 2.9E-13 | 5.2E-10 | 3.2 |
| ERBB4 | 1.0E-11 | 2.7E-09 | 2.4 |
| ESAM | 2.9E-14 | 3.4E-10 | 4.1 |
| ETHE1 | 7.5E-13 | 1.8E-09 | 3.4 |
| Factor B | 9.4E-13 | 2.7E-08 | 4.5 |
| Factor D | 1.6E-12 | 9.5E-10 | 2.8 |
| Factor H | 6.8E-13 | 8.3E-09 | 4.1 |
| Factor I | 2.3E-14 | 4.5E-10 | 4.3 |
| Fas ligand, soluble | 6.8E-12 | 7.1E-09 | 3.0 |
| FCγ2A | 4.9E-13 | 1.1E-09 | 3.4 |
| Ferritin | 3.5E-11 | 1.0E-08 | 2.5 |
| Fetuin B | 6.9E-13 | 7.1E-11 | 2.0 |
| FGF-16 | 1.6E-10 | 4.6E-08 | 2.5 |
| FGF-18 | 1.0E-12 | 7.2E-10 | 2.8 |
| FGF-19 | 5.7E-13 | 7.9E-10 | 3.1 |
| FGF-20 | 7.9E-13 | 4.6E-10 | 2.8 |
| FGF-4 | 4.4E-13 | 1.5E-09 | 3.5 |
| FGF-6 | 6.1E-13 | 1.8E-09 | 3.5 |
| FGF-7 | 2.6E-13 | 9.8E-10 | 3.6 |
| FGF-9 | 2.0E-12 | 3.7E-09 | 3.3 |
| Fibrinogen | 4.2E-14 | 1.0E-09 | 4.4 |
| Fibronectin | 1.7E-12 | 2.4E-09 | 3.2 |
| Ficolin-2 | 9.6E-12 | 1.6E-08 | 3.2 |
| Fibronectin FN1.3 | 3.6E-14 | 1.3E-09 | 4.6 |
| Fibronectin FN1.4 | 1.0E-12 | 4.2E-09 | 3.6 |
| Fractalkine/CX3CL-1 | 2.9E-12 | 5.6E-10 | 2.3 |
| FSH | 3.6E-12 | 2.6E-09 | 2.9 |
| FSTL3 | 7.3E-14 | 3.5E-10 | 3.7 |
| GAPDH | 3.7E-13 | 1.1E-09 | 3.5 |
| GCP-2 | 8.7E-13 | 7.5E-10 | 2.9 |

Nature Precedings : hdl:10101/npre.2010.4538.1 : Posted 14 Jun 2010

| Target | Concentration (M) ($\sigma_{logRFU}$ Fit) | | |
|---|---|---|---|
| | LLOQ | ULOQ | log Range |
| GDF-11 | 1.2E-12 | 2.1E-10 | 2.2 |
| gp130, soluble | 9.4E-13 | 1.4E-09 | 3.2 |
| Granulysin | 2.1E-13 | 7.0E-10 | 3.5 |
| Granzyme A | 4.3E-14 | 3.9E-10 | 4.0 |
| Group IIA phospholipase A2 | 6.5E-13 | 4.6E-10 | 2.9 |
| Growth hormone receptor | 4.8E-12 | 7.4E-09 | 3.2 |
| Gro-α | 6.5E-13 | 1.4E-09 | 3.3 |
| Gro-γ | 9.5E-13 | 1.1E-09 | 3.1 |
| GSK-3 α | 1.0E-06 | 1.0E-06 | 0.0 |
| GSK-3 β | 2.2E-12 | 1.3E-08 | 3.8 |
| HAI-2 | 4.0E-13 | 6.3E-10 | 3.2 |
| Haptoglobin, Mixed Type | 1.9E-11 | 8.7E-08 | 3.7 |
| HCC-1 | 9.9E-13 | 4.3E-09 | 3.6 |
| HCC-4 | 1.1E-12 | 1.2E-09 | 3.0 |
| Hemopexin | 1.5E-12 | 1.0E-06 | 5.8 |
| Heparin cofactor II | 4.6E-13 | 3.0E-08 | 4.8 |
| Hepcidin-25 | 1.3E-12 | 1.5E-09 | 3.1 |
| HGF activator | 5.9E-14 | 7.5E-11 | 3.1 |
| HMG-1 | 1.4E-12 | 2.3E-09 | 3.2 |
| HSP 60 | 4.8E-13 | 5.9E-10 | 3.1 |
| HSP 70 | 9.6E-12 | 1.8E-10 | 1.3 |
| HSP 90α | 1.4E-12 | 1.2E-09 | 2.9 |
| HSP 90β | 3.5E-12 | 2.1E-09 | 2.8 |
| iC3b | 1.9E-13 | 9.0E-10 | 3.7 |
| ICOS | 1.1E-11 | 8.0E-09 | 2.9 |
| IFN-γ | 6.6E-13 | 4.2E-10 | 2.8 |
| IgE | 3.2E-13 | 2.3E-09 | 3.9 |
| IGFBP-1 | 2.1E-14 | 7.2E-10 | 4.5 |
| IGFBP-2 | 9.4E-13 | 2.4E-09 | 3.4 |
| IGFBP-3 | 6.6E-12 | 1.9E-09 | 2.5 |
| IGFBP-4 | 2.5E-13 | 2.0E-10 | 2.9 |
| IGFBP-5 | 3.1E-13 | 3.6E-10 | 3.1 |
| IGFBP-6 | 1.5E-13 | 1.1E-09 | 3.8 |
| IGFBP-7 | 7.4E-14 | 3.1E-10 | 3.6 |
| IGF-I | 3.8E-13 | 7.4E-10 | 3.3 |
| IGF-II receptor | 4.6E-12 | 2.0E-09 | 2.6 |
| IgM | 8.2E-12 | 4.0E-09 | 2.7 |
| IL-1 R4 | 4.1E-12 | 5.6E-10 | 2.1 |
| IL-10 | 2.5E-12 | 1.5E-09 | 2.8 |
| IL-11 | 6.3E-12 | 2.3E-08 | 3.6 |
| IL-12 | 1.4E-10 | 6.7E-09 | 1.7 |
| IL-13 | 9.9E-13 | 7.9E-10 | 2.9 |
| IL-15 Rα | 1.9E-13 | 2.4E-10 | 3.1 |
| IL-16 | 9.4E-14 | 7.7E-11 | 2.9 |
| IL-17 | 8.5E-13 | 3.3E-09 | 3.6 |
| IL-17B | 4.0E-13 | 3.3E-10 | 2.9 |
| IL-18 BPa | 1.4E-11 | 1.5E-09 | 2.0 |
| IL-2 | 1.7E-13 | 1.3E-09 | 3.9 |
| IL-2 sRγ | 5.1E-12 | 5.4E-09 | 3.0 |
| IL-4 | 2.7E-13 | 8.4E-10 | 3.5 |
| IL-4 sR | 2.4E-12 | 4.7E-09 | 3.3 |
| IL-6 | 1.3E-12 | 1.0E-09 | 2.9 |

Nature Precedings : hdl:10101/npre.2010.4538.1 : Posted 14 Jun 2010

Nature Precedings : hdl:10101/npre.2010.4538.1 : Posted 14 Jun 2010

| Target | Concentration (M) ($\sigma_{logRFU}$ Fit) | | |
|---|---|---|---|
| | LLOQ | ULOQ | log Range |
| IL-6 sRα | 3.0E-13 | 8.0E-10 | 3.4 |
| IL-8 | 7.3E-14 | 4.0E-10 | 3.7 |
| ING1 | 1.8E-12 | 3.9E-09 | 3.3 |
| Integrin α1β1 | 1.3E-11 | 7.9E-10 | 1.8 |
| I-TAC | 7.6E-13 | 5.8E-10 | 2.9 |
| Kallikrein 4 | 1.6E-11 | 8.3E-09 | 2.7 |
| Kallikrein 7 | 1.7E-12 | 5.6E-10 | 2.5 |
| Kallikrein 8 | 8.2E-12 | 6.7E-09 | 2.9 |
| Kallikrein 12 | 1.3E-12 | 6.1E-10 | 2.7 |
| Kallistatin | 7.4E-14 | 9.7E-10 | 4.1 |
| Kininogen, HMW, Single Chain | 8.4E-13 | 2.3E-10 | 2.4 |
| Lactoferrin | 1.4E-13 | 1.4E-09 | 4.0 |
| LAG-1 | 1.6E-13 | 5.5E-10 | 3.6 |
| Lamin-B1 | 7.8E-14 | 8.5E-10 | 4.0 |
| LBP | 2.6E-13 | 2.3E-10 | 2.9 |
| LD78-β | 7.2E-13 | 2.5E-09 | 3.5 |
| LDH-H 1 | 8.4E-13 | 3.9E-09 | 3.7 |
| Leptin | 2.4E-12 | 8.6E-09 | 3.6 |
| Leptin | 8.3E-14 | 1.1E-09 | 4.1 |
| Lipocalin 2 | 2.9E-13 | 1.6E-09 | 3.7 |
| LRIG3 | 1.2E-13 | 5.2E-10 | 3.6 |
| Luteinizing hormone | 3.7E-13 | 4.5E-09 | 4.1 |
| Lymphotactin | 2.7E-13 | 2.7E-10 | 3.0 |
| Lymphotoxin α1/β2 | 4.8E-13 | 1.7E-09 | 3.6 |
| Lymphotoxin α2/β1 | 4.3E-12 | 1.1E-08 | 3.4 |
| LYN A | 6.7E-13 | 3.2E-09 | 3.7 |
| LYVE-1 | 8.8E-13 | 7.0E-10 | 2.9 |
| Macrophage mannose receptor | 1.0E-11 | 6.6E-10 | 1.8 |
| MAPK14 | 2.6E-12 | 4.4E-09 | 3.2 |
| MAPK3 | 8.5E-13 | 3.4E-09 | 3.6 |
| MAPKAPK2 | 1.9E-11 | 8.4E-09 | 2.6 |
| MAPKAPK5 | 8.5E-11 | 8.4E-10 | 1.0 |
| MCP-1 | 5.4E-13 | 5.2E-10 | 3.0 |
| MCP-2 | 1.5E-12 | 9.8E-09 | 3.8 |
| MCP-3 | 2.1E-12 | 1.0E-08 | 3.7 |
| MCP-4 | 5.1E-12 | 6.1E-10 | 2.1 |
| M-CSF R | 5.1E-13 | 9.0E-10 | 3.2 |
| MD-1 | 1.6E-12 | 3.6E-08 | 4.3 |
| MDHC | 2.9E-13 | 2.0E-09 | 3.8 |
| Mesothelin | 3.0E-12 | 1.3E-09 | 2.6 |
| Mesothelin | 9.8E-12 | 9.8E-09 | 3.0 |
| MetAP 1 | 9.3E-13 | 2.1E-09 | 3.4 |
| MIA | 2.2E-13 | 1.7E-09 | 3.9 |
| Midkine | 4.0E-11 | 7.2E-09 | 2.3 |
| MIP-1α | 3.4E-12 | 5.8E-10 | 2.2 |
| MIP-1β | 6.8E-10 | 1.0E-06 | 3.2 |
| MIP-4 | 1.3E-13 | 3.6E-10 | 3.4 |
| MIP-5 | 2.4E-13 | 9.3E-10 | 3.6 |
| MMP-2 | 4.6E-12 | 1.2E-09 | 2.4 |
| MMP-3 | 3.1E-10 | 1.9E-08 | 1.8 |
| MMP-7 | 2.5E-13 | 1.0E-09 | 3.6 |
| MMP-8 | 1.7E-12 | 2.8E-09 | 3.2 |

Nature Precedings : hdl:10101/npre.2010.4538.1 : Posted 14 Jun 2010

| Target | Concentration (M) ($\sigma_{logRFU}$ Fit) | | |
|---|---|---|---|
| | LLOQ | ULOQ | log Range |
| MMP-9 | 5.8E-13 | 3.8E-09 | 3.8 |
| MMP-10 | 1.6E-12 | 1.8E-09 | 3.1 |
| MPIF-1 | 1.2E-13 | 5.2E-10 | 3.7 |
| MRC2 | 1.3E-13 | 3.3E-10 | 3.4 |
| Myeloperoxidase | 2.0E-13 | 1.3E-09 | 3.8 |
| Myoglobin | 2.4E-14 | 6.9E-10 | 4.5 |
| Myosin regulatory light chain 2 | 1.2E-11 | 2.9E-09 | 2.4 |
| NADPH-P450 Oxidoreductase | 8.0E-13 | 9.7E-10 | 3.1 |
| NAP-2 | 2.5E-14 | 2.3E-10 | 4.0 |
| Netrin-4 | 3.1E-13 | 5.6E-10 | 3.3 |
| Neurotrophin-3 | 1.9E-13 | 7.5E-10 | 3.6 |
| Neurotrophin-5 | 6.7E-13 | 6.9E-10 | 3.0 |
| Nidogen-2 | 1.4E-12 | 1.6E-08 | 4.1 |
| Noggin | 1.2E-11 | 4.4E-09 | 2.6 |
| NRP1 | 1.0E-14 | 2.2E-10 | 4.3 |
| OBCAM | 4.8E-11 | 1.0E-06 | 4.3 |
| RUNX-2 | 2.4E-13 | 7.2E-09 | 4.5 |
| Osteonectin | 1.0E-06 | 1.0E-06 | 0.0 |
| Otubain-1 | 5.4E-12 | 7.1E-08 | 4.1 |
| OX40 Ligand | 4.0E-12 | 7.6E-10 | 2.3 |
| PAI-1 | 2.8E-12 | 1.0E-06 | 5.6 |
| PAPP-A | 4.5E-13 | 6.1E-10 | 3.1 |
| P-Cadherin | 4.5E-12 | 2.6E-09 | 2.8 |
| Protein C Inhibitor | 1.1E-12 | 1.8E-09 | 3.2 |
| PCNA | 2.1E-12 | 1.8E-09 | 2.9 |
| PDGF-BB | 2.2E-13 | 6.5E-10 | 3.5 |
| PF-4 | 6.2E-14 | 8.8E-10 | 4.2 |
| PGRP-S | 4.4E-14 | 8.9E-10 | 4.3 |
| PKC-ζ | 2.7E-11 | 2.8E-08 | 3.0 |
| Plasminogen | 1.3E-12 | 6.3E-09 | 3.7 |
| PLPP | 3.0E-12 | 5.8E-09 | 3.3 |
| Prekallikrein | 3.5E-13 | 1.2E-09 | 3.5 |
| Properdin | 1.5E-12 | 2.2E-08 | 4.2 |
| Protease nexin I | 1.1E-12 | 3.7E-09 | 3.5 |
| Proteasome subunit p40 | 5.3E-12 | 1.3E-08 | 3.4 |
| Protein C | 8.2E-13 | 2.0E-08 | 4.4 |
| Protein S | 1.4E-12 | 5.9E-10 | 2.6 |
| Proteinase-3 | 3.6E-12 | 1.3E-09 | 2.6 |
| Prothrombin | 1.4E-12 | 1.0E-08 | 3.9 |
| PSA | 1.3E-12 | 1.6E-09 | 3.1 |
| PSA-ACT | 6.6E-14 | 5.4E-10 | 3.9 |
| P-Selectin | 1.0E-14 | 4.0E-10 | 4.6 |
| pTEN | 1.7E-12 | 1.6E-09 | 3.0 |
| RANTES | 7.0E-12 | 3.1E-09 | 2.7 |
| RBP | 9.1E-11 | 7.7E-08 | 2.9 |
| Renin | 2.6E-13 | 4.8E-10 | 3.3 |
| resistin | 1.4E-12 | 7.6E-10 | 2.7 |
| RGM-B | 1.9E-13 | 3.5E-10 | 3.3 |
| ROR1 | 3.7E-13 | 2.4E-09 | 3.8 |
| RPS6Kα3 | 4.1E-13 | 5.0E-10 | 3.1 |
| S100A12 | 1.0E-06 | 1.0E-06 | 0.0 |
| SAP | 3.2E-13 | 1.8E-09 | 3.8 |

Nature Precedings : hdl:10101/npre.2010.4538.1 : Posted 14 Jun 2010

| Target | Concentration (M) ($\sigma_{logRFU}$ Fit) | | |
|---|---|---|---|
| | LLOQ | ULOQ | log Range |
| SCF sR | 3.1E-12 | 5.1E-10 | 2.2 |
| sE-Selectin | 1.1E-13 | 1.4E-10 | 3.1 |
| ICAM-2, soluble | 8.9E-11 | 8.9E-09 | 2.0 |
| ICAM-3, soluble | 2.9E-12 | 9.5E-09 | 3.5 |
| Siglec-9 | 6.0E-14 | 1.8E-10 | 3.5 |
| SLPI | 9.3E-13 | 4.8E-09 | 3.7 |
| sL-Selectin | 2.0E-13 | 1.4E-09 | 3.8 |
| Sonic Hedgehog | 1.1E-13 | 2.3E-09 | 4.3 |
| Sorting nexin 4 | 7.4E-13 | 2.5E-09 | 3.5 |
| sTie-1 | 1.4E-12 | 4.2E-10 | 2.5 |
| STK16 | 3.4E-12 | 7.5E-10 | 2.3 |
| tau | 1.2E-10 | 3.2E-08 | 2.4 |
| Tenascin | 6.7E-12 | 1.2E-09 | 2.3 |
| TFPI | 4.2E-14 | 6.4E-10 | 4.2 |
| TGF-β1 | 2.4E-12 | 5.7E-09 | 3.4 |
| TGF-β2 | 1.0E-12 | 3.4E-09 | 3.5 |
| TGF-β3 | 9.6E-12 | 6.9E-09 | 2.9 |
| Thrombin | 6.5E-13 | 1.1E-09 | 3.2 |
| Thrombospondin-4 | 1.6E-13 | 1.9E-09 | 4.1 |
| Thyroid peroxidase | 1.8E-13 | 1.2E-09 | 3.8 |
| Thyroxine-Binding Globulin | 1.4E-13 | 4.6E-10 | 3.5 |
| TIMP-1 | 1.6E-13 | 2.0E-09 | 4.1 |
| TIMP-2 | 6.4E-11 | 6.8E-08 | 3.0 |
| TIMP-3 | 2.3E-13 | 1.6E-09 | 3.8 |
| TNF sR-I | 5.6E-13 | 1.2E-09 | 3.3 |
| TNF sR-II | 2.8E-12 | 2.1E-09 | 2.9 |
| TNFSF18 | 8.7E-13 | 8.0E-10 | 3.0 |
| tPA | 7.4E-12 | 7.0E-10 | 2.0 |
| TRAIL R4 | 4.7E-12 | 3.3E-10 | 1.8 |
| Transferrin | 1.9E-12 | 4.7E-09 | 3.4 |
| TrATPase | 1.4E-13 | 1.3E-09 | 4.0 |
| Troponin I | 1.7E-11 | 4.2E-09 | 2.4 |
| Troponin T | 5.0E-11 | 3.4E-09 | 1.8 |
| TSLP | 1.0E-12 | 1.4E-09 | 3.1 |
| UBC9 | 2.6E-14 | 3.8E-10 | 4.2 |
| Ubiquitin+1 | 1.2E-12 | 1.7E-09 | 3.2 |
| URB | 3.8E-13 | 3.7E-10 | 3.0 |
| VCAM-1 | 4.9E-12 | 1.1E-08 | 3.4 |
| VEGF | 9.4E-14 | 3.9E-09 | 4.6 |
| VEGF sR2 | 3.8E-13 | 1.1E-09 | 3.5 |
| VEGF sR3 | 8.3E-13 | 2.1E-09 | 3.4 |
| vWF | 1.1E-11 | 8.0E-10 | 1.9 |
| WIF-1 | 1.2E-12 | 1.4E-09 | 3.1 |
| WISP-1 | 2.9E-12 | 5.7E-10 | 2.3 |
| X-Pro aminopeptidase 1 | 2.4E-12 | 3.7E-09 | 3.2 |

Table 6. Limits of quantification for spiked proteins

| Target | Concentration (M) ($\sigma_{logRFU}$ Fit) | | | | | |
|---|---|---|---|---|---|---|
| | Plasma | | | Buffer | | |
| | LLOQ | ULOQ | log Range | LLOQ | ULOQ | log Range |
| Activin A | 3.6E-12 | 1.1E-09 | 2.5 | 6.5E-13 | 7.5E-10 | 3.1 |
| ADAMTS-4 | 1.0E-12 | 5.1E-10 | 2.7 | 1.1E-13 | 2.8E-10 | 3.4 |
| CNTF | 3.6E-13 | 7.1E-10 | 3.3 | 9.4E-14 | 2.2E-10 | 3.4 |
| CTLA-4 | 3.1E-12 | 6.4E-09 | 3.3 | 1.9E-12 | 3.8E-09 | 3.3 |
| EG-VEGF | 1.8E-12 | 2.8E-09 | 3.2 | 2.4E-12 | 3.0E-09 | 3.1 |
| Ephrin-A5 | 3.0E-12 | 1.6E-09 | 2.7 | 1.0E-12 | 5.2E-10 | 2.7 |
| FGF-4 | 1.5E-11 | 1.7E-08 | 3.1 | 7.7E-12 | 1.1E-08 | 3.1 |
| FGF-6 | 3.8E-12 | 2.9E-09 | 2.9 | 4.8E-12 | 9.9E-10 | 2.3 |
| FGF-9 | 1.1E-10 | 9.0E-09 | 1.9 | 3.7E-11 | 6.3E-09 | 2.2 |
| FGF-20 | 1.7E-12 | 6.5E-10 | 2.6 | 1.1E-12 | 2.7E-10 | 2.4 |
| Granzyme A | 5.2E-13 | 6.5E-10 | 3.1 | 1.6E-13 | 4.3E-10 | 3.4 |
| HAI-2 | 5.5E-13 | 8.7E-10 | 3.2 | 3.3E-13 | 2.7E-10 | 2.9 |
| IL-2 | 9.2E-13 | 1.4E-09 | 3.2 | 3.8E-13 | 6.2E-10 | 3.2 |
| IL-4 | 6.3E-13 | 8.4E-10 | 3.1 | 1.1E-12 | 2.0E-10 | 2.2 |
| IL-11 | 5.1E-12 | 1.0E-06 | 5.3 | 7.6E-12 | 5.1E-08 | 3.8 |
| IL-17B | 1.3E-12 | 1.8E-09 | 3.2 | 7.0E-12 | 5.5E-10 | 1.9 |
| IL-4 sR | 1.6E-11 | 5.9E-09 | 2.6 | 3.4E-12 | 6.5E-09 | 3.3 |
| I-TAC | 4.1E-12 | 1.4E-09 | 2.5 | 2.2E-12 | 8.8E-10 | 2.6 |
| Lymphotoxin α1/β2 | 6.4E-12 | 2.8E-08 | 3.6 | 1.7E-12 | 7.5E-09 | 3.6 |
| MCP-2 | 9.3E-12 | 5.5E-09 | 2.8 | 3.0E-12 | 2.2E-09 | 2.9 |
| MCP-3 | 1.7E-11 | 7.7E-09 | 2.7 | 1.5E-11 | 6.6E-09 | 2.7 |
| Neurotrophin-3 | 6.5E-13 | 8.4E-10 | 3.1 | 1.4E-12 | 4.9E-10 | 2.5 |
| PSA | 3.3E-11 | 1.0E-08 | 2.5 | 2.4E-12 | 1.7E-09 | 2.8 |
| Sonic Hedgehog | 1.5E-12 | 1.3E-09 | 2.9 | 1.4E-12 | 5.4E-10 | 2.6 |
| TGF-β2 | 1.6E-12 | 1.0E-09 | 2.8 | 1.8E-12 | 7.4E-10 | 2.6 |
| TNF sR-I | 5.2E-12 | 1.0E-09 | 2.3 | 1.6E-12 | 7.3E-10 | 2.7 |
| TNF sR-II | 2.9E-12 | 6.5E-09 | 3.4 | 3.5E-12 | 1.3E-08 | 3.6 |
| TNFSF18 | 2.3E-12 | 8.3E-10 | 2.6 | 9.1E-13 | 4.3E-10 | 2.7 |

Nature Precedings : hdl:10101/npre.2010.4538.1 : Posted 14 Jun 2010
